# Synthesis and Evaluation of Prodrugs of α-Carboxy Nucleoside Phosphonates

**DOI:** 10.1021/acs.joc.2c02135

**Published:** 2022-10-25

**Authors:** Alan Ford, Nicholas D. Mullins, Jan Balzarini, Anita R. Maguire

**Affiliations:** † School of Chemistry, Analytical and Biological Chemistry Research Facility, Synthesis and Solid State Pharmaceutical Centre, University College Cork, Cork T12 K8AF, Ireland; ‡ Rega Institute for Medical Research, KU Leuven, Herestraat 49, B-3000 Leuven, Belgium; § School of Pharmacy, Analytical and Biological Chemistry Research Facility, Synthesis and Solid State Pharmaceutical Centre, University College Cork, Cork T12 K8AF, Ireland

## Abstract

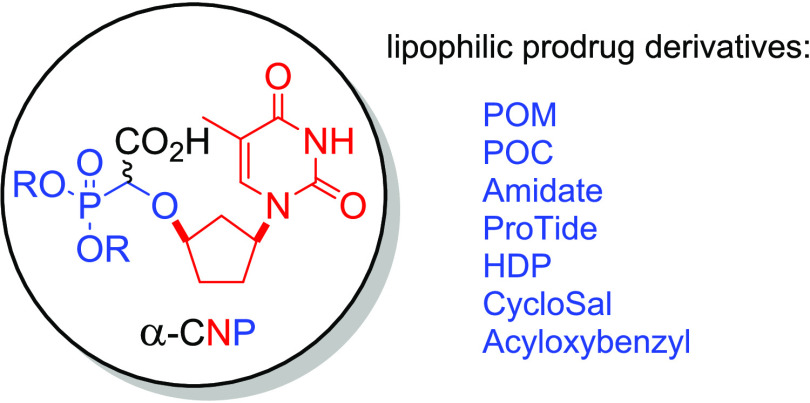

A range of lipophilic prodrugs of α-carboxy nucleoside phosphonates, potent inhibitors of HIV-1 reverse transcriptase without requiring prior phosphorylation, were synthesized to evaluate their in vivo potency against HIV in cell culture. A series of prodrug derivatives bearing a free carboxylic acid where the phosphonate was masked with bispivaloyloxymethyl, diisopropyloxycarbonyloxymethyl, bisamidate, aryloxyphosphoramidate, hexadecyloxypropyl, CycloSal, and acycloxybenzyl moieties were synthesized, adapting existing methodologies for phosphonate protection to accommodate the adjacent carboxylic acid moiety. The prodrugs were assayed for anti-HIV activity in CEM cell cultures—the bispivaloyloxymethyl free acid monophosphonate prodrug exhibited some activity (inhibitory concentration-50 (IC_50_) 59 ± 17 μM), while the other prodrugs were inactive at 100 μM. A racemic bispivaloyloxymethyl methyl ester monophosphonate prodrug was also prepared to assess the suitability of the methyl ester as a carboxylic acid prodrug. This compound exhibited no activity against HIV in cellular assays.

## Introduction

**Figure 1:**
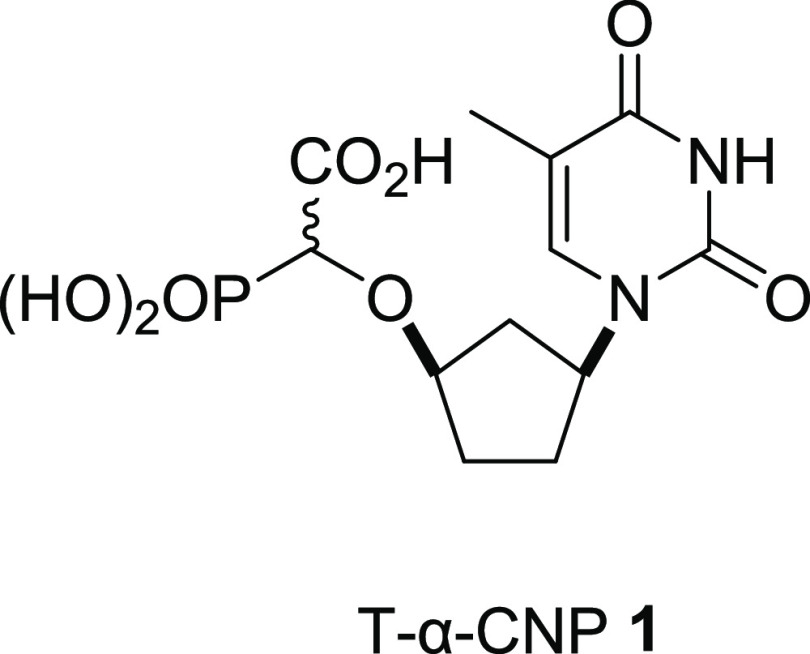
α-Carboxy nucleoside phosphonate (α-CNP) **1**.

Recently, we described the design, synthesis, and evaluation of a novel class of α-carboxy nucleoside phosphonates (α-CNPs), e.g., **1** (Figure 1), which are potent inhibitors of HIV-1 reverse transcriptase (RT) in cell-free assays.^1−5^ In addition, this class displays activity against a range of viral polymerases including Herpes virus DNA polymerases.^4^ Significantly, these phosphonates do not require phosphorylation to exhibit activity, in sharp contrast to other phosphononucleosides, which have been designed as nucleoside monophosphate mimics and thus require further phosphorylation to the triphosphate in the host cells before they can be active.^6^ We have demonstrated that the presence of the phosphonoacetic acid moiety is crucial to their activity,^7^ and indeed structural biology investigations have shown that the phosphonoacetic acid moiety binds to the metal site in HIV-1 RT mimicking the triphosphate binding in the natural nucleotide triphosphates.^2^ However, these molecules, containing both the phosphonic acid and carboxylic acid moieties, are too polar to efficiently migrate across the cell membrane as they are ionized at physiological pH.

**Figure 2:**
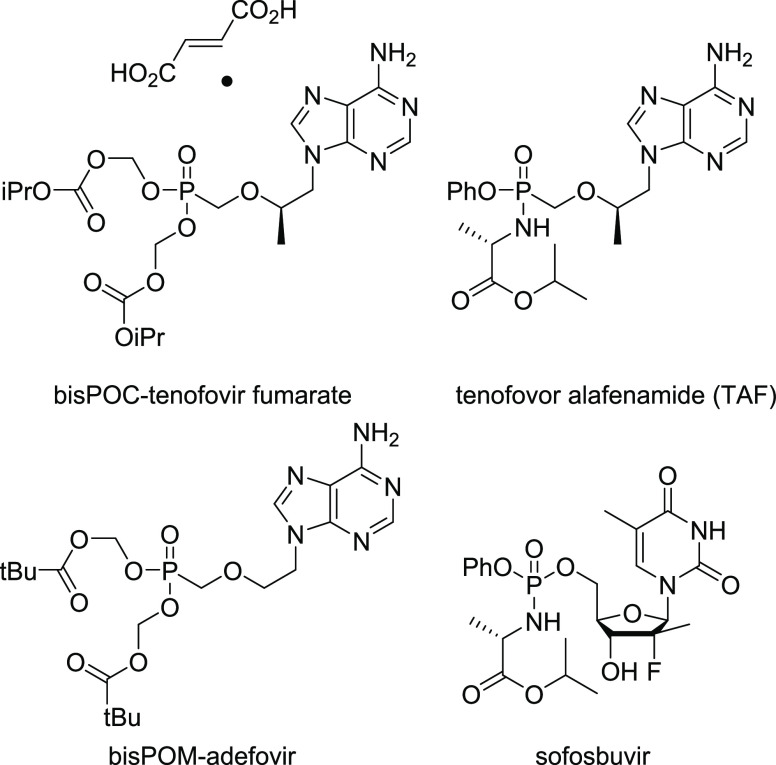
Clinically approved phosph(on)ate prodrugs.

There has been intense activity in the last few decades to develop prodrugs for the phosph(on)ate moiety to facilitate transport of these highly charged molecules into the cell. A number of recent accounts document the tremendous growth in this field.^8−11^ In spite of the efforts expended, to date, the only monophosphonate prodrugs clinically approved for HIV treatment are the diisopropyloxycarbonyloxymethyl (POC) ester of tenofovir fumarate and the alafenamide derivative of tenofovir (TAF).^12−15^ The bispivaloyloxymethyl (POM) prodrug of adefovir has been approved for the treatment of hepatitis B virus (HBV) infection (Figure 2).^16,17^

Naturally, these two prodrug approaches (POM, POC) which have well-documented clinical efficacy aroused our interest when seeking viable lipohilic forms for α-CNPs. Other strategies such as the ProTide (aryloxyphosphoramidate) approach developed by McGuigan,^18−23^ utilized in the prodrug Sofosbuvir (Figure 2),^24^ and the related bisamidate prodrugs,^25^ which release innocuous amino acids as byproducts, were also attractive targets. The highly lipophilic alkoxyalkyl prodrugs [e.g., hexadecyloxypropyl (HDP)] pioneered by Hostetler were also considered as potential protecting groups for the phosphonate moiety of the α-CNPs.^26−32^ Additionally, the cyclic, salicyl alcohol-derived “CycloSal” prodrugs described by Meier were of particular interest given that the CycloSal moiety undergoes a pH-dependent chemical hydrolysis to unmask the phosphonate rather than enzymatic hydrolysis; the stability of the CycloSal prodrugs can be altered by modification of substituents on the aromatic ring.^33−39^ A further prodrug moiety of interest, also developed by Meier, was the acyloxybenzyl phosphonate. Acyloxybenzyl prodrugs have been used with nucleoside phosphates,^40^ diphosphates,^41,42^ and triphosphates,^43^ and also with phosphonates.^44,45^ In this case, the deprotection of the phosphonate function is triggered by enzymatic hydrolysis of the acyl group, which is remote from the carboxyphosphonate, followed by spontaneous loss of the resulting hydroxybenzyl groups to reveal the free phosphonate.

**Figure 3:**
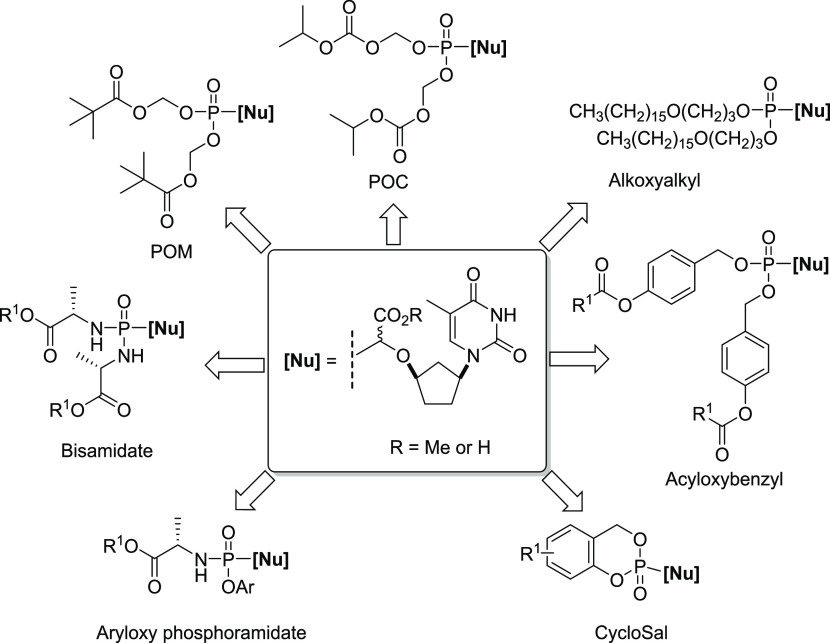
Potential target monophosphonate prodrugs of α-CNPs.

Most of the above approaches rely on enzymatic hydrolysis of the lipophilic phosphonate esters once they are within the cell to release the phosphonates. However, the majority of this work was developed utilizing phosphonates with an unsubstituted methylene linking the phosphorous and oxygen. Hence, one of the key issues to be addressed was whether the phosphonate esters of the α-CNPs would undergo enzymatic hydrolysis or if the efficiency of release of the free phosphonate would be affected by the presence of the carboxylic acid moiety at this carbon. Accordingly, Meier’s CycloSal protecting strategy was of particular interest. The various prodrug approaches are summarized in Figure 3.

In addition to protecting the polar phosphonate moiety, protecting the carboxylic acid was also briefly explored, to enhance the lipophilicity of the highly polar α-CNPs. Prodrugs for carboxylic acids have not been as widely documented, with POM-derivatized prodrugs being the most widely known (e.g., Pivampicillin).^46^ More recent advances in this area have been the development of the dioxolenone moiety reported by Cheng and co-workers^47^ and the *N*-alkyl-*N*-alkyloxycarbonylaminomethyl (NANAOCAM) derivatives of carboxylic acids described by Majumdar.^48^

## Results and Discussion

Our investigations of the prodrug strategies mostly utilized the racemic thymine α-CNP **1**, which displayed excellent inhibitory activity against HIV-RT, as a model compound. While we have demonstrated that the activity in the α-CNPs resides predominantly in the L-series,^1,2^ for the prodrug investigations, we employed the racemic compounds. As with our original α-CNPs, each of the compounds is generated as an equimolar mixture of diastereomers at the center adjacent to the phosphonate. Extension to other α-CNPs can be envisaged building on the methodologies developed below.

The principal synthetic challenge was to modify the established protocols to generate lipophilic phosphonate prodrugs for use with the α-CNPs bearing the α carboxylic acid function. As the inclusion of the carboxylic acid moiety adjacent to the phosphonate is not very common, existing prodrug strategies have not been developed to take on board the potential impact of the carboxylic acid moiety on either the introduction of the lipophilic group or the hydrolysis of the prodrug in vivo to release the active compound—both are likely to be impacted by the reactive carboxylic acid proximal to the phosphonate. A small number of prodrug derivatives of phosphonoacetates^49^ and other phosphono-carboxylic acid compounds^50^ have been described in recent years.

### Synthesis and Evaluation of Bis-POM Methyl Ester Monophosphonate Prodrug **2**

**Scheme 1:**
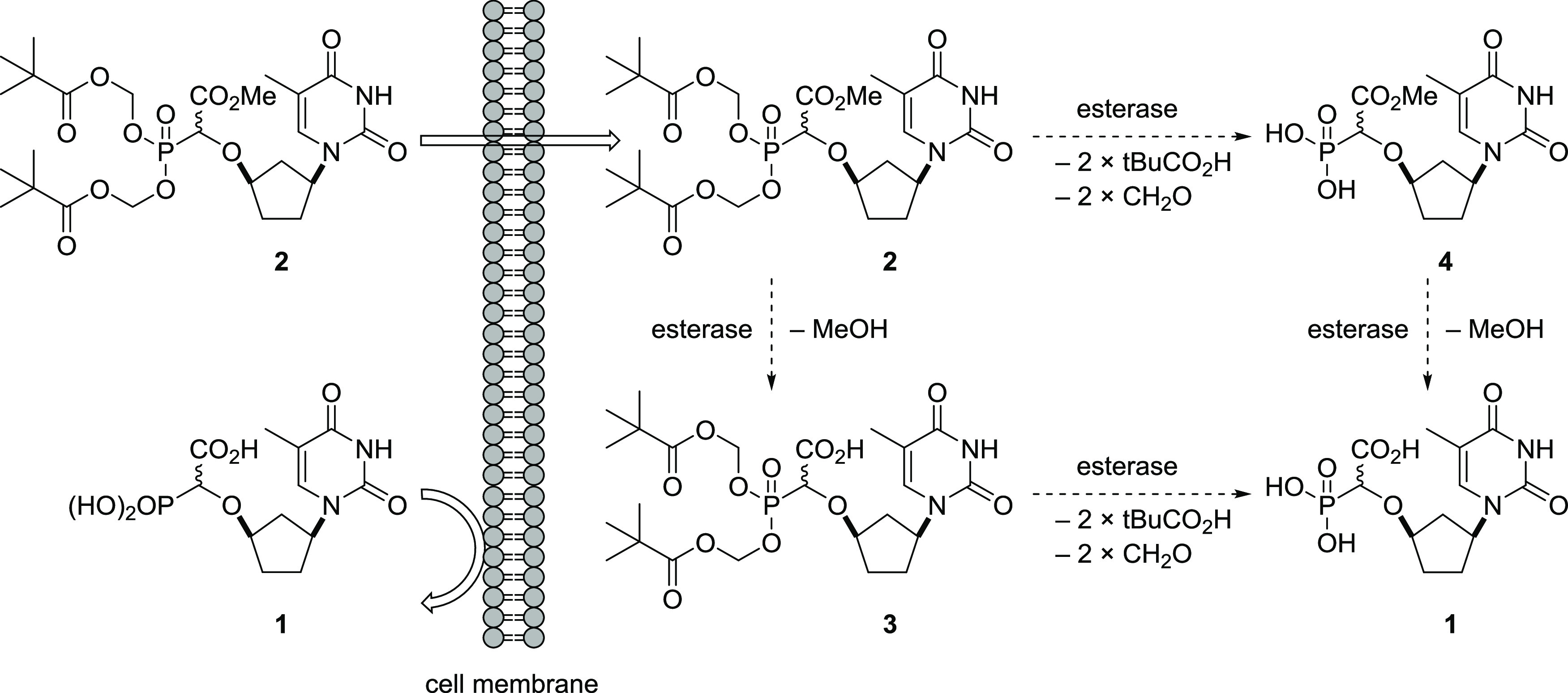
Potential Cellular Activation Pathway of **2** to Release **1**

Our initial focus centered on compound **2**, the bis-POM methyl ester monophosphonate prodrug of **1**. Because of the ubiquitous presence of esterases in blood, tissues, and organs, we were hopeful that the bis-POM prodrug **2** would be sufficiently lipophilic to penetrate the cell membrane whereupon the methyl ester would undergo hydrolysis by esterases to give **3**. The pivaloyloxymethyl moiety could then detach to unmask the phosphonate to give the fully deprotected α-CNP **1** (Scheme 1).^51^ It may also be possible that the activation steps are reversed i.e., the POM moieties are first hydrolyzed to generate **4** and subsequent methyl ester hydrolysis affords **1**. In parallel, investigation of the free acid prodrug **3** was envisaged, to obviate the necessity for methyl ester hydrolysis to release **1** inside the cell, with the expectation that **3** would still be sufficiently lipophilic to pass into cells.

**Scheme 2:**
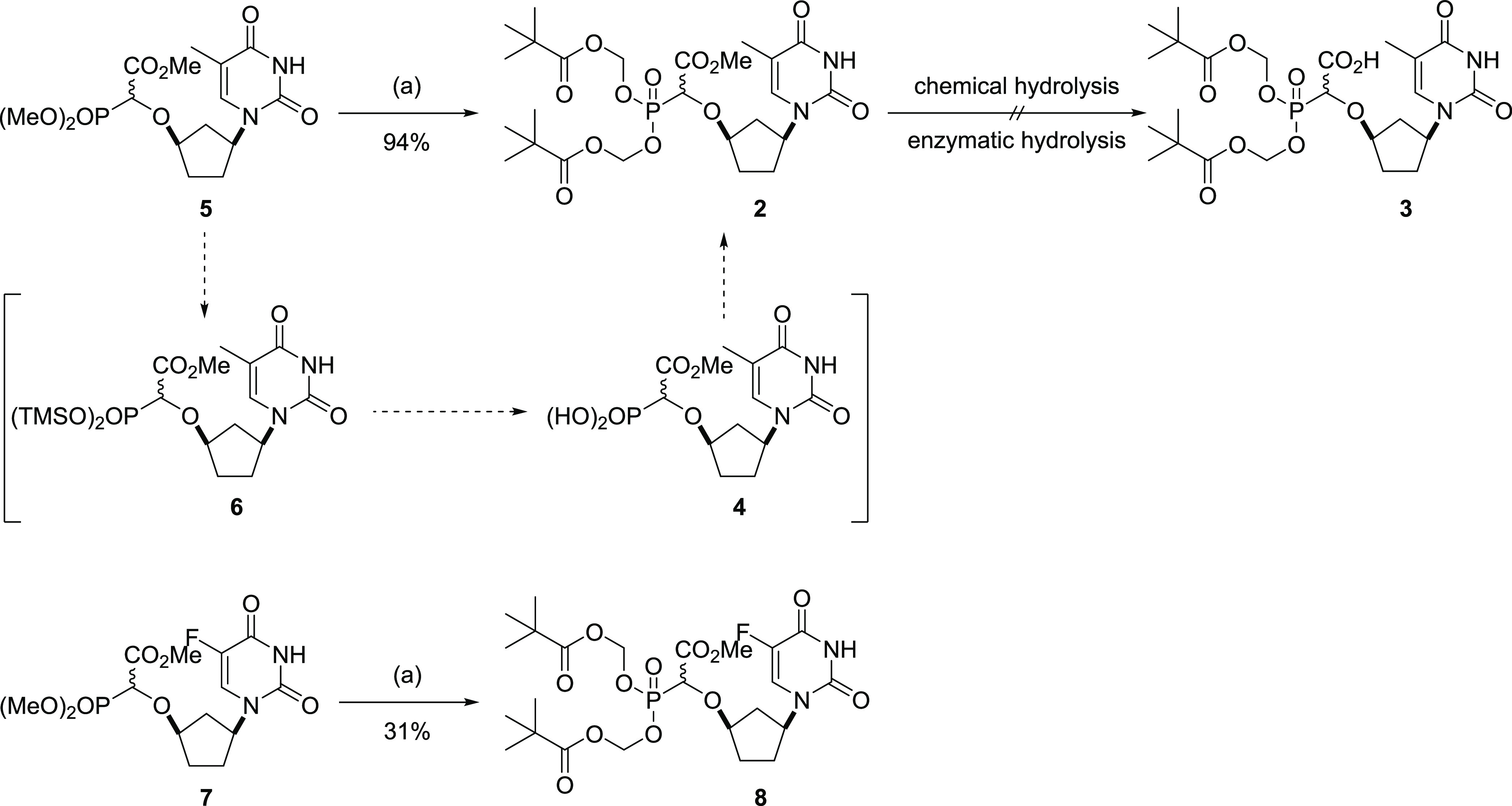
Synthesis of Bis-POM Methyl Ester Prodrugs **2** and **8** Conditions: (a) (i) TMSBr, MeCN microwave 50 °C, 10 min, (ii) MeOH/H_2_O, 30 min, (iii) POM-I, *N*,*N*-diisopropylethylamine (DIPEA), THF, 24 h.

The synthesis of compound **5**, a key intermediate in the production of α-CNPs has already been described by us.^1^ Microwave-mediated hydrolysis of **5** in the presence of trimethylsilyl bromide^1^ generated the TMS-phosphonate **6**, which was subsequently hydrolyzed in situ by stirring in methanol–water for 30 min to give **4**, which was reacted directly with POM iodide and Hünig’s base in tetrahydrofuran (THF) to furnish the racemic bis-POM target **2** in 94% yield from **5** after chromatography. When POM chloride was employed in place of POM iodide, much lower yields were obtained. A similar approach was employed to convert the 5-fluorouracil CNP analogue **7** to the bis-POM derivative **8** (Scheme 2).

Compound **2** was found to be inactive against HIV-1 in cellular assays, indicating a need to make prodrugs with the free carboxylic acid to avoid the need for multiple enzymatic hydrolyzes to release the α-CNPs. Attempts to selectively hydrolyze the methyl ester of **2** while preserving the prodrug appendages by both chemical and enzymatic (using commercially available esterases) means to isolate **3** were unsuccessful.

### Synthesis of Free Acid Prodrugs

**Scheme 3:**
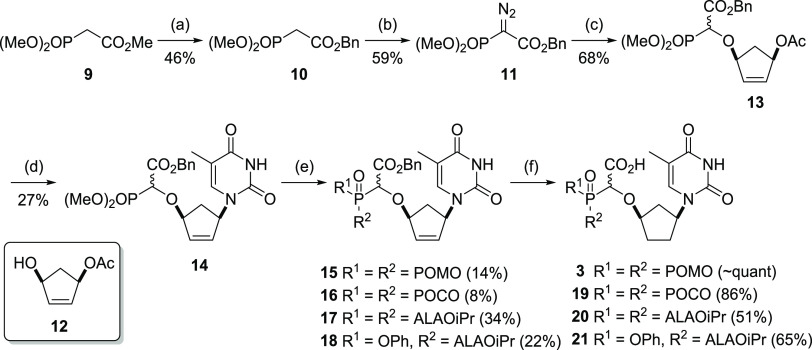
Synthesis of Bis-POM, Bis-POC, Bisamidate, and Aryloxyamidate Prodrugs Conditions: (a) (i) K_2_CO_3_, H_2_O, 24 h; (ii) BnBr, MeCN, 60 °C, 48 h; (b) 4-dodecylbenzenesulfonyl azide (DBSA), Et_3_N, MeCN, 48 h; (c) **12**, Rh_2_(OCCMe_3_)_4_, CH_2_Cl_2_, 40 °C, 24 h; (d) thymine, Pd(dba)_2_, dppb, Na_2_CO_3_, aq. MeCN, microwave 50 °C, 30 min; (e) for **15** (i) TMSBr, lutidine, MeCN microwave 50 °C, 10 min; (ii) MeOH/H_2_O, 30 min; (iii) POM-I, DIPEA, THF; 24 h; for **16**: (i) TMSBr, lutidine, MeCN microwave 50 °C, 10 min; (ii) MeOH/H_2_O, 30 min; (iii) POC-I, DIPEA, THF, 24 h; for **17**: (i) TMSBr, lutidine, MeCN microwave 50 °C, 10 min; (ii) 2,2-dithiodipyridine (Aldrithiol-2), l-ALA-OiPr, PPh_3_, pyridine, 90 °C, 24 h; for **18**: (i) TMSBr, lutidine, MeCN microwave 50 °C, 10 min; (ii) Aldrithiol-2, l-ALA-OiPr, PhOH, PPh_3_, pyridine, 90 °C, 24 h; (f) H_2_, 5% Pd/C 1 atm, MeOH, 24 h.

Undeterred, we revisited our strategy for making “free acid” prodrugs of **1**, preferring early introduction of a benzyl ester in the sequence, which would allow a late-stage unmasking of the carboxylic acid by hydrogenolysis (Scheme 3). We were hopeful that the hydrophobic prodrug attachments would make the α-CNP sufficiently lipophilic to transport into the cell, despite the presence of the free carboxylic acid.

Thus, commercially available **7** was converted in two steps to **11** via the dimethyl benzyl ester **10**.^52^ Diazo transfer to **10** was achieved using 4-dodecylbenzenesulfonyl azide (DBSA) to give the diazo phosphonoester **11** (27% yield over two steps).^53^ Use of DBSA for the diazo transfer was preferable to use of 4-acetamidobenzenesulfonylazide (ABSA) due to the ease of separating the reaction products by chromatography (the sulfonamide byproduct of ABSA co-elutes with **11**). We then employed a rhodium-catalyzed O–H insertion reaction, which we have developed for the synthesis of α-CNPs and related compounds,^1,7,54^ to access the allylic acetate **13** in 68% yield. In an improvement over our previously reported conditions, this reaction was carried out using rhodium pivalate, in dichloromethane (DCM) rather than benzene. Replacement of the methyl ester with the benzyl ester had only a modest impact on the efficiency of the key O–H insertion step. Introduction of the nucleobase thymine to **13** was facilitated by a palladium-catalyzed Tsuji–Trost reaction.^55^ Thus, an aqueous acetonitrile solution of the sodium salt of thymine was treated with a degassed solution of **13**, bis(dibenzylideneacetone)palladium(0) Pd(dba)_2_ and bis(diphenylphosphino)butane (dppb) in acetonitrile under microwave irradiation to generate the insertion product **14** in 27% yield. The benzyl dimethyl phosphonate ester **14** is a key intermediate in this sequence as it allows access to several of the “free acid” prodrugs. In contrast to the saturated phosphonate **5**, the amine base lutidine is required along with TMSBr to effect selective hydrolysis of the phosphonate esters of **14** which have an alkene present in the ring.^1^ Bis-POM modification was carried out as described earlier for **5** to give the bis-POM benzyl ester compound **15** in 14% yield following chromatography. Finally, concomitant hydrogenation of the cyclopentyl alkene and hydrogenolysis of the benzyl ester of **15** was achieved under a balloon of hydrogen in the presence of 5% Pd/C to furnish the racemic bis-POM prodrug **3** bearing the free carboxylic acid moiety. Under similar conditions, using POC-iodide, the bis-POC prodrug **19** was likewise prepared in two steps from **14**.

We were also able to prepare **15** and **16** from **14** in a convenient one-pot reaction.^56^ Heating a solution of **14**, sodium iodide and POM-Cl or POC-Cl in acetonitrile in a sealed tube for 48 h gave **15** (46%) and **16** (36%) respectively, after chromatography. This method is preferable since it does not require the hydrolysis and isolation of the intermediate phosphonic acid which can be time-consuming and labor-intensive; additionally, this reaction sequence proceeds using the chlorides of the carbonyloxymethyl reagents, thus the additional steps to prepare the analogous iodides are not necessary.

The bisamidate prodrug **20** was also synthesized in two steps from **14**. Heating the intermediate TMS-phosphonate prepared from **14** with L-Ala-O^*i*^Pr in the presence of 2,2-dithiodipyridine (Aldrithiol-2) and triphenylphosphine in pyridine at 90 °C gave the bis-substituted product **17** in 34% yield, which is in line with the modest yields reported in the literature.^25^ As before, simultaneous saturation of the alkene and hydrogenolysis of the ester using Pd/C under a balloon of hydrogen was carried out to afford the free acid bisamidate prodrug **20**. The phenoxyamidate **18** was prepared in a similar manner to **17** except that phenol was added along with l-Ala-O^*i*^Pr (**14**/phenol/Ala 1:4.8:2.1 ratio) in the reaction of the TMS derivative of **14**. Hydrogenation/hydrolysis of **18** gave the phenoxyamidate monophosphonate prodrug **21** in 65% yield, as a complex diastereomer mixture. When the phenoxyamidate reaction was carried out on a fivefold scale (**14**/phenol/Ala 1:5.1:2.0 ratio) it was possible to isolate both **17** (7%) and **18** (21%) by chromatography.

**Figure 4:**
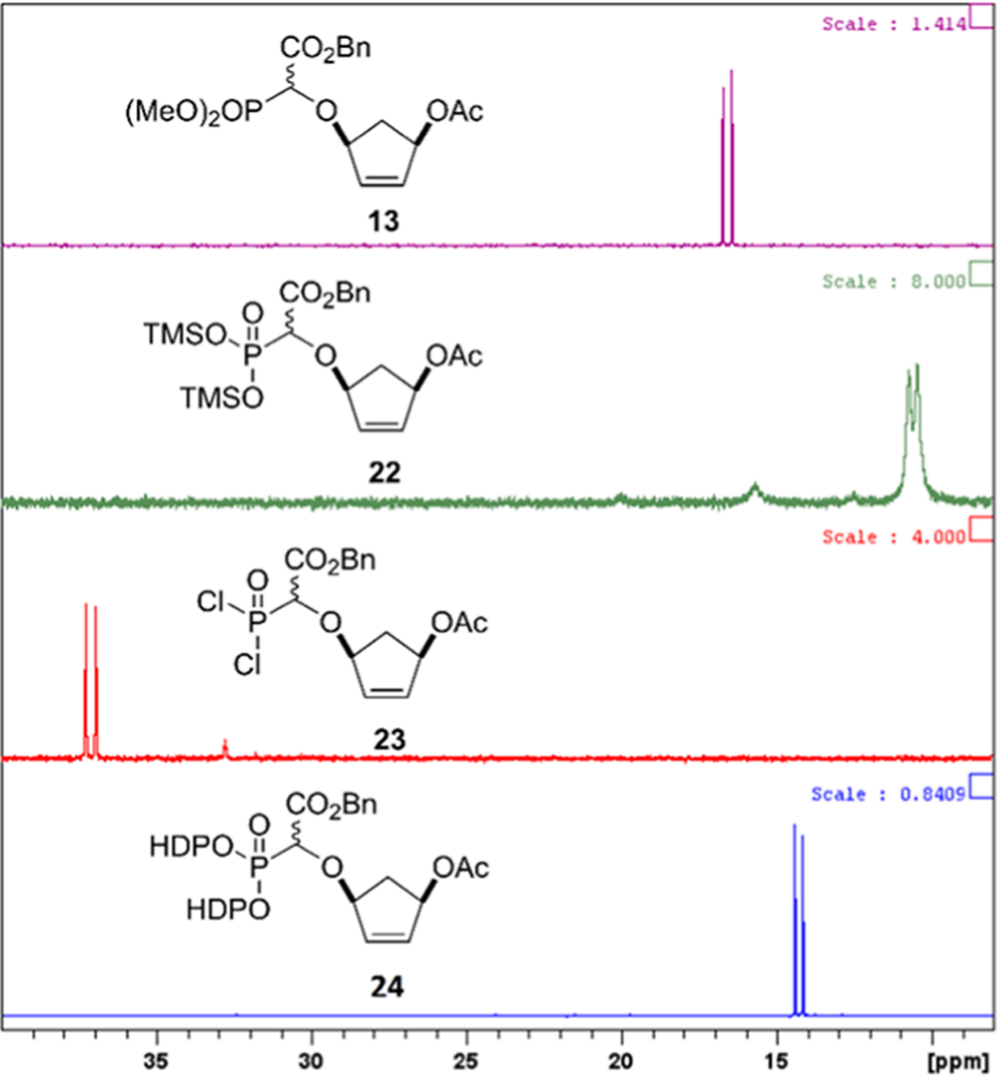
^31^P{^1^H} NMR (121.5 MHz, CDCl_3_) of conversion of **13** to **24** via TMS-phosphonate **22** and dichloridate **23**.

**Scheme 4:**
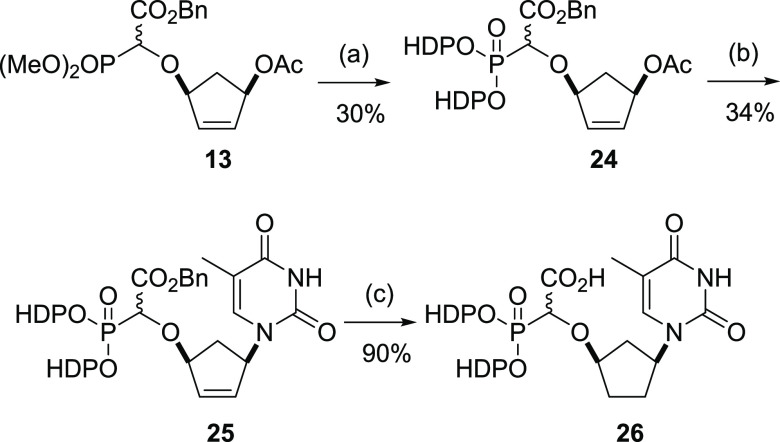
Synthesis of Bis(hexadecyloxypropyl) Prodrug Conditions: (a) (i) TMSBr, lutidine, MeCN microwave 50 °C, 10 min; (ii) (COCl)_2_, cat. DMF, DCM, room temperature (rt), 3 h; (iii) HDPOH, *i*Pr_2_NEt, 0 °C, DCM, 24 h; (b) thymine, Pd_2_(dba)_3_, dppb, Na_2_CO_3_, aq. MeCN, microwave 50 °C, 30 min; (c) H_2_, 5% Pd/C 1 atm, MeOH, 24 h (HDP = CH_3_(CH_2_)_15_O(CH_2_)_3_−).

We had initially also hoped to install the bis-HDP moiety immediately after the base insertion step (i.e., from compound **14**), however, several attempts following literature procedures were unsuccessful. Thus, we resolved to introduce the HDP group earlier in the sequence to overcome this issue (Scheme 4). Reaction of the O–H insertion product **13** with TMSBr and lutidine under microwave conditions generated the TMS-phosphonate **22** which was not isolated, but treated with oxalyl chloride and dimethylformamide (DMF) (cat.) in DCM. Evaporation of the solvent gave the dichloridate **23** which in the presence of HDPOH and diisopropylethylamine at 0 °C gave the desired bis-HDP phosphonate **24** in 30% yield following chromatography.^57^ The conversion of **13** to **24** via the TMS-phosphonate **22** and dichloridate **23** could be easily monitored by ^31^P NMR (Figure 4) by sampling at each intermediate stage; signals for the pair of diastereomers are evident throughout the series.

The palladium-mediated base insertion reaction was carried out under microwave conditions as previously described to give **25** in 34% yield. Concomitant hydrogenation of the cyclopentyl alkene and removal of the benzyl group by hydrogenolysis under an atmosphere of hydrogen in the presence of 5% Pd/C afforded the target prodrug **26** in 90% yield.

### CycloSal Prodrugs

We next decided to examine the CycloSal prodrug moiety, developed by Meier and co-workers, although CycloSal phosphonates have been found to be somewhat more labile than similar phosphates.^33^ The late-stage hydrogenation approach seemed unlikely to be compatible with the CycloSal function so we adopted a different approach to this compound, involving the use of a *tert*-butyl ester which could be cleaved under acidic conditions. Notably, Meier described the trifluoroacetic acid (TFA) deprotection of monomethoxytrityl (MMTr)-protected PMEA CycloSal derivatives, indicating that the CycloSal phosphonate moiety can survive acidic conditions late in the sequence.^33^

**Scheme 5:**
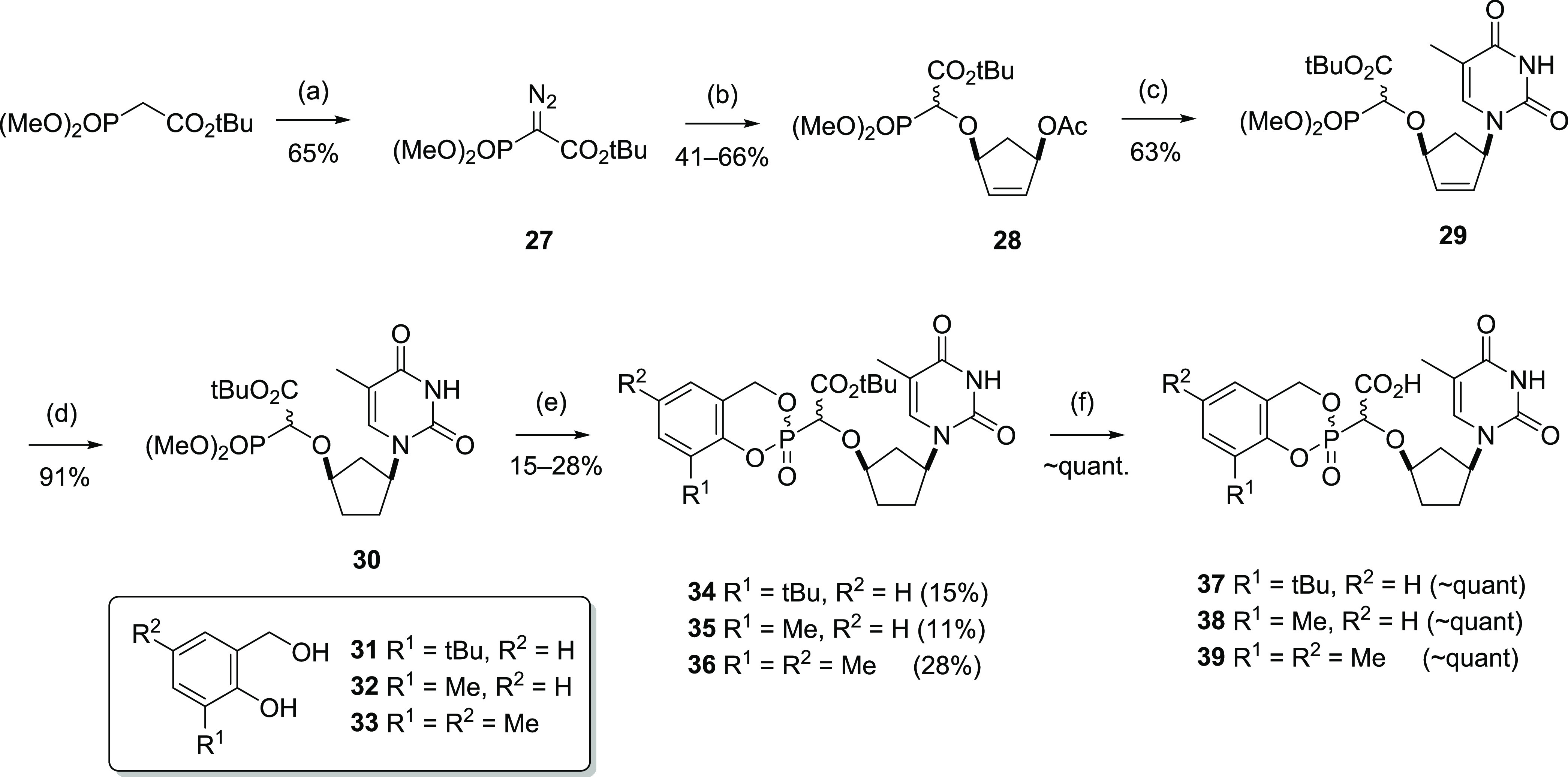
Synthesis of CycloSal Prodrugs Conditions: (a) ABSA, 1,8-diazabicyclo[5.4.0]undec-7-ene (DBU), dimethyl sulfoxide (DMSO), rt, 30 min; (b) **12**, Cu(OTf)_2_, C_6_H_6_, 80 °C, 30 min (41%), or **12**, Rh_2_(O_2_CC(CH_3_)_3_)_4_, CH_2_Cl_2_, 40 °C (66%); (c) thymine, aq. Na_2_CO_3_, Pd(dba)_2_/dppb, DMF, 60 °C, 30 min; (d) H_2_, 5% Pd/C 1 atm, MeOH, rt, 18 h; (e, i) TMSBr, lutidine, MeCN, 50 °C microwave, 30 min; (ii) (COCl)_2_, cat. DMF, CH_2_Cl_2_, rt 3 h; (iii) **31**–**33**, Et_3_N, CH_2_Cl_2_, 0 °C to rt, 18 h; (f) TFA, CH_2_Cl_2_, 0 °C to rt, 18 h.

Thus, the synthesis started from commercially available *tert*-butyl dimethylphosphonoacetate, which was converted to the corresponding diazo compound **27** and reacted with the acetoxy alcohol **12** to afford the O–H insertion product **28** (Scheme 5). While this O–H insertion reaction could be catalyzed by Rh_2_(OAc)_4_ or Rh_2_(piv)_4_, both of these catalysts also led to formation of a substantial amount of an unidentified side-product, which hampered purification, but the desired product was isolated in 66% yield. Use of Cu(OTf)_2_ instead of rhodium catalysts afforded the desired product without forming the undesired side-product, although the eventual yield was slightly lower (41%). Purification of the product from the copper-catalyzed reaction was much more straightforward on a multi-gram scale, so this was the preferred method of preparation of **28**. With the intermediate **28** in hand, the base insertion reaction with thymine was carried out, to give **29**. In this case the reaction was most conveniently performed in DMF under thermal conditions, rather than under microwave irradiation, typically affording 50–60% yield following evaporation of the solvents and purification by chromatography. Product **29** could also be isolated by extractive workup, although sometimes the product was isolated with a diastereomer ratio far from 1:1; in these cases, the diastereomers could be equilibrated using a catalytic amount of triethylamine. Hydrogenation of **29** to afford the key intermediate **30** was straightforward using a balloon of hydrogen and 5% palladium on carbon. The CycloSal group was installed in a similar manner to that described by Meier et al.;^33^ the dimethyl phosphonate group was deprotected using TMSBr, in the presence of 2,6-lutidine to prevent cleavage of the *tert*-butyl ester, and the resulting bis-TMS intermediate was treated with oxalyl chloride and a catalytic amount of DMF to give the dichloridate, which was then reacted with the relevant salicyl alcohol **31**–**33** to give the penultimate intermediates **34**–**36** in low to moderate yields and with variable diastereomer ratios, which could be observed in the NMR spectra of the products. Three salicyl alcohols were used, 3-*tert*-butyl **31**, 3-methyl **32**, and 3,5-dimethyl **35**, while the attempted preparation of the 5-chloro analogue was unsuccessful. Treatment of **34**–**36** with TFA in CH_2_Cl_2_, starting in an ice bath and allowed to warm slowly overnight, afforded the free acid compounds **37**–**39** in essentially quantitative yields after evaporation of the volatiles and drying in vacuo.

### Bis(Acyloxybenzyl)phosphonate Prodrug

**Scheme 6:**
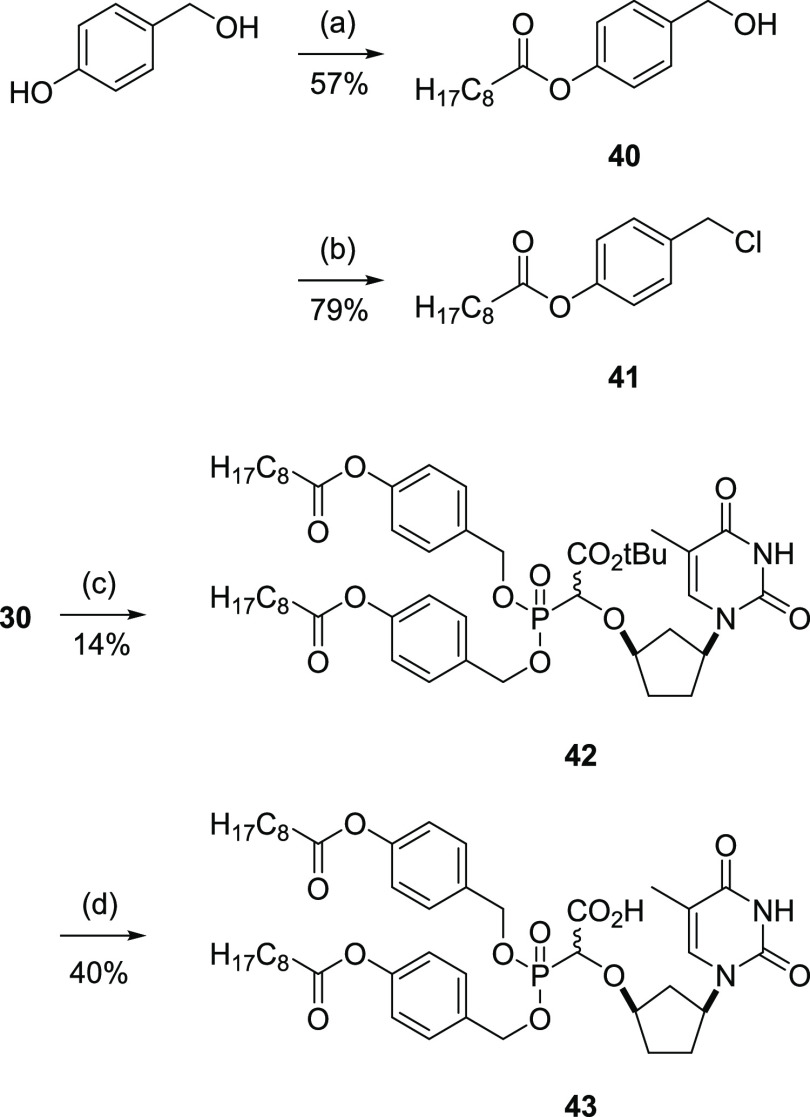
Synthesis of the Bis(acyloxybenzyl) Phosphonate **43** Conditions: (a) nonanoyl chloride, Et_3_N, CH_2_Cl_2_, rt, 18 h; (b) SOCl_2_, CH_2_Cl_2_, rt, 1.5 h; (c) **41**, NaI, MeCN, 80 °C, 48 h; (d) TFA, CH_2_Cl_2_, 0 °C, 2 h.

The last prodrug derivative examined in this study was the 4-nonanoyloxybenzyl phosphonate **43** (Scheme 6). As discussed above, the cleavage of the acyloxybenzyl group is initiated by enzymatic hydrolysis of the acyl groups, which leads to spontaneous expulsion of quinone methides to leave the free phosphonate. This was attractive to us since the initial hydrolysis would occur remotely from the α-CNP function, in principle overcoming concerns that the α-CNP group itself may be incompatible with cellular enzymes. The bis(nonanoyloxybenzyl) α-CNP derivative **42** was prepared in a similar way to the bis-POM derivative **2** using the one-pot procedure, from the *tert*-butyl dimethyl phosphonate **30** by reaction with 4-nonanoyloxybenzyl chloride **41** (prepared from 4-hydroxybenzyl alcohol via acylation to **40** followed by conversion to the chloride using thionyl chloride) and sodium iodide in acetonitrile. The carboxylic acid was revealed by TFA hydrolysis to give the desired product **43** in 40% yield; the yield of **43** from the hydrolysis reaction was a balance between incomplete reaction and hydrolysis of the phosphonate function; difficulties in purification meant that the isolated product **43** was approximately 80% pure, judged by the NMR spectra.

Throughout this investigation, the lipophilic phosphonate prodrugs are generally isolated as mixtures of diastereomers; in the NMR spectra, the signals for the PCH (δ_H_ ∼ 4.5 doublet, *J* ∼ 20 Hz, and δ_C_ ∼ 74 doublet *J* ∼ 150 Hz) are particularly characteristic and enabled the assignment of the diastereomeric ratios.

### Biochemical Evaluation of Free Acid Prodrugs

Unfortunately, almost all of the prodrug derivatives were inactive against HIV-1 in CEM cell cultures, although **3** displayed limited activity (inhibitory concentration-50 (IC_50_) 59 ± 17 μM) against HIV-1 in the cellular assays.

The reason for such poor, if any, activity of the prodrugs with the free acids may be explained by (a) lack of sufficient lipophilicity to facilitate significant transportation of the molecule into the cell in the presence of the free carboxylic acid or (b) release of the α-CNP **1** in the cell is hindered by the aforementioned acid function. In fact, to date, no activity of nucleoside phosphonates in which an additional negative charge is present near the phosphonate moiety has been documented, to our knowledge. However, more data is required to fully elucidate the mechanistic basis for the lack of activity. Clearly, with the methyl ester in place the prodrugs were similarly inactive which presumably indicates that the enzymatic release of the free α-CNP is hindered by the presence of the ester moiety relative to the standard phosphononucleoside POC and POM derivatives. Interestingly, in our earlier investigations, the methyl esters of the α-CNPs, both the saturated and unsaturated T-α-CNPs, were inactive against HIV-1 RT,^7^ indicating that enzymatic ester hydrolysis to release the free acid might not proceed in vivo, and is a prerequisite for any eventual antiviral activity in drug-exposed cells. These results indicate the need for a separate prodrug for the carboxy group in conjunction with the phosphonate protection or a tether to link the phosphonate and carboxy groups to facilitate cell penetration.

## Conclusions

In an effort to evaluate the potency of α-CNPs against HIV-1 in vivo (intact virus-infected cells), we synthesized a number of prodrugs, demonstrating methodologies for the attachment of a range of lipophilic groups to the phosphonate, with and without protection at the adjacent carboxylic acid, building on earlier work by a number of teams using simple phosphonates, without the complication of an adjacent carboxylic acid moiety. Key to this was the use of the α-diazophosphonates bearing either a benzyl or *tert*-butyl ester enabling selective deprotection at this group late in the synthetic sequence. The bis-POM methyl ester prodrugs **2** and **8** were inactive against HIV-1. Among the “free acid” prodrugs **3**, **19**–**21**, **26**, **37**–**39**, and **43**, only the bis-POM derivative **3** displayed a certain degree of poor activity against HIV-1 in cellular assays.

## Experimental Section

### General Procedures

Solvents were distilled prior to use as follows: dichloromethane was distilled from phosphorus pentoxide and/or calcium hydride; ethyl acetate was distilled from potassium carbonate. Benzene was dried before use with activated 4 Å molecular sieves. For O–H and base insertion reactions, solvents were degassed by purging with nitrogen. Organic phases were dried using anhydrous magnesium sulfate. All commercial reagents were used without further purification. Microwave reactions were carried out in closed vessels using a CEM Discover SP in conjunction with Synergy software; reaction temperatures were measured by IR sensor. ^1^H, ^13^C, and ^31^P spectra were recorded at 20 °C on 300, 400, or 600 MHz spectrometers. ^1^H and ^13^C chemical shifts are given in ppm (δ), referenced to solvent signals. ^31^P chemical shifts are referenced to H_3_PO_4_ (external standard), and ^19^F chemical shifts are referenced to C_6_F_6_. Coupling constants (*J*) are given in hertz (Hz). In some cases, in ^13^C NMR spectra of diastereomer mixtures, several resonances with phosphorus coupling were very closely spaced and have been designated as multiplets (m) with a chemical shift range. High-resolution mass spectra (HRMS) were recorded on a time-of-flight spectrometer in electrospray ionization (ESI) mode. Column chromatography was performed using silica gel 60. Thin-layer chromatography (TLC) was carried out on precoated silica gel plates (60 PF254). Visualization was achieved by UV (254 nm) detection and/or staining with vanillin, permanganate, or ceric ammonium molybdate. *cis*-4-Hydroxy-2-cyclopentenyl acetate **12**,^3,58−60^ ABSA,^61^ HDP-OH,^62^ POM-I,^63^ POC-Cl,^64^ and POC-I^65^ and the salicyl aclohols **31**–**33**^66−68^ were prepared by literature methods. Trimethyl phoshonoacetate and *tert*-butyl dimethylphosphonoacetate were purchased from TCI; 4-hydroxybenzyl alcohol was purchased from Merck.

### Cellular HIV-RT Assays

The procedure to determine the anti-HIV-1(IIIB) activity in human lymphocytic CEM cell cultures has been described before.^19,33^ Briefly, 200 μL of CEM cell cultures (250,000–300,000 cells/mL) was seeded in the wells of 96-well microtiter trays and exposed to 100 CCID_50_ (cell culture-infective dose, 50) HIV-1 (strain IIIB)/mL. The wells contain a serial dilution (5-fold) of the test compounds at 100 μM as the highest compound concentration. After 4 days of incubation at 37 °C, HIV-1-induced syncytia formation in the absence (control) or presence (test) of the test compounds was microscopically examined. The inhibitory concentration-50 (IC_50_) was defined as the test compound concentration required to inhibit virus-induced syncitium formation by 50%.

### *cis*-1-(4-((Methoxycarbonyl)bis(pivaloyloxymethyl)phosphonomethoxy)cyclopentan-1-yl)thymine **2** (Bis-POM T-α-CNP Methyl Ester)

A solution of *cis*-1-(4-((methoxycarbonyl)dimethylphosphonomethoxy)cyclopentan-1-yl)thymine **5** (0.23 g, 0.6 mmol), TMSBr (306 μL, 0.35 g, 2.3 mmol) and acetonitrile (3 mL) in a microwave vial was heated at 50 °C under microwave irradiation at 50 W for 10 min. Thereafter, the reaction was quenched with MeOH/H_2_O (95:5) and the mixture concentrated under reduced pressure. The resultant amber oil was dissolved in THF (15 mL), Hünig’s base (556 μL, 0.42 g, 3.2 mmol) was added followed by POM iodide (0.45 g, 1.85 mmol), and the reaction mixture was stirred overnight. After removal of suspended solids by filtration and concentration of the filtrate under reduced pressure, the residue was purified by flash chromatography (5% MeOH/CH_2_Cl_2_) to afford the desired product **2** as an oil (0.32 g, 94%) as an essentially equimolar mixture of diastereomers: ^1^H NMR (300 MHz, CDCl_3_): δ 8.71 (bs, 1H) 7.78 (bs, 0.5H), 7.63 (bs, 0.5H), 5.82–5.61 (m, 4H), 5.33–5.15 (m, 1H), 4.49 (d, 0.5H, *J* = 20.1), 4.45 (d, 0.5H, *J* = 20.1), 4.31–4.16 (m, 1H), 3.82 (s, 3H), 2.49–2.31 (m, 1H), 2.28–1.76 (m, 7H), 1.69–1.50 (m, 1H), 1.28–1.20 (m, 18H). ^13^C{^1^H} NMR (75.5 MHz, CDCl_3_): δ 176.8, 176.6, 166.9 (d, *J* = 3.1), 166.8 (d, *J* = 2.4), 163.73, 163.71, 151.31, 151.27, 138.1, 137.9, 111.8, 111.7, 82.8 (d, *J* = 8.1), 82.4 (d, *J* = 10.8), 82.2–82.1 (m), 73.9 (d, *J* = 162.6), 73.7 (d, *J* = 164.3), 53.2, 53.1, 53.0, 38.7, 38.4, 38.2, 31.1, 30.8, 30.0, 29.9, 26.8, 12.3, 12.2, ^31^P{^1^H} NMR (121.5 MHz, CDCl_3_): δ 13.75, 13.69. HRMS (ES+) *m*/*z*: [M + H]^+^ calcd for C_25_H_40_N_2_O_12_P 591.2319; found 591.2319.

### *cis*-1-(4-((Methoxycarbonyl)bis(pivaloyloxymethyl)phosphonomethoxy)cyclopentan-1-yl)-5-fluorouracil **8** (Bis-POM 5-F-U-α-CNP Methyl Ester)

This was prepared following the procedure described for **2**, starting from phosphonate ester **7** (81 mg, 0.205 mmol) and TMSBr (126 mg, 106 μL, 0.820 mmol) in acetonitrile (3 mL). The solution was heated at 50 °C under microwave irradiation at 50 W for 10 min. Water (0.2 mL) and methanol (0.2 mL) were added, and the mixture was stirred for 20 min at room temperature. The reaction mixture was concentrated, and the residue was dissolved in dry acetonitrile (13 mL). A solution of Hünig’s base (146 mg, 196 μL, 1.127 mmol) in acetonitrile (2.6 mL) and a solution of POM iodide (154 mg, 0.636 mmol) in acetonitrile (2.6 mL) were added, and the mixture was stirred overnight and then concentrated in vacuo. Purification by flash chromatography (SiO_2_, 2% MeOH/CH_2_Cl_2_) yielded **8** as a colorless gum (38 mg, 31%). ^1^H NMR (300 MHz, CDCl_3_): δ 9.21 (br s, 1H), 8.18 (d, 0.5H, *J* = 6.6), 8.05 (d, 0.5H, *J* = 6.6), 5.85–5.62 (m, 4H), 5.32–5.22 (m, 1H), 4.50 (d, 0.5H, *J* = 20.6), 4.46 (d, 0.5H, *J* = 19.2), 4.31–4.29 (m, 0.5H), 4.20–4.19 (m, 0.5H), 3.82, 3.81 (2× s, 3H), 2.43–1.49 (m, 6H), 1.24, 1.23 (2× s, 18H). ^13^C{^1^H} NMR (75.5 MHz, CDCl_3_): δ 176.89, 176.86, 176.70, 176.67, 166.9 (d, *J* = 3.2), 166.6 (d, *J* = 2.3), 156.9 (d, *J* = 27.0), 156.6 (d, *J* = 26.7), 149.95, 149.92, 140.93 (d, *J* = 236.6), 140.86 (d, *J* = 236.3), 126.84 (d, *J* = 33.6), 126.78 (d, *J* = 33.7), 82.8 (d, *J* = 7.7), 82.4–82.0 (m), 73.9 (d, *J* = 162.8), 73.5 (d, *J* = 164.8), 54.1, 54.0, 53.09, 53.05, 38.75, 38.70, 38.5, 31.1, 30.9, 30.7, 30.0, 26.8. ^31^P{^1^H} NMR (121.5 MHz, CDCl_3_): δ 13.80, 13.60. ^19^F{^1^H} NMR (282.4 MHz, CDCl_3_): δ −163.70, −163.89. HRMS (ES+) *m*/*z*: [M + H]^+^ calcd for C_24_H_37_FN_2_O_12_P 595.2068; found 595.2068.

### Benzyl (Dimethylphosphono)acetate **10**(69)

A suspension of trimethyl phosphonoacetate **9** (20 g, 0.109 mol) and potassium carbonate (20 g, 0.145 mol) in water (500 mL) was stirred at room temperature overnight. The reaction mixture was concentrated for 3 h at 45 °C under vacuum and the residue taken up in acetonitrile (200 mL). Neat benzyl bromide (16.0 mL, 0.135 mol) was added, and the reaction mixture was refluxed for 48 h, cooled to room temperature, and concentrated to dryness. The residue was purified by chromatography (SiO_2_, 5% MeOH/CH_2_Cl_2_) to give the title compound **10** as a colorless oil (12.91 g, 46%). ^1^H NMR (300 MHz, CDCl_3_): δ 7.40–7.29 (m, 5H), 5.18 (s, 2H), 3.76 (d, 6H, *J* = 11.2), 3.02 (d, 2H, *J* = 21.5). ^13^C{^1^H} NMR (75.5 MHz, CDCl_3_): δ 165.4 (d, *J* = 6.0), 135.2, 128.5, 128.4, 128.3, 67.4, 53.1 (d, *J* = 6.4), 33.4 (d, *J* = 135.1). ^31^P{^1^H} NMR (121.5 MHz, CDCl_3_): δ 22.18.

### Benzyl (Dimethylphosphono)diazoacetate **11**(53)

Triethylamine (1.1 mL, 0.79 g, 7.78 mmol) was added to a solution of benzyl (dimethylphosphono)acetate **10** (2.01 g, 7.78 mmol) and dodecylbenzenesulfonyl azide (2.73 g, 7.78 mmol) in acetonitrile (25 mL) and stirred at room temperature overnight and then at 40 °C for 2h. Silica gel (ca. 10 g) was added, and the volatiles were removed under reduced pressure. The residue was purified by flash chromatography (60% EtOAc/hexane) to afford the desired product **11** as a yellow oil (1.3 g, 59%). ^1^H NMR (300 MHz, CDCl_3_): δ 7.39–7.33 (m, 5H), 5.24 (s, 2H), 3.80 (d, 6H, *J* = 12.0). ^13^C {^1^H}NMR (75.5 MHz, CDCl_3_): δ 163.0 (d, *J* = 12.2), 135.2, 128.5, 128.4, 128.1, 67.2, 53.8 (d, *J* = 5.7) (signal for CN_2_ not observed). ^31^P{^1^H} NMR (121.5 MHz, CDCl_3_): δ 13.13.

### *cis*-1-((Benzyloxycarbonyl)dimethylphosphonomethoxy)-4-acetoxycyclopent-2-ene **13**

A solution of 4-hydroxycyclopent-2-en-1-yl acetate **12** (0.57 g, 4.0 mmol) and benzyl (dimethoxyphosphono)diazoacetate **11** (1.25 g, 4.5 mmol) in CH_2_Cl_2_ (30 mL) was purged with nitrogen for 10 min; then, 4 Å molecular sieve (ca. 4 g) was added, and the mixture allowed to stand for 6 h. Rhodium pivalate (20 mg, 0.033 mmol, 0.75 mol %) was added, and the reaction mixture was stirred gently and heated to reflux overnight. The mixture was cooled, filtered, and evaporated to afford a green residue, which was purified by chromatography (SiO_2_, 60–80% EtOAc/hexanes) to give the desired product **13** as a colorless viscous oil as an essentially equimolar mixture of diastereomers (1.09 g, 68%). ^1^H NMR (300 MHz, CDCl_3_): δ 7.41–7.33 (m, 5H), 6.11–5.98 (m, 2H), 5.49–5.40 (m, 1H), 5.34–5.18 (m, 2H), 4.73–4.69 (m, 0.5H), 4.63–4.59 (m, 0.5H), 4.53 (d, 0.5H, *J* = 19.9), 4.49 (d, 0.5H, *J* = 20.1), 3.82–3.72 (m, 6H,), 2.78–2.67 (m, 1H), 2.01 (s, 1.5H), 0.99 (s, 1.5H), 1.82 (dt, 0.5H, *J* = 15.0, 3.8), 1.72 (dt, 0.5H, *J* = 14.6, 4.0). ^13^C{^1^H} NMR (75.5 MHz, CDCl_3_): δ 170.6, 170.5, 167.6 (d, *J* = 2.7), 167.4 (d, *J* = 2.5), 135.2, 135.1, 134.98, 134.95, 134.5, 134.3, 128.53, 128.52, 128.47, 128.45, 84.1 (d, *J* = 12.0), 84.0 (d, *J* = 11.6), 76.2, 76.1, 74.1 (d, *J* = 159.8), 73.4 (d, *J* = 159.4), 67.62, 67.56, 54.2–54.1 (m), 36.70, 36.65, 20.97, 20.95. ^31^P{^1^H} NMR (121.5 MHz, CDCl_3_): δ 16.72, 16.43. HRMS (ES+) *m*/*z*: [M + H]^+^ calcd for C_18_H_24_O_8_P 399.1209; found 399.1203.

### *cis*-1-(4-((Benzyloxycarbonyl)dimethylphosphonomethoxy)cyclopent-2-en-1-yl)thymine **14**

A microwave vial containing a degassed suspension of thymine (190 mg, 1.51 mmol) and sodium carbonate (160 mg, 1.51 mmol) in water (2 mL) and acetonitrile (2 mL) was heated under microwave conditions (50 °C, 200 W) for 30 min. A degassed solution of **13** (408 mg, 1.06 mmol) in acetonitrile (3 mL), bis(dibenzylideneacetone)palladium(0) Pd(dba)_2_ (30 mg, 5 mol %), and 1,4-bis(diphenylphosphino)butane (dppb) (42 mg, 9 mol %) was added to the vial. The resulting mixture was irradiated (50 °C, 200 W) for 30 min, whereupon a second portion of Pd(dba)_2_ (30 mg) and dppb (42 mg) was added followed by irradiation (50 °C, 200 W) for a further 30 min. The reaction mixture was cooled to room temperature, gravity filtered, and concentrated under vacuum to give a purple residue, which was purified by chromatography (SiO_2_, 3% MeOH/CH_2_Cl_2_) to afford compound **14** (127 mg, 27%) as a colorless foam, which was a roughly equal mixture of diastereomers. ^1^H NMR (300 MHz, CDCl_3_): δ 9.48 (bs, 1H) 7.40–7.28 (m, 5H), 7.28–7.25 (m, 0.5H), 7.22–7.18 (m, 0.5H), 6.26 (dt, 0.5H, *J* = 5.2, 1.9), 6.18 (dt, 0.5H, *J* = 5.5, 1.9), 5.90, (dd, 0.5H, *J* = 5.5, 1.8), 5.86 (dd, 0.5H, *J* = 5.5, 2.2), 5.67–5.57 (m, 1H), 5.34–5.14 (m, 2H), 4.65–4.59 (0.5H, m), 4.59–4.46 (m, 1.5H, containing 4.56 d, 0.5H, *J* = 19.2 and 4.54 d, 0.5H, *J* = 19.8), 3.82–3.72 (m, 6H), 2.82–2.69 (m, 1H), 1.88 (s, 3H), 1.78–1.67 (m, 1H). ^13^C{^1^H} NMR (75.5 MHz, CDCl_3_): δ 167.2 (d, *J* = 2.2), 166.9 (d, *J* = 2.4), 164.0, 151.1, 151.0, 137.1, 137.0, 135.8, 135.3, 134.8, 134.70, 134.67, 134.60, 128.6, 128.51, 128.47, 111.5, 111.4, 84.9–84.7 (m), 75.1 (d, *J* = 159.3), 75.0 (d, *J* = 159.4), 67.77, 67.75, 57.7, 57.6, 54.1–53.7 (m), 37.0, 36.8, 12.1. ^31^P{^1^H} NMR (121.5 MHz, CDCl_3_) δ: 16.38, 16.21. HRMS (ES+) *m*/*z*: [M + H]^+^ calcd for C_21_H_26_N_2_O_8_P 465.1427; found 465.1427.

### *cis*-1-(4-((Benzyloxycarbonyl)bis(pivaloyloxymethyl)phosphonomethoxy)cyclopent-2-en-1-yl)thymine **15** (Bis-POM Unsaturated T-α-CNP Benzyl Ester)

*Method A*: Bromotrimethylsilane (56 μL, 0.44 mmol) and lutidine (50 μL, 0.44 mmol) were sequentially added via a syringe to a solution of *cis*-1-(4-((benzyloxycarbonyl)dimethylphosphonomethoxy)cyclopent-2-en-1-yl)thymine **14** (50 mg, 0.11 mmol) (diastereomeric ratio 46:54) in acetonitrile (2 mL). The solution was heated at 50 °C for 10 min under microwave irradiation at 50 W. Upon cooling, water (50 μL) and methanol (950 μL) were added and the mixture was stirred for 10 min at room temperature. The reaction mixture was concentrated in vacuo, and the residue was dissolved in dry THF (5 mL). A solution of DIPEA (100 μL, 1.05 mmol) in THF (1 mL) and a solution of POM iodide (145 mg, 0.60 mmol) in THF (1 mL) were added, and the mixture was stirred overnight at room temperature. The reaction mixture was concentrated in vacuo and purified by flash chromatography (SiO_2_, 5% methanol in dichloromethane) to give the bispivaloyloxymethyl product as a yellow gum containing some impurities. This gum was further purified by dissolving in dichloromethane (2 mL) and washing with HCl solution (1 M, 2 × 5 mL) and brine (2 × 5 mL). The organic layer was dried (MgSO_4_), filtered, and concentrated to give the desired product **15** as a yellow gum (10 mg, 14%) as a roughly equal mixture of diastereomers.

*Method B*: one-pot procedure: A mixture of **14** (50 mg, 0.108 mmol), POM-Cl (35 μL, 36 mg, 0.237 mmol), and NaI (32 mg, 0.216 mmol) in acetonitrile (1 mL) was sealed in a crimp-cap vial and stirred at 80 °C for 48 h. Two such reaction mixtures were combined in CH_2_Cl_2_ (25 mL), and SiO_2_ (ca. 3 mL) was added. After removal of the solvents, the residue was purified by chromatography (SiO_2_, 80% EtOAc/hexanes) to afford 66 mg (46%) of **15** as a colorless film. ^1^H NMR (300 MHz, CDCl_3_): δ 8.29 (bs, 1H), 7.41–7.30 (m, 5H), 7.30–7.27 (m, 0.5H), 7.24–7.20 (m, 0.5H), 6.29 (dt, 0.5H, *J* = 5.6, 1.9), 6.19 (d, 0.5H, *J* = 5.5, 1.9), 5.94–5.86 (m, 1H), 5.74–5.54 (m, 5H), 5.35–5.10 (m, 2H), 4.72–4.61 (m, 1H), 4.57 (d, 0.5H, *J* = 19.3), 4.56 (d, 0.5H, *J* = 20.1), 2.85–2.67 (m, 1H), 1.91, 1.90 (2× s, 3H), 1.83–1.68 (m, 1H), 1.21, 1.205, 1.203, 1.19 (4× s, 18H). ^13^C{^1^H} NMR (75 MHz, CDCl_3_): δ 176.73, 176.66, 176.56, 176.53, 166.3 (d, *J* = 3), 166.1 (d, *J* = 3), 164.0, 151.08, 151.05, 137.2, 137.1, 135.5, 134.8, 134.62, 134.61, 134.57, 128.7, 128.59, 128.57, 128.5, 128.4, 111.6, 85.2 (d, *J* = 9), 85.0 (d, *J* = 11), 82.2–82.0 (m), 75.2 (d, *J* = 163), 74.9 (d, *J* = 163), 67.9, 57.7, 57.6, 38.6, 36.9, 36.8, 26.7, 12.2. ^31^P{^1^H} NMR (121.5 MHz, CDCl_3_) δ: 13.38, 13.16 (unidentified minor peaks (ca. 5%) at 12.44 & 12.19). HRMS (ES+) *m*/*z*: [M + H]^+^ calcd for C_31_H_42_N_2_O_12_P 665.2470; found 665.2488.

### *cis*-1-(4-((Carboxy)bis(pivaloyloxymethyl)phosphonomethoxy)cyclopentan-1-yl)thymine **3** (Bis-POM T-α-CNP Free Acid)

*cis*-1-(4-((Benzyloxycarbonyl)bis(pivaloyloxymethyl)phosphonomethoxy)cyclopentan-1-yl)thymine **15** (46 mg, 0.069 mmol) was dissolved in methanol (5 mL) and flushed with nitrogen. Palladium on carbon 5% (5 mg) was added, and the suspension was stirred under a hydrogen-filled balloon at room temperature for 24 h. The reaction mixture was filtered using a syringe filter and concentrated to give product **3** as a colorless film (39 mg, ∼quantitative) as a roughly equal mixture of diastereomers. ^1^H NMR (300 MHz, CDCl_3_): δ: 9.14 (bs, 1H), 7.87 (s, 0.5H), 7.67 (s, 0.5H), 5.87–5.62 (m, 4H) 5.30–5.11 (m, 1H), 4.50 (d, 0.5H, *J* = 20.8), 4.48 (d, 0.5H, *J* = 19.9), 4.35–4.21 (m, 1H), 2.47–2.30 (m, 1H), 2.26–2.02 (m, 2H), 1.98–1.75 (m, 5H, contains 1.95 s and 1.94 s), 1.70–1.51 (m, 1H), 1.30–1.15 (m, 18H). ^13^C{^1^H} NMR (75 MHz, CDCl_3_): δ 176.85, 176.83, 176.76, 168.9 (d, *J* = 4), 168.6 (d, *J* = 4), 164.7, 164.6, 151.4, 139.1, 138.7, 111.6, 111.5, 82.7–82.4 (m), 73.8 (d, *J* = 163), 73.7 (d, *J* = 163), 53.5, 53.4, 38.7, 38.6, 38.2, 31.0, 30.9, 30.1, 29.9, 26.8, 12.12, 12.11. ^31^P (121 MHz, CDCl_3_): δ 14.49, 14.41 (unidentified minor peaks (ca. 5%) at 12.73 & 12.69). HRMS (ES+) *m*/*z*: [M + H]^+^ calcd for C_24_H_38_N_2_O_12_P 577.2162; found 577.2164.

### *cis*-1-(4-((Benzyloxycarbonyl)bis(isopropoxycarbonyloxymethyl)phosphonomethoxy)cyclopent-2-en-1-yl)thymine **16** (Bis-POC Unsaturated T-α-CNP Benzyl Ester)

*Method A*: Bromotrimethylsilane (120 μL, 0.90 mmol) and lutidine (100 μL, 0.86 mmol) were sequentially added via a syringe to a solution of **14** (100 mg, 0.22 mmol) in acetonitrile (2 mL). The solution was heated at 50 °C for 10 min under microwave irradiation at 50 W. Upon cooling, water (50 μL) and methanol (950 μL) were added and the mixture was stirred for 10 min at room temperature. The reaction mixture was concentrated in vacuo, and the residue was dissolved in dry THF (5 mL). A solution of DIPEA (100 μL, 0.56 mmol) in THF (1 mL) and a solution of POC-iodide (200 mg, 0.82 mmol) in THF (1 mL) were added, and the mixture was stirred overnight at room temperature. The reaction mixture was concentrated in vacuo and purified by flash chromatography (SiO_2_, 5% MeOH/CH_2_Cl_2_) to give the bis-POC product **16** as a yellow gum (12 mg, 8%).

*Method B*: one-pot procedure: A mixture of **14** (30 mg, 0.066 mmol), sodium iodide (20 mg, 0.13 mmol), and POC-Cl (25 mg, 0.16 mmol) in MeCN (1 mL) was placed in a microwave vial. The vial was sealed, and the reaction mixture was heated at 80 °C for 48 h with stirring. After cooling, ether (5 mL) was added and the solution washed with water (2 × 5 mL), dried (MgSO_4_), filtered, and evaporated. The residue was purified by chromatography (SiO_2_, 5% MeOH/CH_2_Cl_2_) to afford the desired product **16** (16 mg, 36%). ^1^H NMR (300 MHz, CDCl_3_): δ 8.39 (br s, 1H), 7.41–7.32 (m, 5H), 7.30–7.27 (m, 0.5H), 7.23–7.21 (m, 0.5H), 6.29 (dt, 0.5H, *J* = 5.5, 1.9), 6.20 (dt, 0.5 H, *J* = 5.5, 1.9), 5.96–5.83 (m, 1H), 5.74–5.53 (m, 5H), 5.35–5.15 (m, 2H), 4.99–4.81 (m, 2H), 4.71–4.53 (m, 2H, contains 4.60 d, *J* = 19.6 and 4.59 d, *J* = 20.3), 2.86–2.65 (m, 1H), 1.90 (2 unresolved s, 3H), 1.86–1.74 (m, 1H), 1.35–1.23 (m, 12H). ^13^C{^1^H} NMR (75 MHz, CDCl_3_): δ 166.2 (d, *J* = 3.1), 166.0 (d, *J* = 3.5), 163.9, 153.0, 152.95, 152.92, 152.87, 151.1, 151.0, 137.2, 137.1, 135.6, 135.5, 134.8, 134.64, 134.60, 128.6, 128.58, 128.56, 128.50, 128.45, 111.6, 85.4 (d, *J* = 10.0), 85.2 (d, *J* = 11.4), 84.9–84.6 (m), 75.3 (d, *J* = 163.5), 75.0 (d, *J* = 163.6), 73.4 br, 68.0, 57.7, 57.6, 36.84, 36.75, 21.5, 12.1. ^31^P{^1^H} NMR (121 MHz, CDCl_3_): δ 13.68, 13.38 (unidentified minor peaks (ca. 10%) at 12.72 and 12.39). HRMS (ES+) *m*/*z*: [M + H]^+^ calcd for C_29_H_38_N_2_O_14_P 669.2061; found 669.2037.

### *cis*-1-(4-((Carboxy)bis(isopropoxycarbonyloxymethyl)phosphonomethoxy)cyclopentan-1-yl)thymine **19** (Bis-POC T-α-CNP Free Acid)

A mixture of **16** (59 mg, 0.088 mmol) and palladium on carbon (5 mg, 10%) in methanol (5 mL) was stirred under a balloon of hydrogen at atmospheric pressure for 18 h at room temperature. After this time, analysis of a small sample by ^1^H NMR spectroscopy revealed reduction of the double bond but the continued presence of the benzyl ester. Fresh catalyst (5 mg) was added, and the mixture was stirred for an additional 6 h under a hydrogen balloon, after which time the reaction was found to be complete. The mixture was filtered through a syringe filter and concentrated to give **19** as a colorless film (44 mg, 86%). ^1^H NMR (300 MHz, CDCl_3_): δ 8.82, 8.79 (2× bs, 1H) 7.83, (s, 0.5H) 7.63 (s, 0.5H), 5.87–5.66 (m, 4H), 5.30–5.11 (m, 1H), 4.89–4.85 (m, 2H), 4.53 (d, 0.5H, *J* = 20.6), 4.50 (d, 0.5H, *J* = 20.1), 4.33–4.21 (m, 1H), 2.46–2.31 (m, 1H), 2.25–2.03 (m, 2H), 1.99–1.78 (m, 5H, contains singlets at 1.95 and 1.93), 1.68–1.50 (m, 1H), 1.31 (d, 12H, *J* = 6.9). ^13^C{^1^H} NMR (75 MHz, CDCl_3_): δ 168.7 br, 168.4 br, 164.6, 153.0, 151.4, 139.1, 138.7, 111.6, 85.1–84.7 (m), 83.1–82.6 (m), 73.8 (d, *J* = 162), 73.7 (d. *J* = 162), 73.6, 73.51, 73.47, 53.4, 38.6, 38.3, 31.0, 30.8, 30.1, 29.9, 21.5, 12.1. ^31^P{^1^H} NMR (121 MHz, CDCl_3_): 14.86, 14.80, (unidentified minor peaks (ca. 10%) at 13.15 & 13.13). HRMS (ES+) *m*/*z*: [M + H]^+^ calcd for C_22_H_34_N_2_O_14_P 581.1748; found 581.1748.

### Diisopropyl 2,2′-(((2-(Benzyloxy)-1-((4-(5-methyl-2,4-dioxo-3,4-dihydropyrimidin-1(2*H*)-yl)cyclopent-2-en-1-yl)oxy)-2-oxoethyl)phosphoryl)bis(azanediyl))(2*S*,2′*S*)-dipropionate **17** (Bisamidate Unsaturated T-α-CNP Benzyl Ester)

Bromotrimethylsilane (60 μL, 0.45 mmol) and lutidine (50 μL, 0.43 mmol) were sequentially added via a syringe to a solution of **14** (50 mg, 0.11 mmol) in acetonitrile (2 mL). The solution was heated at 50 °C for 10 min under microwave irradiation at 50 W. The solution was cooled to room temperature and concentrated to dryness in vacuo, and the residue was dissolved in pyridine (1 mL). Triethylamine (0.20 mL, 1.43 mmol) and l-alanine isopropyl ester hydrochloride (72 mg, 0.43 mmol) were added, and the mixture was heated to 60 °C until a clear solution was obtained (typically 5 min). A freshly prepared solution of Aldrithiol-2 (140 mg, 0.64 mmol) and triphenylphosphine (167 mg, 0.63 mmol) in pyridine (1 mL) was added to the above reaction mixture. The reaction was stirred at 60 °C overnight, cooled to room temperature, and concentrated under vacuum. The residue was dissolved in ethyl acetate (25 mL) and washed with saturated sodium bicarbonate solution (4 × 25 mL), dried (MgSO_4_), filtered, and concentrated in vacuo. Purification of the residue by flash chromatography (SiO_2_, 5% MeOH/CH_2_Cl_2_) afforded the bisamidate **17** as a roughly equal mixture of four diastereomers (25 mg, 34%). ^1^H NMR (300 MHz, CDCl_3_): δ 8.32 (bs, 1H), 7.45–7.20 (m, 6H), 6.35–6.30 (m, 0.25 H), 6.30–6.25 (m, 0.25 H), 6.19–6.13 (m, 0.5H), 5.90–5.80 (m, 1H), 5.65–5.55 (m, 1H), 5.35–5.18 (m, 2H), 5.06–4.88 (m, 2H), 4.69–4.58 (m, 1H), 4.54 (d, 0.25H, *J* = 17.5), 4.51 (d, 0.25H, *J* = 18.0), 4.43 (d, 0.25H, *J* = 18.3), 4.41 (d, 0.25H, *J* = 18.9), 4.13–3.90 (m, 2H), 3.60–3.34 (m, 2H), 2.83–2.66 (m, 1H), 1.92–1.91 (4× s, 3H), 1.89–1.68 (m, 1H), 1.37–1.28 (m, 6H), 1.27–1.18 (m, 12H). ^31^P{^1^H} NMR (121 MHz, CDCl_3_): δ 16.15, 16.09, 15.59, 15.44. No ^13^C NMR data was recorded for this compound. HRMS (ES+) *m*/*z*: [M + H]^+^ calcd for C_31_H_44_N_4_O_10_P 663.2795; found 663.2783.

### 2-(Bis(((*S*)-1-Isopropoxy-1-oxopropan-2-yl)amino)phosphoryl)-2-((4-(5-methyl-2,4-dioxo-3,4-dihydropyrimidin-1(2*H*)-yl)cyclopent-2-en-1-yl)oxy)acetic Acid **20** (Bisamidate T-α-CNP Free Acid)

A mixture of **17** (25 mg, 0.038 mmol) and palladium on carbon (6 mg, 10%) in methanol (2 mL) was stirred under a balloon of hydrogen at atmospheric pressure for 24 h at room temperature. The mixture was filtered on Celite, and the cake was rinsed with methanol. The filtrate was concentrated to give **20** (11 mg, 51%, ∼80% pure by ^1^H NMR). ^1^H NMR (600 MHz, CDCl_3_): δ 8.88 (bs, 1H), 7.87 (s, 0.25H), 7.84 (s, 0.25H), 7.65 (s, 0.25H), 7.61 (s, 0.25H), 5.23–4.91 (m, 4H), 4.42–4.15 (m, 2 H), 4.10–3.66 (m, 5H), 2.44–2.28 (m, 1H), 2.17–2.00 (m, 2H), 1.99–1.71 (m, 5H, includes singlet at 1.94), 1.66–1.50 (m, 2H), 1.46–1.35 (m, 6H), 1.28–1.14 (m, 12H). ^13^C{^1^H} NMR (150 MHz, CDCl_3_): δ 174.3–173.6 (m), 170.9 br, 170.5 br, 169.9, 164.00, 163.98, 151.3, 151.2, 138.8, 138.7, 138.2, 138.0, 111.4, 111.3, 111.2, 82.5–82.0 (m), 77.7–75.0 (m), 70.5–69.2 (m), 53.9–53.6 (m), 50.7, 49.5, 49.33, 49.30, 48.8, 48.7, 48.23, 48.17, 38.6, 38.5, 37.9, 37.8, 31.14, 31.07, 31.0, 30.1, 30.0, 29.6, 21.67, 21.66, 21.64, 21.61, 21.56, 21.40,. 21.38, 21.0, 12.29, 12.27, 12.22, 12.17. ^31^P{^1^H} NMR (121 MHz, CDCl_3_): δ 19.47 br, 19.29 br. HRMS (ES+) *m*/*z*: [M + H]^+^ calcd for C_24_H_40_N_4_O_10_P 575.2482; found 575.2466.

### Isopropyl ((2-(Benzyloxy)-1-((4-(5-methyl-2,4-dioxo-3,4-dihydropyrimidin-1(2*H*)-yl)cyclopent-2-en-1-yl)oxy)-2-oxoethyl)(phenoxy)phosphoryl)-l-alaninate **18** (Phenoxyamidate Unsaturated T-α-CNP Benzyl Ester)

Bromotrimethylsilane (30 μL, 0.22 mmol) and lutidine (25 μL, 0.86 mmol) were sequentially added to a solution of **14** (25 mg, 0.056 mmol) in acetonitrile (2 mL). The solution was heated at 50 °C for 10 min under microwave irradiation at 50 W. The solution was cooled to room temperature and concentrated to dryness in vacuo, and the residue was dissolved in pyridine (1 mL). Triethylamine (0.11 mL, 0.79 mmol), l-alanine isopropyl ester hydrochloride (20 mg, 0.12 mmol), and phenol (25 mg, 0.27 mmol) were added, and the mixture was heated to 60 °C until a clear solution was obtained (typically 5 min). A freshly prepared solution of Aldrithiol-2 (75 mg, 0.34 mmol) and triphenylphosphine (88 mg, 0.34 mmol) in pyridine (1 mL) was added to the above reaction mixture. The reaction was stirred at 60 °C overnight, cooled to room temperature, and concentrated under vacuum. The residue was dissolved in ethyl acetate (25 mL) and washed with saturated sodium bicarbonate solution (4 × 25 mL), dried (MgSO_4_), filtered, and concentrated in vacuo. Purification of the residue by flash chromatography (SiO_2_, 5% methanol in dichloromethane) afforded the phenoxyamidate **18** as a brown gum (8 mg, 22%). ^1^H NMR (300 MHz, CDCl_3_): 8.80–8.68 (m, 1H), 7.43–7.01 (m, 11H), 6.35–6.14 (m, 1H), 5.94–5.79 (m, 1H), 5.69–5.55 (m, 1H), 5.40–5.17 (m, 2H), 5.05–4.88 (m, 1H), 4.77–4.42 (m, 2H), 4.14–3.43 (m, 2H), 2.86–2.67 (m, 1H) 1.95–1.65 (m, 4H), 1.37–1.15 (m, 9H). ^31^P NMR (121 MHz, CDCl_3_) 16.19, 16.13, 15.77, 15.59, 14.98, 14.97, 14.76, 14.72. No ^13^C NMR data was recorded for this compound. HRMS (ES+) *m*/*z*: [M + H]^+^ calcd for C_31_H_37_N_3_O_9_P 626.2267; found 626.2239.

When the reaction above was carried out on larger scale, it was possible to isolate both the bisamidate **17** and the phenoxyamidate **18**. The following amounts of reagents were employed: bromotrimethylsilane (170 μL, 1.29 mmol); lutidine (140 μL, 1.21 mmol); **14** (140 mg, 0.31 mmol); triethylamine (0.60 mL, 4.3 mmol); l-alanine isopropyl ester hydrochloride (108 mg, 0.64 mmol); phenol (150 mg, 1.59 mmol); Aldrithiol-2 (495 mg, 2.25 mmol); and triphenylphosphine (588 mg, 2.24 mmol). Purification of the residue by flash chromatography (SiO_2_, 5% methanol in dichloromethane) afforded the bisamidate **17** (14 mg, 7%) and the phenoxyamidate **18** (40 mg, 21%).

### 2-((((*S*)-1-Isopropoxy-1-oxopropan-2-yl)amino)(phenoxy)phosphoryl)-2-((4-(5-methyl-2,4-dioxo-3,4-dihydropyrimidin-1(2*H*)-yl)cyclopent-2-en-1-yl)oxy)acetic Acid **21** (Phenoxyamidate T-α-CNP Free Acid)

A mixture of **18** (16 mg, 0.256 mmol) and palladium on carbon (5 mg, 10%) in methanol (2 mL) was stirred under a balloon of hydrogen at atmospheric pressure for 24 h at room temperature. The mixture was filtered on Celite, and the cake was rinsed with methanol. The filtrate was concentrated to give **21** (9 mg, 65%). ^1^H NMR (600 MHz, CDCl_3_): δ 9.28 (bs, 1H), 7.96, 7.88, 7.82, 7.71, 7.64 (5× s, 1H), 7.31–7.26 (m, 2H), 7.24–7.17 (m, 2H), 7.16–7.11 (m, 1H), 5.24–5.09 (m, 1H), 5.00–4.90 (m, 1H), 4.71–4.50 (m, 1H), 4.40–3.99 (m, 3H), 2.41–2.25 (m, 1H), 2.19–2.02 (m, 2H), 1.94–1.49 (m, 6H, includes singlets at 1.89, 1.87, 1.86, 1.85 and 1.83), 1.38–1.13 (m, 9H). ^13^C{^1^H} NMR (150 MHz, CDCl_3_): δ 173.8–173.0 (m), 170.3, 170.0, 169.8, 169.7, 169.6, 169.4, 164.43, 164.39, 164.36, 151.32, 151.28, 150.2–149.7 (m), 139.3, 139.2, 139.15, 139.0, 138.8, 138.63, 138.59, 138.5, 129.8, 129.7, 129.69, 129.66, 129.6, 125.3, 125.25, 125.2, 120.7–120.3 (m), 111.5, 111.43, 111.41, 111.40, 83.0–82.4 (m), 76.3–74.7 (m), 69.6, 69.54, 69.46, 69.38, 69.37, 53.78, 53.75, 53.7, 53.65, 53.5, 50.4, 50.3, 50.1, 50.0, 49.8, 38.9, 38.8, 38.5, 38.22, 38.18, 38.14, 37.9, 31.27, 31.24, 31.22, 31.00, 30.97, 30.1, 30.05, 30.03, 29.96, 29.8, 29.74, 29.67, 21.6–20.8 (m), 12.21, 12.19, 12.15, 12.11. ^31^P{^1^H} NMR (121 MHz, CDCl_3_): δ 17.70–16.95 (several lines). HRMS (ES+) *m*/*z*: [M + H]^+^ calcd for C_24_H_33_N_3_O_9_P 538.1954; found 538.1961.

### *cis*-1-((Benzyloxycarbonyl)bis(3-(hexadecyloxy)propyl)phosphonomethoxy)-4-acetoxycyclopent-2-ene **24**

A solution of methyl phosphonate **13** (250 mg, 0.63 mmol) and lutidine (467 μL, 405 mg, 3.78 mmol) in MeCN (3.5 mL) was treated dropwise with TMSBr (415 μL, 482 mg, 3.15 mmol). The resulting mixture was irradiated (50 °C, 50 W) for 30 min, after which it was concentrated under reduced pressure. The residue was dissolved in CH_2_Cl_2_ (5 mL), and oxalyl chloride (2 M in CH_2_Cl_2_, 0.95 mL, 1.9 mmol) was slowly added, followed by one drop of DMF. The mixture was stirred at room temperature for 3 h and again concentrated under reduced pressure. The residue was dissolved in CH_2_Cl_2_ (5 mL) and cooled in an ice-water bath, a solution of HDP-OH (416 mg, 1.39 mmol) and Hünig’s base (242 μL, 180 mg, 1.39 mmol) in CH_2_Cl_2_ (1 mL) was added dropwise, and the resulting mixture was allowed to warm slowly overnight. The volatiles were removed under reduced pressure, and the residue was extracted with hexanes (3 × 25 mL). The combined hexane extracts were evaporated, and the residue was purified by flash chromatography (SiO_2_, 30% EtOAc/hexanes) to afford the desired product **24** as a colorless waxy solid (176 mg, 30% yield). ^1^H NMR (300 MHz, CDCl_3_): δ 7.41–7.30 (m, 5H), 6.10–5.99 (m, 2H), 5.48–5.43 (m, 1H), 5.30–5.20 (m, 2H), 4.71–4.66 (m, 0.5H) 4.63–4.58 (m, 0.5H), 4.50 (d, 0.5H, *J* = 19.7), 4.47 (d, 0.5H, *J* = 19.9), 4.23–4.12 (m, 4H), 3.42 (q, 4H, *J* = 6.2), 3.35 (td, 4H, *J* = 6.7, 2.7), 2.79–2.67 (m, 1H), 2.02, 2.00 (2× s, 3H), 1.93–1.69 (m, 5H), 1.58–1.46 (m, 4H), 1.33–1.20 (m, 52H), 0.88 (t, 6H, *J* = 6.7). ^13^C{^1^H} NMR (75 MHz, CDCl_3_): δ 170.6, 170.5, 167.6 (d, *J* = 3), 167.5 (d, *J* = 3), 135.3, 135.07, 135.04, 134.8, 134.7, 134.1, 128.52, 128.50, 128.44, 128.41, 84.0, 83.8, 76.3, 76.2, 74.2 (d, *J* = 159), 73.7 (d, *J* = 159), 71.2, 67.5, 67.4, 66.5, 65.1–64.9 (m), 36.8, 36.7, 31.9, 30.9, 30.8, 29.69, 29.65, 29.61, 29.59, 29.5, 29.3, 26.1, 22.6, 20.97, 20.94, 14.1. ^31^P{^1^H} NMR (121 MHz, CDCl_3_): δ 14.39, 14.15. HRMS (ES+) *m*/*z*: [M + H]^+^ calcd for C_54_H_96_O_10_P 935.6736; found 935.6752.

### *cis*-1-(4-(Benzyloxycarbonyl-bis(3-(hexadecyloxy)propyl)phosphonomethoxy)cyclopent-2-en-1-yl)thymine **25** (Bis-HDP Unsaturated T-α-CNP Benzyl Ester)

A degassed mixture of thymine (7.4 mg, 0.058 mmol) and Na_2_CO_3_ (6.8 mg, 0.06 mmol) in H_2_O (0.15 mL) and MeCN (0.15 mL) was irradiated for 10 min (50 °C, 50 W). To the resulting homogeneous mixture was added a degassed solution of **24** (50 mg, 53 mmol) in MeCN (2.5 mL), followed by Pd_2_(dba)_3_ (6 mg, ca. 0.007 mmol) and dppb (7 mg, ca. 0.016 mmol). This mixture was irradiated for 30 min (50 °C, 50 W), after which a second portion of Pd_2_(dba)_3_ (6 mg) and dppb (7 mg) was added, and the mixture was irradiated again for 30 min. The mixture was diluted with CH_2_Cl_2_ (25 mL), filtered, and concentrated. The residue was purified by chromatography (SiO_2_, 80% EtOAc/hexanes) to afford **25** as a colorless waxy solid (18 mg, 34%). ^1^H NMR: (300 MHz, CDCl_3_): δ 8.70 (bs, 1H), 7.42–7.30 (m, 5.5H), 7.26–7.22 (m, 0.5H), 6.28 (dt, 0.5H, *J* = 5.5, 1.9), 6.19 (dt, 0.5H, *J* = 5.5, 1.8), 5.91 (dd, 0.5H, *J* = 5.5, 2.0), 5.86 (dd, 0.5H, *J* = 5.5, 2.2), 5.69–5.59 (m, 1H), 5.31–5.16 (m, 2H), 4.67–4.60 (m, 1H), 4.49 (d, 0.5H, *J* = 18.9), 4.48 (d. 0.5H, *J* = 19.5), 4.25–4.10 (m, 4H), 3.46–3.29 (m, 8H), 2.82–2.67 (m, 1H), 1.914, 1.910 (2× s, 3H) 1.91–1.68 (m, 5H), 1.59–1.43 (m, 4H), 1.33–1.20 (m, 52H), 0.87 (t, 6H, *J* = 6.7). ^13^C{^1^H} NMR: (75 MHz, CDCl_3_): δ 167.3 (d, *J* = 2), 167.0 (d, *J* = 2), 163.7, 150.89, 150.87, 137.3, 137.2, 135.8, 135.6, 134.9, 134.8, 134.64, 134.59, 128.7, 128.59, 128.57, 128.5, 111.6, 84.8 (d, *J* = 10), 84.7 (d, *J* = 11), 75.4 (d, *J* = 159), 75.2 (d, *J* = 159), 71.2, 67.7, 66.4, 66.3, 65.19 (d, *J* = 7), 65.16 (d, *J* = 7), 64.9, 64.8, 57.8, 57.7, 37.0, 36.9, 31.9, 30.9, 30.8, 29.7, 29.6, 29.5, 29.3, 26.1, 22.7, 14.1, 12.2. ^31^P{^1^H} NMR: (121 MHz, CDCl_3_): δ 14.03, 13.83. HRMS (ES+) *m*/*z*: [M + H]^+^ calcd for C_57_H_98_N_2_O_10_P 1001.6954; found 1001.6985.

### *cis*-1-(4-(Carboxy-bis(3-(methoxy)propyl)phosphonomethoxy)cyclopent-2-en-1-yl)thymine **26** (Bis-HDP T-α-CNP Free Acid)

A mixture of **25** (27 mg, 0.027 mmol) and 5% palladium on carbon (5 mg) in methanol (5 mL) and CH_2_Cl_2_ (1 mL) was stirred under a balloon of hydrogen at atmospheric pressure for 18 h at room temperature. The mixture was filtered through a syringe filter and concentrated to afford 22 mg (90%) of **26** as a colorless waxy solid. ^1^H NMR: (300 MHz, CDCl_3_): δ 8.52, 8.47 (2× bs, 1H), 7.85 (s, 0.5H), 7.65 (s, 0.5H), 5.29–5.14 (m, 1H), 4.42 (d, 0.5H, *J* = 20.3), 4.38 (d, 0.5H, *J* = 19.3), 4.34–4.18 (m, 5H), 3.50 (t, 4H, *J* = 6.0), 3.39 (t, 4H, *J* = 6.7), 2.45–2.30 (m, 1H), 2.27–2.04 (m, 2H), 2.02–1.77 (m, 5H, contains singlets at 1.96 and 1.95), 1.68–1.47 (m, 5H), 1.36–1.20 (m, 54H), 0.88 (t, 6H, *J* = 6.6). ^13^C{^1^H} NMR: (75 MHz, CDCl_3_): δ 169.1 (d, *J* = 3), 168.8 (d, *J* = 3) 164.24, 164.20, 151.26, 151.23, 138.9, 138.5, 111.6, 111.5, 82.6–82.2 (m), 73.9 (d, *J* = 158), 73.8 (d, *J* = 159), 71.3, 66.4, 65.5–65.2 (m), 53.4, 38.7, 38.4, 31.9, 31.1, 31.0, 30.84, 30.77, 30.2, 30.0, 29.7, 29.6, 29.5, 29.3, 26.1, 22.7, 14.1, 12.22, 12.18. ^31^P{^1^H} NMR: (121 MHz, CDCl_3_): δ 15.55, 14.47. HRMS (ES+) *m*/*z*: [M + H]^+^ calcd for C_50_H_94_N_2_O_10_P 913.6641; found 913.6648.

### *tert*-Butyl (Dimethylphosphono)diazoacetate **27**

A solution of *tert*-butyl dimethylphosphonoacetate (10.0 mL, 11.3 g, 50.4 mmol) and 4-acetamidobenzenesulfonyl azide (12.1 g, 50.4 mmol) in DMSO (50 mL) was stirred at room temperature and treated dropwise with DBU (8.5 mL, 8.64 g, 56.8 mmol) (mild exotherm). Stirring was continued for 15 min, after which water (50 mL) and Et_2_O (50 mL) were added. The organic layer was separated, and the aqueous layer was extracted with Et_2_O (5 × 20 mL). The combined organic phases were washed with brine (10 mL), dried over MgSO_4_, concentrated, and eluted over a short plug of silica gel (50% EtOAc/hexanes) to afford 8.2 g (65%) of **27** as a yellow oil with spectroscopic properties in accordance with the literature.^70^^1^H NMR (400 MHz, CDCl_3_): δ 3.83 (d, 6H, *J* = 12.0), 1.50 (s, 9H). ^13^C{^1^H} NMR (100 MHz, CDCl_3_): δ 162.1 (d, *J* = 12.3), 83.1, 53.6 (d, *J* = 5.8), 28.1 (signal for CN_2_ not observed). ^31^P{^1^H} NMR (162 MHz, CDCl_3_): δ 14.28. HRMS (ES+) *m*/*z*: [M + Na]^+^ calcd for C_8_H_15_N_2_O_5_PNa 273.0611; found 273.0614.

### *cis*-1-[(*tert*-Butoxycarbonyl)dimethylphosphonomethoxy]-4-acetoxycyclopent-2-ene **28**

*Rhodium-Catalyzed Reaction*: A solution of *tert*-butyl (dimethylphosphono)diazoacetate **27** (1.40 g, 5.60 mmol) and *cis*-4-hydroxy-2-cyclopentenyl acetate **12** (0.68 g, 4.78 mmol) in CH_2_Cl_2_ (30 mL) was purged with nitrogen gas for 10 min, after which activated 4 Å molecular sieve beads (ca. 1 mL) were added, and the mixture was stirred gently. After 1 h, Rh_2_(piv)_4_ (34 mg, 56 μmol, 1 mol %) was added, the mixture was placed in a pre-equilibrated bath set to 60 °C, and stirring was continued under reflux overnight. The mixture was cooled, decanted, concentrated under reduced pressure, and the residue was purified by flash chromatography (SiO_2_, 80% EtOAc/hexane) to afford the desired product **28** as a colorless oil. Yield 1.15 g (66%).

*Copper-Catalyzed Reaction*: A solution of *tert*-butyl (dimethylphosphono)diazoacetate **27** (7.0 g, 28.0 mmol) and *cis*-4-hydroxy-2-cyclopentenyl acetate **12** (3.29 g, 23.1 mmol) in benzene (100 mL) was purged with nitrogen gas for 15 min, after which activated 4 Å molecular sieve beads (ca. 10 mL) were added and the mixture was left to stand overnight. Copper(II) triflate (1.0 g, 2.8 mmol) was added, and the mixture was placed in a preheated 80 °C heating block. After 30 min, no more gas evolution was evident. The mixture was cooled, filtered, and concentrated, and the residue was purified by flash chromatography (SiO_2_, 80% EtOAc/hexane) to afford **28** as a colorless oil (3.45 g, 41%) ^1^H NMR (400 MHz, CDCl_3_): δ 6.15–6.11 (m, 1H), 6.07–6.03 (m, 1H), 5.49–5.45 (m, 1H), 4.70–4.67 (m, 0.5H), 4.62–4.59 (m, 0.5H), 4.38 (d, 0.5H, *J* = 19.5), 4.35 (d, 0.5H, *J* = 19.8), 3.86–3.82 (m, 6H), 2.81–2.74 (m, 1H), 2.05, 2.04 (2× s, 3H), 1.86 (dt, 0.5H, *J* = 14.9, 4.0), 1.74 (dt, 0.5H, *J* = 14.4, 4.1), 1.52, 1.50 (2× s, 9H). ^13^C{^1^H} NMR (100 MHz, CDCl_3_): δ 170.60, 170.57, 166.5 (d, *J* = 2.5), 166.4 (d, *J* = 2.3), 135.5, 134.7, 134.6, 133.9, 83.8 (d, *J* = 12.1), 83.7 (d, *J* = 12.6), 83.1, 82.9, 76.3, 76.2 74.4 (d, *J* = 159.9), 74.0 (d, *J* = 158.5), 54.0–53.9 (m), 36.9, 36.7, 27.84, 27.81, 21.0. ^31^P{^1^H} NMR (162 MHz, CDCl_3_): δ 17.58, 17.32. HRMS (ES+) *m*/*z*: [M + Na]^+^ calcd for C_15_H_25_O_8_PNa 387.1179; found 387.1176.

### *cis*-1-(4-((*tert*-Butoxycarbonyl)dimethylphosphonomethoxy)cyclopent-2-en-1-yl)thymine **29**

A solution of aq. sodium carbonate (2 M, 2.3 mL, 4.6 mmol) was added to a stirring solution of thymine (0.53 g, 4.22 mmol) and allylic acetate **28** (1.40 g, 3.84 mmol) in *N*,*N*-dimethylformamide (45 mL), and the mixture was purged with nitrogen gas for 20 min. Solid Pd(dba)_2_ (163 mg, 0.283 mmol) and 1,4-bis(diphenylphosphino)butane (163 mg, 0.384 mmol) were added, and the mixture was placed in a preheated heating block at 65 °C. The reaction was monitored by TLC, and after 30 min, no acetate remained. The DMF was removed in vacuo, and the residue was dissolved in CH_2_Cl_2_, filtered, concentrated, and purified by flash chromatography (SiO_2_, 5% MeOH/CH_2_Cl_2_) to afford the desired product **29** as an off-white foam (1.05 g, 63%).

*Alternative Extractive Workup*: The reaction was carried out as above, starting with **28** (1.0 g, 2.74 mmol), thymine (0.38 g, 3.01 mmol), Na_2_CO_3_ (2 M, 1.65 mL, 3.30 mmol), Pd_2_(dba)_3_ (93 mg, 0.10 mmol), and dppb (116 mg, 0.27 mmol) in DMF (30 mL). The cooled reaction mixture was partitioned with Et_2_O and 5% LiCl (150 mL each) and filtered over Celite. The phases were separated, and the aqueous phase was washed with Et_2_O. The combined organic phases were back-extracted with water, and the combined aqueous phases were then extracted with CH_2_Cl_2_ (5 × 20 mL). The solvents were evaporated, and the remaining DMF was removed as an azeotrope with toluene. The residue was purified by flash chromatography (SiO_2_, 5% MeOH/CH_2_Cl_2_) to afford 0.65 g (55%) of **29** as a white solid, shown by ^1^H NMR to be ca. 4:1 dr. This was dissolved in CH_2_Cl_2_ (25 mL) and three drops of Et_3_N were added. After standing for 24 h at room temperature, the mixture was evaporated and the residue was passed through a short plug of SiO_2_, eluting with 5% MeOH/CH_2_Cl_2_. Recovery 0.61 g, dr 1:1 (overall yield 52%). ^1^H NMR (400 MHz, CDCl_3_): δ 8.66, 8.65 (2× overlapping bs, 1H), 7.35 (s, 0.5H), 7.29 (s, 0.5H), 6.34–6.26 (m, 1H), 5.97–5.89 (m, 1H), 5.70–5.62 (m, 1H), 4.68–4.62 (m, 0.5H), 4.61–4.54 (m, 0.5H), 4.39 (d, 0.5H, *J* = 18.7), 4.35 (d, 0.5H, *J* = 19.5), 3.89–3.78 (m, 6H), 2.85–2.73 (m, 1H), 1.93 (s, 3H), 1.85–1.73 (m, 1H), 1.52, 1.50 (2× s, 9H). ^13^C{^1^H} NMR (100 MHz, CDCl_3_): δ 166.2, 165.9, 164.1, 151.1, 137.3, 137.2, 136.1, 135.5, 134.6, 134.3, 111.5, 111.4, 84.5 (d, *J* = 12.3), 84.3 (d, *J* = 10.7), 83.4, 75.2 (d, *J* = 158.3), 57.72, 57.67, 54.0–53.7 (m), 37.1, 36.8, 27.8, 12.2, 12.1. ^31^P{^1^H} NMR (162 MHz, CDCl_3_): δ 17.32, 17.13. HRMS (ES+) *m*/*z*: [M + Na]^+^ calcd for C_18_H_27_N_2_O_8_P 453.1397; found 453.1394.

### *cis*-1-(4-((*tert*-Butoxycarbonyl)dimethylphosphonomethoxy)cyclopentan-1-yl)thymine **30**

A solution of the alkene **29** (0.91 g) in methanol (50 mL) was placed under nitrogen, and 5% palladium on carbon (110 mg) was added. The flask was flushed with hydrogen, and the mixture was stirred overnight under a balloon of hydrogen. The mixture was filtered over Celite, concentrated, and the residue was passed over a short plug of silica gel eluting with 5% MeOH/CH_2_Cl_2_ to afford the desired product **30** as a white solid (0.83 g, 91%). ^1^H NMR (400 MHz, CDCl_3_): δ 8.15 (br s, 1H), 7.87 (unresolved q, 0.5 H, *J* ∼ 1), 7.72 (unresolved q, 0.5H, *J* ∼ 1), 5.31–5.20 (m, 1H), 4.32 (d, 0.5H, *J* = 19.5), 4.26 (d, 0.5H, *J* = 18.9), 4.21–4.16 (m, 1H), 3.88–3.82 (m, 6H), 2.44–2.31 (m, 1H), 2.26–2.16 (m, 1H), 2.15–1.6 (m, 7H contains 2.00, 1.98 2× unresolved d, 3H, *J* ∼ 1), 1.52, 1.51 (2× s, 9H). ^13^C{^1^H} NMR (100 MHz, CDCl_3_): δ 166.3 (d, *J* = 2.4), 166.0 (d, *J* = 1.6), 164.0, 151.49, 151.46, 138.3, 138.1, 111.65, 111.60, 83.5, 83.3, 82.8 (d, *J* = 9.6), 81.6 (d, *J* = 11.5), 74.8 (d, *J* = 158.4), 73.6 (d, *J* = 159.4), 54.1–53.6 (m), 53.1, 52.9, 38.5, 38.4, 31.5, 30.6, 30.1, 30.0, 27.7, 27.8, 12.22, 12.15. ^31^P{^1^H} NMR (162 MHz, CDCl_3_) δ: 17.84, 17.57. HRMS (ES+) *m*/*z*: [M + Na]^+^ calcd for C_18_H_29_N_2_O_8_PNa 455.1554; found 455.1557.

### *tert*-Butyl 2-(8-(*tert*-Butyl)-2-oxido-4*H*-benzo[*d*][1,3,2]dioxaphosphinin-2-yl)-2-((3-(5-methyl-2,4-dioxo-3,4-dihydropyrimidin-1(2*H*)-yl)cyclopentyl)oxy)acetate **34** (3-*tert*-Butylcyclosal T-α-CNP *tert*-Butyl Ester)

A solution of the dimethyl phosphonate **30** (250 mg, 0.578 mmol) and 2,6-lutidine (402 μL, 372 mg, 3.47 mmol) in MeCN (3.5 mL) was added dropwise to bromotrimethylsilane (381 μL, 442 mg, 2.89 mmol). The resulting solution was irradiated (50 W, 50 °C) for 30 min, after which the volatiles were removed in vacuo. The residue was redissolved in CH_2_Cl_2_ (5 mL), and two drops of DMF were added, followed by the dropwise addition of oxalyl chloride (2 M in CH_2_Cl_2_, 0.87 mL, 1.73 mmol). The resulting mixture was stirred at room temperature for 3 h and again evaporated in vacuo. The residue was redissolved in CH_2_Cl_2_ (5 mL), cooled in ice, and treated dropwise with a solution of 2-(*tert*-butyl)-6-(hydroxymethyl)phenol **31** (113 mg, 0.629 mmol) and triethylamine (177 μL, 129 mg, 1.27 mmol) in CH_2_Cl_2_ (1 mL); after the addition, the mixture was allowed to warm slowly to room temperature overnight. Silica gel (ca. 1 g) was added, the mixture was concentrated under reduced pressure, and the residue was purified by flash chromatography (80% EtOAc/hexanes) to afford the desired product **34** as a colorless film that was a roughly equal mixture of four diastereomers (47 mg, 15%). ^1^H NMR (600 MHz, CDCl_3_): δ 9.25 (bs, ∼0.5H), 9.21 (bs, ∼0.5H), 7.76 (s, ∼0.6H), 7.65 (s, ∼0.2H), 7.59 (s, ∼0.2H), 7.37–7.29 (m, 1 H), 7.09–7.01 (m, 1H), 6.98–6.86 (m, 1H), 5.45–5.10 (m, 3H), 4.56 (d, ∼0.2H, *J* = 20.0), 4.54 (d, ∼0.2H, *J* = 19.4), 4.52 (d, ∼0.3H, *J* = 19.1), 4.50 (d, ∼0.3H, *J* = 19.4), 4.26 (bt, ∼0.3H, *J* = 4.3), 4.17 (bt, ∼0.2H, *J* = 4.4), 4.10 (bt, ∼0.5H, *J* = 4.4), 2.40–1.23 (m, 28H, includes singlets at 1.97, 1.93, 1.90, 1.84, 1.43, 1.42, 1.414, 1.410, 1.407, 1.40, 1.35). ^13^C{^1^H} NMR (150 MHz, CDCl_3_): δ 165.8 (d, *J* = 3.5), 165.55 (d, *J* = 3.3), 165.50 br, 163.98, 163.96, 163.93, 151.44, 151.43, 151.40, 149.1 (d, *J* = 9.0), 149.0 (d, *J* = 9.0), 148.8 (d, *J* = 9.7), 148.4 (d, *J* = 9.1), 139.5 (d, *J* = 5.4), 139.45 (d, *J* = 5.7), 139.37 (d, *J* = 5.7), 139.2 (d, *J* = 5.6), 138.3, 138.1, 138.0, 137.8, 127.95, 127.93, 127.7, 127.6, 124.2, 124.15, 124.13, 123.80, 123.78, 123.6, 123.4, 123.0 (d, *J* = 9.0), 122.7 (d, *J* = 8.9), 122.4 (d, *J* = 9.8), 122.3 (d, *J* = 9.5), 111 73, 111.67, 111.6, 111.2, 84.09, 84.06, 83.97, 83.93, 83.4 (d, *J* = 9.6), 83.0 (d, *J* = 8.8), 82.7 (d, *J* = 8.3), 82.6 (d, *J* = 9.1), 75.8 (d, *J* = 157.3), 75.6 (d, *J* = 158.4), 75.0 (d, *J* = 159.0), 74.7 (d, *J* = 160.5), 68.5 (d, *J* = 7.6), 68.4 (d, *J* = 7.5), 68.2 (d, *J* = 7.3), 67.8 (d, *J* = 7.4), 53.2, 53.15, 53.10, 53.07, 38.8, 38.5, 38.2, 37.9, 34.82, 34.80, 34.76, 31.2, 31.15, 31.1. 31.0, 30.1, 29.97, 29.94, 29.92, 29.8, 29.4, 27.74, 27.71, 27.69, 12.4, 12.21, 12.18, 12.1. ^31^P{^1^H} NMR (121 MHz, CDCl_3_): δ 9.00, 8.93, 6.89, 6.80. HRMS (ES+) *m*/*z*: [M + Na]^+^ calcd for C_27_H_37_N_2_O_8_PNa 571.2180; found 571.2179.

### *tert*-Butyl 2-(8-Methyl-2-oxido-4*H*-benzo[*d*][1,3,2]dioxaphosphinin-2-yl)-2-((3-(5-methyl-2,4-dioxo-3,4-dihydropyrimidin-1(2*H*)-yl)cyclopentyl)oxy)acetate **35** (3-Methylcyclosal T-α-CNP *tert*-Butyl Ester)

This was prepared following the procedure described for **34**, starting from phosphonate **30** (250 mg, 0.578 mmol), 2,6-lutidine (381 μL, 442 mg, 2.89 mmol), and bromotrimethylsilane (381 μL, 442 mg, 2.89 mmol) in MeCN (3.5 mL); DMF (2 drops) and oxalyl chloride (2 M in CH_2_Cl_2_, 0.87 mL, 1.73 mmol) in CH_2_Cl_2_; and 2-methyl-6-(hydroxymethyl)phenol **32** (86 mg, 0.629 mmol) and triethylamine (177 μL, 129 mg, 1.27 mmol) in CH_2_Cl_2_ (5 mL + 1 mL). Yield **35**, colorless film, mixture of diastereomers, 32 mg (11%) ^1^H NMR (600 MHz, CDCl_3_): δ 9.05 (bs, 1H), 7.70 (s, ∼0.3H), 7.65 (s, ∼0.4H), 7.54 (s, ∼0.3H), 7.21–7.14 (m, 1H), 7.05–6.97 (m, 1H), 6.95–6.86 (m, 1H), 5.48–5.40 (m, 1H), 5.30–5.10 (m, 2H), 4.482 (d, ∼0.3H, *J* = 20.1), 4.478 (d, ∼0.3H, *J* = 19.7), 4.67 (d, ∼0.15H, *J* = 18.5), 4.46 (d, ∼0.25H, *J* = 19.6), 4.13 (bt, ∼0.15H, *J* = 4.4), 4.11 (bt, ∼0.25H, *J* = 4.5), 4.07 (bt, 0.3H, *J* = 4.6), 4.05 (bt, ∼0.3H, *J* = 4.2), 2.37–1.03 (m, 21H, includes singlets at 2.31, 2.30, 2.29, 1.96, 1.95, 1.94, 1.41, 1.37, 1.34, 1.30). ^13^C{^1^H} NMR (150 MHz, CDCl_3_): δ 165.5 (d, *J* = 4), 165.34 (d, *J* = 3), 165.32 (d, *J* = 3), 165.2 (d, *J* = 4), 163.9, 163.87, 163.84, 151.36, 151.33, 151.31, 149.2 (d, *J* = 8.5), 148.9–148.8 (m), 138.4, 138.1, 138.0, 137.8, 131.41, 131.38, 131.34, 131.32, 127.90 (d, *J* = 6.8), 127.86 (d, *J* = 7.2), 127.6 (d, *J* = 6.6), 127.3 (d, *J* = 6.6), 124.1, 124.05, 124.00, 123.95, 123.0, 122.9, 122.8, 121.6 (d, *J* = 8.7), 121.5 (d. *J* = 8.8), 121.43 (d, *J* = 9.6), 121.37 (d, *J* = 8.9), 111.6, 111.5, 111.1, 84.03, 83.95, 83.94, 83.84, 83.5 (d, *J* = 8.3), 82.6–82.4 (m), 75.3 (d, *J* = 156.8), 74.7 (d, *J* = 158.6), 74.44 (d, *J* = 159.2), 74.36 (d, *J* = 157.6), 68.1 (d, *J* = 7.7), 67.9 (d, *J* = 7.5), 67.7 (d, *J* = 7.6), 67.4 (d, *J* = 7.6), 53.1, 53.04, 52.99, 38.7, 38.2, 38.03, 38.00, 31.19, 31.17, 31.11, 31.05, 30.0, 29.9, 29.8, 29.4, 27.7, 27.6, 27.5, 15.43, 15.40, 15.35, 15.34, 12.4, 12.3, 12.2. ^31^P{^1^H} NMR (121 MHz, CDCl_3_): δ 9.15, 9.06, 8.07. HRMS (ES+) *m*/*z*: [M + Na]^+^ calcd for C_24_H_31_N_2_O_8_PNa 529.1710; found 529.1706

### *tert*-Butyl 2-(6,8-Dimethyl-2-oxido-4*H*-benzo[*d*][1,3,2]dioxaphosphinin-2-yl)-2-((3-(5-methyl-2,4-dioxo-3,4-dihydropyrimidin-1(2*H*)-yl)cyclopentyl)oxy)acetate **36** (3,5-Dimethylcyclosal T-α-CNP *tert*-Butyl Ester)

This was prepared following the procedure described for **34**, starting from phosphonate **30** (250 mg, 0.578 mmol), 2,6-lutidine (381 μL, 442 mg, 2.89 mmol) and bromotrimethylsilane (381 μL, 442 mg, 2.89 mmol) in MeCN (3.5 mL); DMF (2 drops) and oxalyl chloride (2 M in CH_2_Cl_2_, 0.87 mL, 1.73 mmol) in CH_2_Cl_2_; 2,4-dimethyl-6-(hydroxymethyl)phenol **33** (95 mg, 0.629 mmol) and triethylamine (177 μL, 129 mg, 1.27 mmol) in CH_2_Cl_2_ (5 mL + 1 mL). Yield **36**, colorless film (approx 1:1:2:2 mixture of diastereomers) 85 mg (28%). ^1^H NMR (600 MHz, CDCl_3_): δ 8.98 (b s, 1H), 7.73 (s, ∼0.2 H), 7.691, 7.686 (2× s, ∼0.7H), 7.58 (2, ∼0.1 H), 7.00–6.93 (m, 1H), 6.73–6.65 (m, 1H), 5.43–5.34 (m, 1H), 5.24–5.11 (m, 2H), 4.48–4.42 (m, 1H), 4.15–4.03 (m, 1H) 2.40–1.06 (m. 24H, contains singlets at 2.26, 2.25, 2.24, 2.22, 1.96, 1.95, 1.94, 1.93, 1.41, 1.38, 1.35, 1.30). ^13^C{^1^H} NMR (150 MHz, CDCl_3_): δ 165.6 (d, *J* = 3.9), 165.4–165.35 (m), 163.9, 163.86, 163.84, 151.4, 151.33, 151.30, 147.0 (d, *J* = 8.1), 146.6 (d, *J* = 8.6), 146.5 (d, *J* = 8.7), 138.5, 138.2, 138.15, 137.9, 133.74, 133.71, 133.66, 133.61, 132.0, 131.94, 131.91, 131.8, 127.45 (d, *J* = 6.5), 127.43 (d, *J* = 6.4), 127.3 (d, *J* = 6.5), 126.9 (d, *J* = 6.4), 123.3, 123.23, 123.17, 123.10, 121.20 (d, *J* = 8.6), 121.17 (d, *J* = 8.9), 121.0 (d, *J* = 8.7), 120.9 (d, *J* = 8.7), 111.7, 111.6, 111.5, 111.0, 84.0, 83.9, 83.8, 83.5 (d, *J* = 8.0), 82.6–84.4 (m), 75.4 (d, *J* = 157.2), 74.7 (d, *J* = 158.7), 74.5 (d, *J* = 157.8), 74.4 (d, *J* = 159.2), 68.2 (d, *J* = 7.7), 68.0 (d, *J* = 7.5), 67.8 (d, *J* = 7.5), 67.5 (d, *J* = 7.3), 53.13, 53.08, 38.8, 38.3, 38.1, 38.0, 31.2, 31.13, 31.10, 30.03, 29.93, 29.8, 29.3, 27.7, 27.6, 27.5, 20.55, 20.50, 15.34, 15.32, 15.27, 12.4, 12.3, 12.2. ^31^P{^1^H} NMR (125 MHz, CDCl_3_): δ 9.04, 8.91, 8.24, 8.14. HRMS (ES+) *m*/*z*: [M + Na]^+^ calcd for C_25_H_33_N_2_O_8_PNa 574.1867; found 543.1867

### 2-(8-(*tert*-Butyl)-2-oxido-4*H*-benzo[*d*][1,3,2]dioxaphosphinin-2-yl)-2-(((1*R*,3*S*)-3-(5-methyl-2,4-dioxo-3,4-dihydropyrimidin-1(2*H*)-yl)cyclopentyl)oxy)acetic Acid **37** (3-*tert*-Butylcyclosal T-α-CNP)

A solution of the *tert*-butyl ester **34** (40 mg) in CH_2_Cl_2_ (3 mL) was cooled in an ice-water bath, and trifluoroacetic acid (0.5 mL) was added dropwise. After the addition, the mixture was allowed to warm slowly while being stirred overnight, and then concentrated under reduced pressure. The residue was dissolved in CH_2_Cl_2_, filtered on a syringe filter, and then concentrated to afford the desired product **37** as a colorless film (36 mg, ∼quant.). ^1^H NMR (600 MHz, CDCl_3_): δ 9.60–9.15 (m, br, 1H), 7.88, 7.80, 7.64, 7.62 (4× s, 1H), 7.38–7.32 (m, 1H), 7.11–7.03 (m. 1H), 6.99–6.88 (m, 1H), 5.43–5.27 (m, 2H), 5.20–5.04 (m, 1H), 4.76–4.67 (m, 1H), 4.32–4.14 (m, 1H), 2.36–2.02 (m, 3H), 1.96–1.83 (m, 4H, contains singlets at 1.94, 1.91, 1.87, 1.84), 1.76–1.30 (m, 11H, contains singlets at 1.42, 1.41, 1.40, 1.39). ^13^C{^1^H} NMR (150 MHz, CDCl_3_): δ 169.0 (d, *J* = 4.3), 168.7 (d, *J* = 3.2), 168.4 (d, *J* = 4.7), 168.3 (d, *J* = 4.2), 164.72, 164.69, 164.65, 151.25, 151.21, 149.0 (d, *J* = 9.5), 148.8 (d, *J* = 7.9), 148.7 (d, *J* = 8.6), 148.5 (d, *J* = 9.1), 139.7 (d, *J* = 5.4), 139.6 (d, *J* = 5.6), 139.5 (d, *J* = 5.7), 139.4 (d, *J* = 5.6), 139.3, 139.1, 138.9, 138.8, 128.2, 127.9, 127.8, 124.51, 124.45, 124.43, 123.9, 123.8, 123.63, 123.57, 122.7 (d, *J* = 9.7), 122.6 (d, *J* = 9.0), 122.4 (d, *J* = 10.1), 122.3 (d, *J* = 10.3), 111.6, 111.5, 111.4, 111.1, 83.8 (d, *J* = 9.1), 83.6 (d, *J* = 11.1), 83.5 (d, *J* = 7.8), 82.9 (d, *J* = 6.5), 75.4 (d, *J* = 157.4), 74.7 (d, *J* = 159.4), 74.6 (d, *J* = 156.7), 73.7 (d, *J* = 157.4), 69.3 (d, *J* = 7.0), 69.2 (d, *J* = 7.1), 68.7 (d, *J* = 7.4), 68.4 (d, *J* = 7.2), 53.94, 53.91, 53.7, 53.6, 39.2, 38.7, 37.8, 37.7, 34.88, 34.87, 34.85, 34.80, 30.72, 30.68, 30.2, 30.1, 30.0, 29.95, 29.92, 29.74, 29.70, 29.4, 12.3, 12.2, 12.1. ^31^P{^1^H} NMR (121 MHz, CDCl_3_): δ 10.13, 10.08, 7.95, 7.55. HRMS (ES+) *m*/*z*: [M + H]^+^ calcd for C_23_H_30_N_2_O_8_P 493.1734; found 493.1730.

### 2-((3-(5-Methyl-2,4-dioxo-3,4-dihydropyrimidin-1(2*H*)-yl)cyclopentyl)oxy)-2-(8-methyl-2-oxido-4*H*-benzo[*d*][1,3,2]dioxaphosphinin-2-yl)acetic Acid **38** (3-Methylcyclosal T-α-CNP)

This was prepared following the procedure described for **37**, starting from *tert*-butyl ester **35** (28 mg), CH_2_Cl_2_ (3 mL), and trifluoroacetic acid (0.5 mL), to afford **38** as a colorless film (24 mg, ∼quantitative). ^1^H NMR (400 MHz, CDCl_3_): δ 9.35, 9.26 (2× br s, 1H), 7.80, 7.68, 7.61, 7.55 (4× s, 1H), 7.23–6.34 (m, 3H), 5.56–5.02 (m, 3H), 4.69–4.58 (m, 1H), 4.18–4.05 (m, 1H), 2.32, 2.31, 2.30 (3× s, 3H), 2.30–1.95 (m 3H), 1.92 (br s, 3H), 1.88–1.40 (m, 3H). ^13^C{^1^H} NMR (150 MHz, CDCl_3_) δ: 169.3–167.8 (m, br), 165.1, 151.4 (br), 149.1, 148.9, 148.5, 139.9, 139.4, 139.2, 139.1, 131.52, 131.49, 131.45, 128.1, 128.0, 127.8, 127.4, 124.42, 124.38, 124.3, 124.2, 123.13, 123.07, 122.9, 121.7, 121.5, 112.1–110.9 (m, br), 83.7, 83.3, 82.3, 75.7–73.5 (m, br), 68.8, 68.5, 68.3, 53.8, 53.6, 53.5, 39.0, 38.3, 37.9, 31.6, 31.3, 30.8, 30.6, 30.1, 30.0, 29.7, 29.3, 15.3, 15.2, 12.2, 12.1, 12.0. ^31^P{^1^H} NMR (162 MHz, CDCl_3_): δ 10.29, 9.83, 8.57, 8.53. HRMS (ES+) *m*/*z*: [M + H]^+^ calcd for C_20_H_24_N_2_O_8_P 451.1265; found 451.1262.

### 2-(6,8-Dimethyl-2-oxido-4*H*-benzo[*d*][1,3,2]dioxaphosphinin-2-yl)-2-((3-(5-methyl-2,4-dioxo-3,4-dihydropyrimidin-1(2*H*)-yl)cyclopentyl)oxy)acetic Acid **39** (3,5-Dimethylcyclosal T-α-CNP)

This was prepared following the procedure described for **34**, starting from of the *tert*-butyl ester **36** (42 mg), CH_2_Cl_2_ (3 mL), and trifluoroacetic acid (0.5 mL). Yield **39**, colorless film, 36 mg (∼quant.). ^1^H NMR (600 MHz, CDCl_3_): δ 9.89 (br s, 1H), 7.87, 7.77, 7.69, 7.62 (4× s, 1H), 7.00–6.94 (m, 1H), 6.76–6.66 (m, 1H), 5.50–5.38 (m, 1H), 5.32–5.07 (m, 2H), 4.69–4.61 (m, 1H), 4.18–4.07 (m, 1H), 2.39–1.95 (m, 9H, contains singlets at 2.26, 2.25, 2.24, 2.22, 2.20), 1.94–1.44 (m, 6H, contains singlets at 1.91, 1.90). ^13^C{^1^H} NMR (150 MHz, CDCl_3_): δ 168.9 (d, *J* = 3.8), 168.64 (d, *J* = 2.9), 168.56 (d, *J* = 3.1), 168.4 (d, *J* = 4.1), 165.13, 165.08, 153.3, 151.30, 151.27, 146.73 (d, *J* = 8.2), 146.65 (d, *J* = 8.0), 146.60 (d, *J* = 8.4), 146.2 (d, *J* = 7.8), 140.0, 139.6, 139.3, 139.2, 134.19, 134.16, 134.1, 134.0, 132.10, 132.07, 132.0, 127.6 (d, *J* = 6.2), 127.53 (d, *J* = 6.7), 127.48 (d, *J* = 7.0), 127.1 (d, *J* = 6.2), 123.37, 123.34, 123.3, 123.2, 121.3, 121.2, 121.1, 121.1, 111.53, 111.45, 111.40, 110.8, 83.7 (d, *J* = 7.7), 83.3 (d, *J* = 9.8), 82.4 (d, *J* = 5.1), 74.4 (d, *J* = 158.3), 74.3 (d, *J* = 156.6), 74.2 (d, *J* = 158.5), 68.92 (d, *J* = 7.8), 68.89 (d, *J* = 7.8), 68.6 (d *J* = 7.7), 68.4 (d, *J* = 7.4), 53.9, 53.7, 53.6, 39.1, 38.4, 37.9, 31.6, 31.3, 30.8, 30.6, 30.2, 30.0, 29.7, 29.2, 20.54, 20.53, 20.45, 15.15, 15.14, 15.10, 12.14, 12.10, 12.08, 11.99. Residual TFA visible at ∼160 ppm. ^31^P{^1^H} NMR (121 MHz, CDCl_3_): δ 10.61, 10.05, 9.13. HRMS (ES+) *m*/*z*: [M + H]^+^ calcd for C_21_H_26_N_2_O_8_P 465.1421; found 465.1434.

### 4-Nonanoyloxybenzyl Alcohol **40**

A solution of 4-hydroxybenzyl alcohol (2.0 g, 16.1 mmol) and triethylamine (2.5 mL, 1.79 g, 17.7 mmol) in CH_2_Cl_2_ (20 mL) was cooled in an ice bath and treated dropwise with nonanoyl chloride (3.3 mL, 3.13 g, 17.7 mmol). The resulting mixture was allowed to warm slowly and stirred overnight, after which the solvent was removed under reduced pressure. The residue was partitioned with EtOAc and saturated NaHCO_3_ (ca. 50 mL each) and stirred for 1 h. The organic layer was separated, the aqueous layer was extracted with ethyl acetate, and the combined organic phases were washed with brine, dried with MgSO_4_, and concentrated. The residue was purified by flash chromatography (SiO_2_, 30% EtOAc/hexanes) to afford the desired product **40** as a colorless microcrystalline solid. Yield 2.43 g (57%). ^1^H NMR (400 MHz, CDCl_3_): δ 7.40–7.35 (m, 2H), 7.09–7.04 (m, 2H), 4.68 (s, 2H), 2.55 (t, 2H, *J* = 7.5), 1.80–1.65 (m, 3H), 1.46–1.21 (m, 10H), 0.89 (t, 3H, *J* = 6.8). ^13^C{^1^H} NMR (100 MHz, CDCl_3_): δ 172.5, 150.0, 138.4, 127.9, 121.5, 64.5, 34.3, 31.7, 29.1, 29.05, 29.0, 24.9, 22.6, 14.0. HRMS (ES+) *m*/*z*: [M + Na]^+^ calcd for C_16_H_24_O_3_Na 287.1618; found 287.1611.

### 4-Nonanoyloxybenzyl Chloride **41**

Thionyl chloride (0.27 mL, 0.45 g, 3.78 mmol) was slowly added to a solution of the alcohol **40** (0.50 g, 1.89 mmol) in CH_2_Cl_2_ (10 mL), and the resulting mixture was stirred at room temperature for 90 min. The mixture was concentrated under reduced pressure and partitioned between ether and saturated NaHCO_3_ (20 mL each). The organic phase was separated, dried over MgSO_4_, and concentrated. The residue purified by flash chromatography (SiO_2_, 5% EtOAc/hexanes) to afford the desired product **41** (0.43 g, 79%) as a colorless liquid. ^1^H NMR (400 MHz, CDCl_3_): δ 7.42–7.37 (m, 2H), 7.10–7.05 (m, 2H), 4.58 (s, 2H), 2.55 (t, 2H, *J* = 7.5), 1.75 (quint, 2H, *J* = 7.5), 1.45–1.21 (m, 10H), 0.89 (t, 3H, *J* = 6.6). ^13^C{^1^H} NMR (100 MHz, CDCl_3_): δ 172.08, 150.64, 134.84, 129.65, 121.83, 45.50, 34.31, 31.74, 29.15, 29.06, 29.03, 24.85, 22.58, 14.04. HRMS (ES+) *m*/*z*: [M + H]^+^ calcd for C_16_H_24_^35^ClO_2_ 283.1459; found 283.1463.

### *cis*-1-(4-(*tert*-Butoxycarbonyl-di(4-nonanoyloxybenzyl)phosphonomethoxy)cyclopentan-1-yl)thymine **42**

A mixture of phosphonate **30** (100 mg, 0.231 mmol), 4-nonanoyloxybenzyl chloride **41** (156 mg, 0.552 mmol), and sodium iodide (70 mg, 0.467 mmol) in acetonitrile (2 mL) was heated in a sealed tube for 48 h. After cooling, the volatiles were removed under reduced pressure and the residue was purified by flash chromatography (80% EtOAc/hexanes) to give **42** as a colorless oil (30 mg, 14%). ^1^H NMR (400 MHz, CDCl_3_): δ 8.69 (bs, 1H), 7.82 (s, 0.5H), 7.65 (s, 0.5H), 7.40–7.31 (m, 4H), 7.09–7.00 (m, 4H), 5.27–5.14 (m, 1H), 5.14–5.00 (m, 4H), 4.25 (d, 0.5H, *J* = 19.0), 4.20 (d, 0.5H, *J* = 18.0), 4.15–4.05 (m, 1H), 2.54 (t, 4 H, *J* = 7.5), 2.39–2.22 (m, 1H), 2.20–2.05 (m, 1H), 2.01–1.65 (m, 10H, contains singlets at 1.93 and 1.89), 1.61–1.19 (m, 30H, contains singlets at 1.42 and 1.41), 0.88 (t, 6H, *J* = 6.7). ^13^C{^1^H} NMR (100 MHz, CDCl_3_): δ 172.09, 172.06, 166.1 (d, *J* = 2.5) 165.9 (d, *J* = 1.6), 163.9, 163.8, 151.4, 151.3, 150.96, 150.93, 150.91, 150.87, 138.4, 138.1, 133.21, 133.15, 133.04, 132.98, 132.92, 129.4, 129.3, 129.2, 121.8, 111.64, 111.56, 83.5, 83.3, 82.6 (d, *J* = 9.0), 81.8 (d, *J* = 11.1), 75.0 (d, *J* = 159.3), 74.5 (d, *J* = 160.0), 68.2–67.9 (m), 53.1, 53.0, 38.37, 38.35, 34.3, 31.7, 31.3, 30.8, 30.03, 30.00, 29.1, 29.04, 29.02, 27.83, 27.78, 24.8, 22.6, 14.0, 12.3, 12.2. ^31^P{^1^H} NMR (162 MHz, CDCl_3_): δ 16.40, 16.37. HRMS (ES+) *m*/*z*: [M + H]^+^ calcd for C_48_H_70_N_2_O_12_P 897.4661; found 897.4667.

### *cis*-1-(4-(Carboxy-di(4-nonanoyloxybenzyl)phosphonomethoxy)cyclopentan-1-yl)thymine **43**

A solution of the *tert*-butyl ester **42** (16 mg, 17.8 μmol) in CH_2_Cl_2_ (1 mL) was cooled in ice and treated with TFA (1 mL). The solution was stirred for 2 h and then concentrated under reduced pressure. The waxy residue was washed with hexane (2 × 1 mL) and 20% EtOAc/hexane (3 × 3 mL) and then extracted with Et_2_O (2 × 2 mL). The combined ether extracts were evaporated to afford the acid **43** as a colorless film (6 mg, 40%, estimated to be ca. 80% pure by NMR). ^1^H NMR (400 MHz, CDCl_3_): δ 9.09–8.54 (br m, 1H), 7.84, 7.59 (2× s, 1H), 7.42–7.31 (m, 4H), 7.08–6.98 (m, 4H), 5.22–5.03 (m, 5H), 4.38 (d, *J* = 20.3, 0.5H), 4.34 (d, 0.5H, *J* = 19.4), 4.30–4.10 (m, 1H, partly obscured by a broad hydroxylic signal), 2.54 (t, 4H, *J* = 7.5), 2.37–1.60 (m, 13H, contains singlets at 1.88 and 1.85), 1.59–1.17 (m, 24H), 0.88 (t, 6H, *J* = 6.3). ^31^P{^1^H} NMR (162 MHz, CDCl_3_): δ 16.84, 16.55. No ^13^C NMR data was recorded for this compound. HRMS (ES+) *m*/*z*: [M + H]^+^ calcd for C_44_H_62_N_2_O_12_P 841.4035; found 841.4049.

## Supporting Information Available

Copies of ^1^H, ^13^C, ^19^F, and ^31^P NMR spectra (PDF)
