# Supplementary material for: Synthesis and Evaluation of Prodrugs of α-Carboxy Nucleoside Phosphonates
Source: J Org Chem. 2022 Oct 25;87(21):14793–808. doi: 10.1021/acs.joc.2c02135 (PMC9639015; doi:10.1021/acs.joc.2c02135)

Supporting Information

Synthesis and evaluation of prodrugs of alpha-carboxy nucleoside phosphonates

Alan Ford,[†] Nicholas D. Mullins,[†] Jan Balzarini[‡] and Anita R. Maguire[†§]*

[†]School of Chemistry and [§]School of Pharmacy, Analytical and Biological Chemistry Research Facility, Synthesis and Solid State Pharmaceutical Centre, University College, Cork, Ireland

[‡]KU Leuven, Rega Institute for Medical Research, Herestraat 49, B-3000 Leuven, Belgium

Contents

Copies ^{1}H, ^{13}C, and ^{31}P and ^{19}F NMR spectra if applicable S2–S46

Compound **2** ^{1}H NMR (300 MHz, $CDCl_3$)

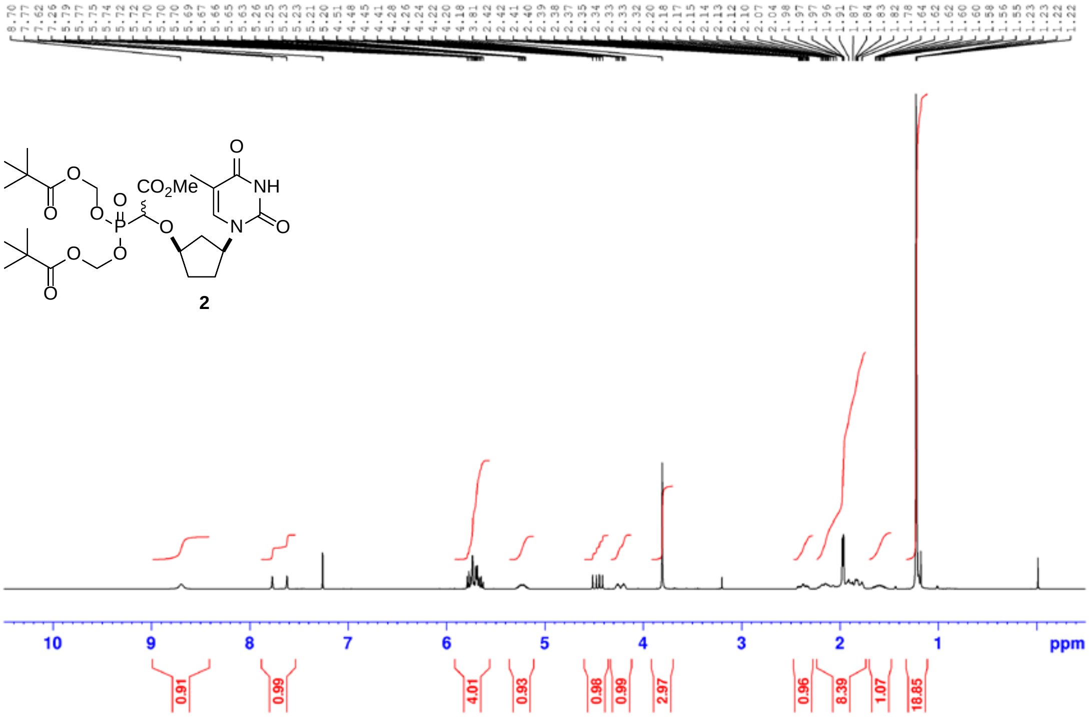

Compound **2** $^{12}C\{^{1}H\}$ NMR (75 MHz, $CDCl_3$)

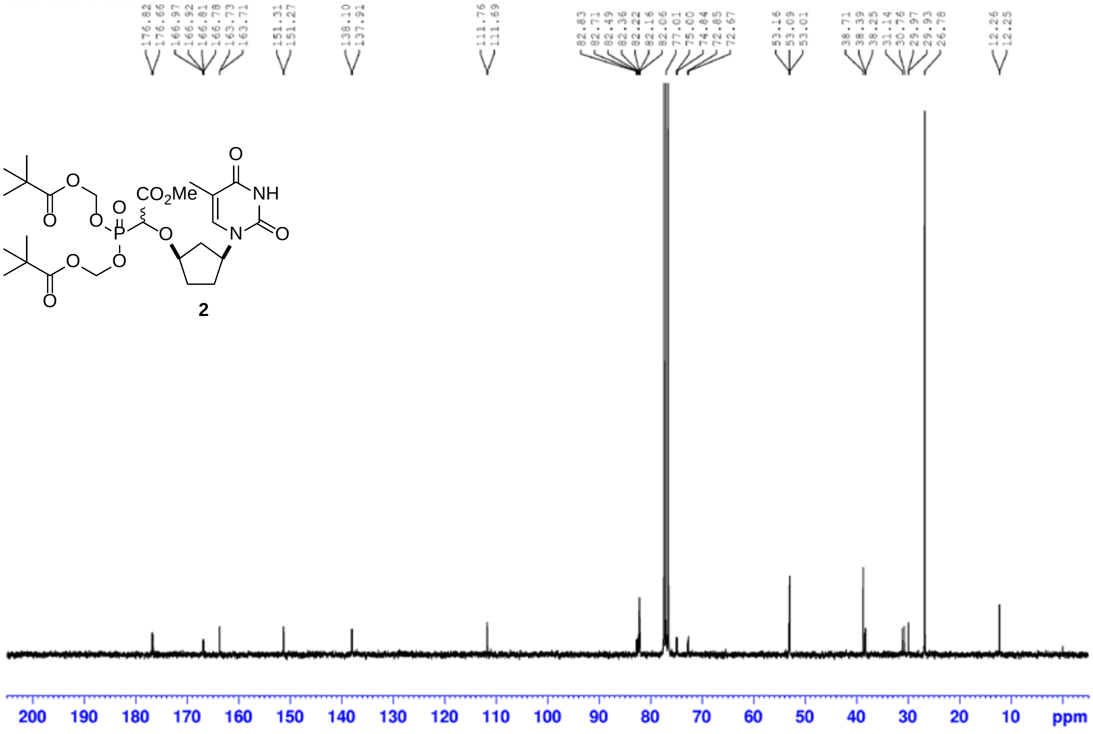

Compound **2** $^{31}P\{^{1}H\}$ NMR (121 MHz, $CDCl_3$)

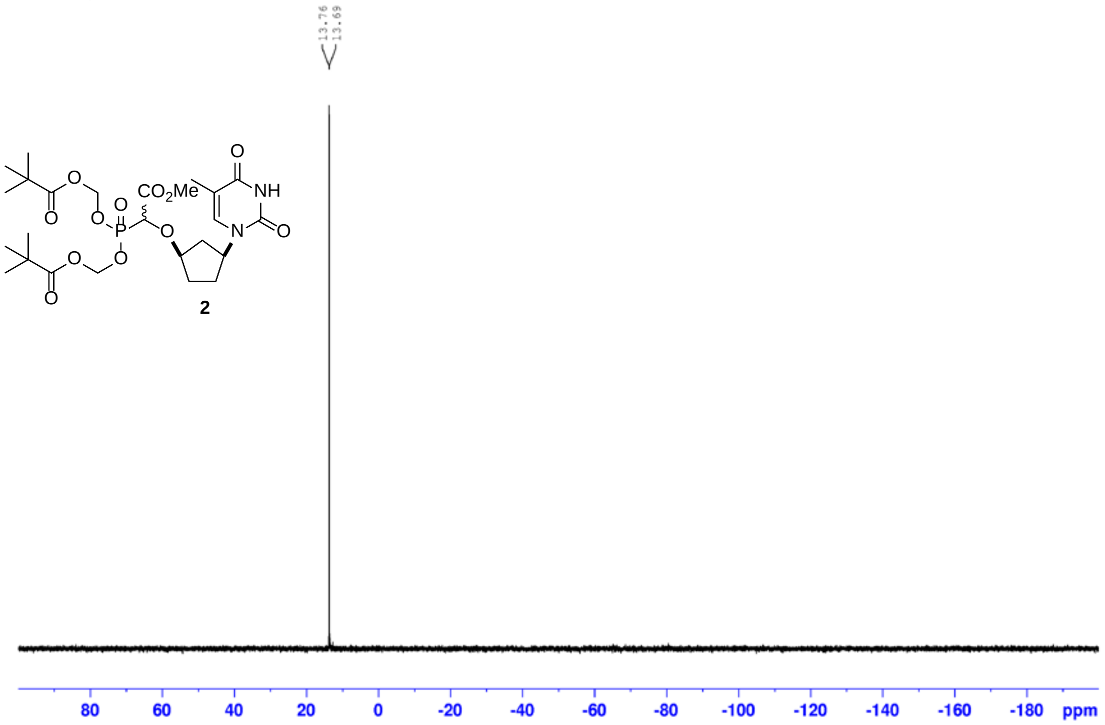

Compound **8** ^{1}H NMR (300 MHz, $CDCl_3$)

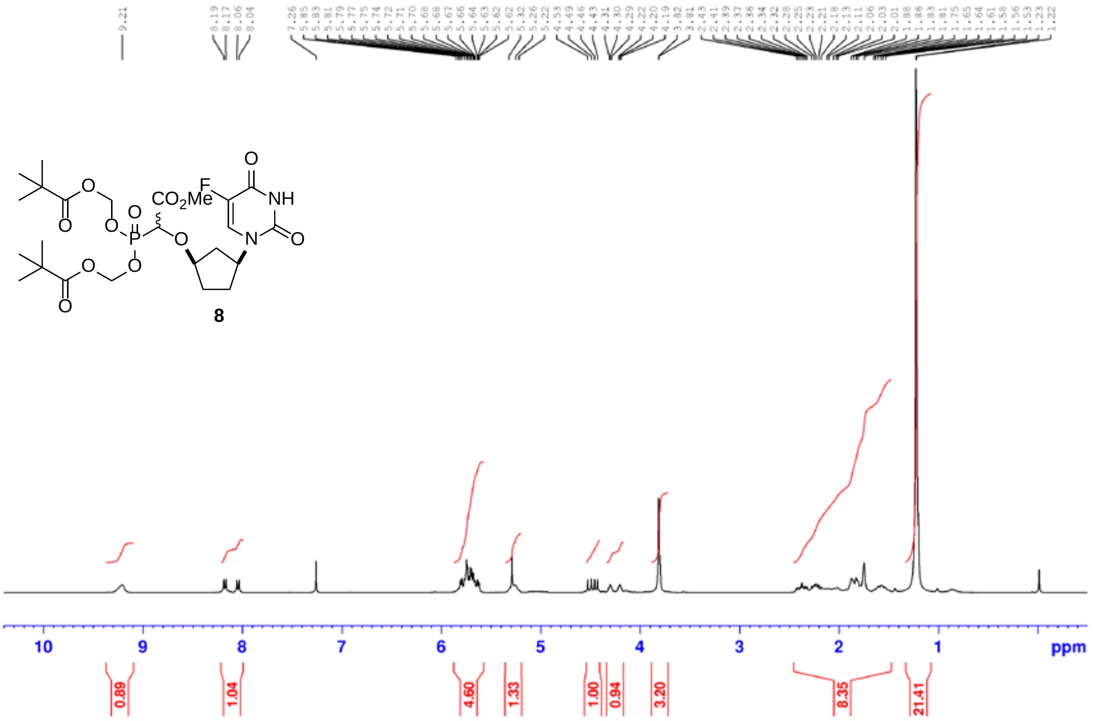

Compound **8** $^{13}C\{^{1}H\}$ NMR (75 MHz, $CDCl_3$)

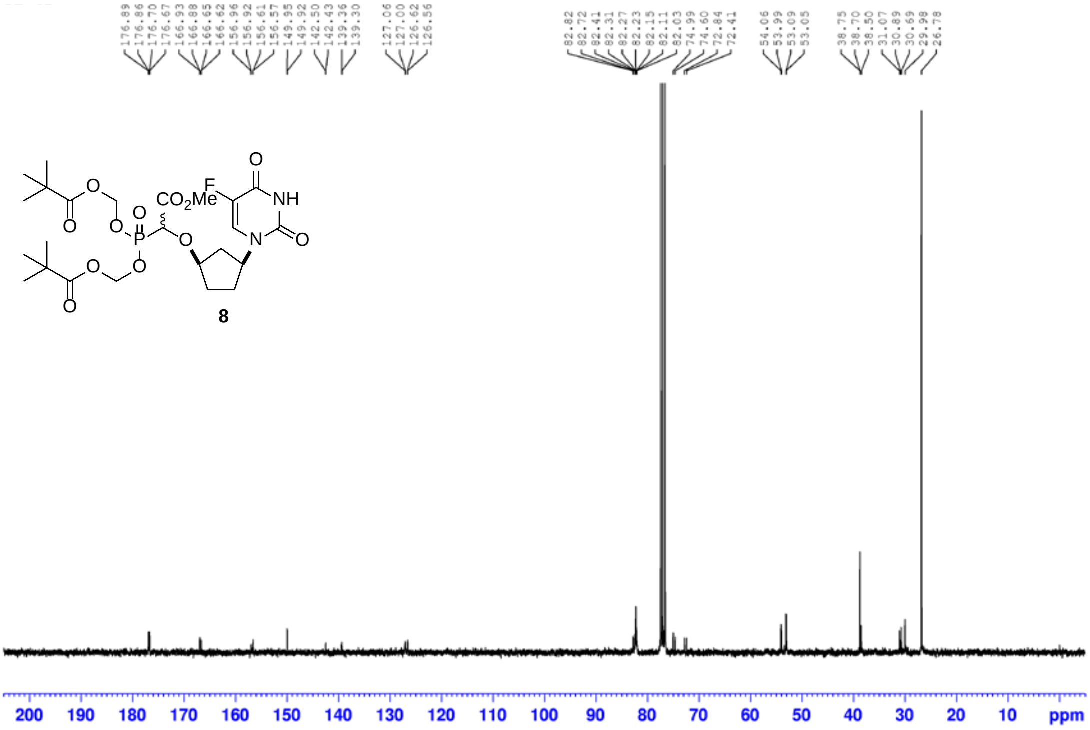

Compound **8** $^{31}P\{^{1}H\}$ NMR (121 MHz, $CDCl_3$)

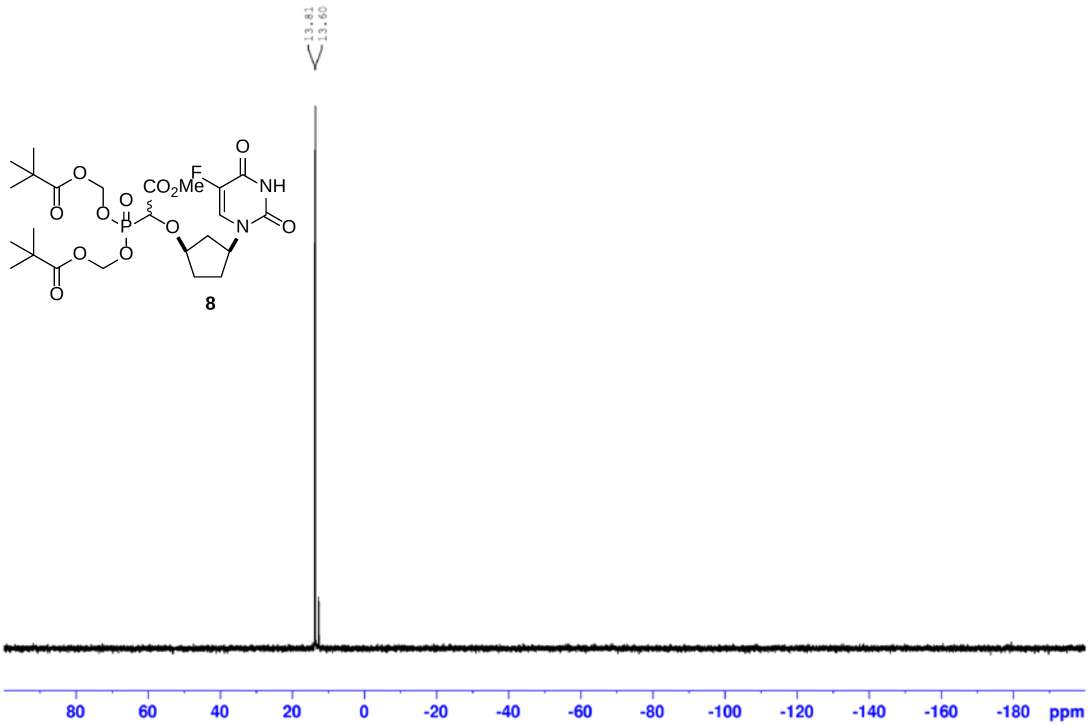

Compound **8** $^{19}F\{^1H\}$ NMR (282 MHz, $CDCl_3$)

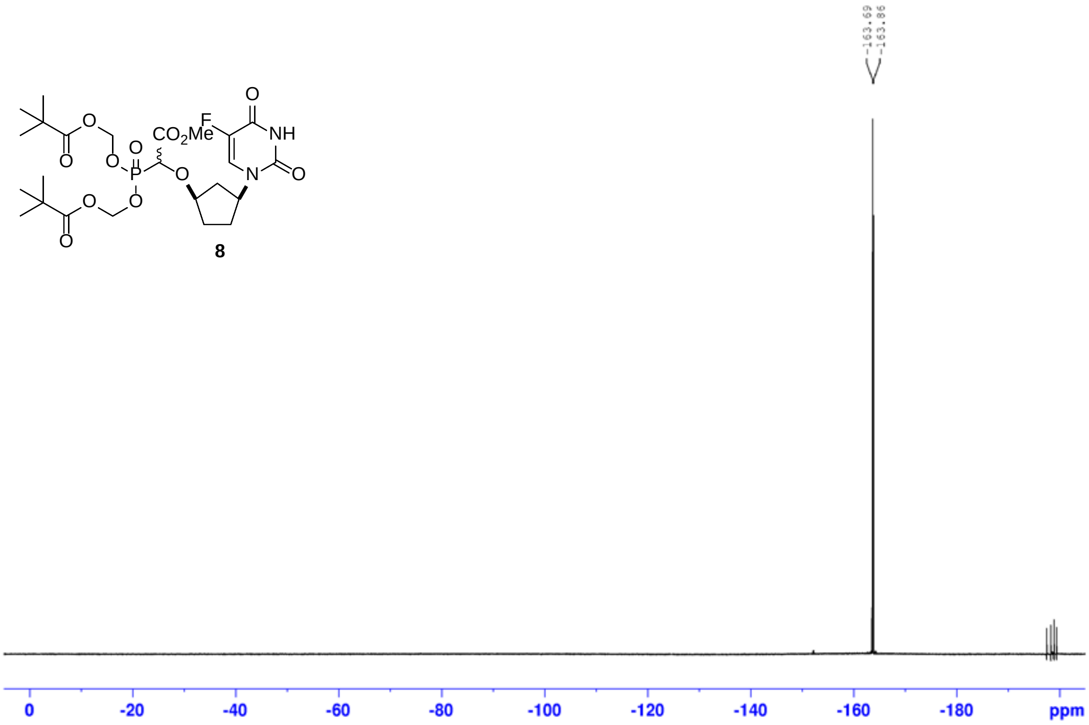

Compound **10** ^1H NMR (300 MHz, $CDCl_3$)

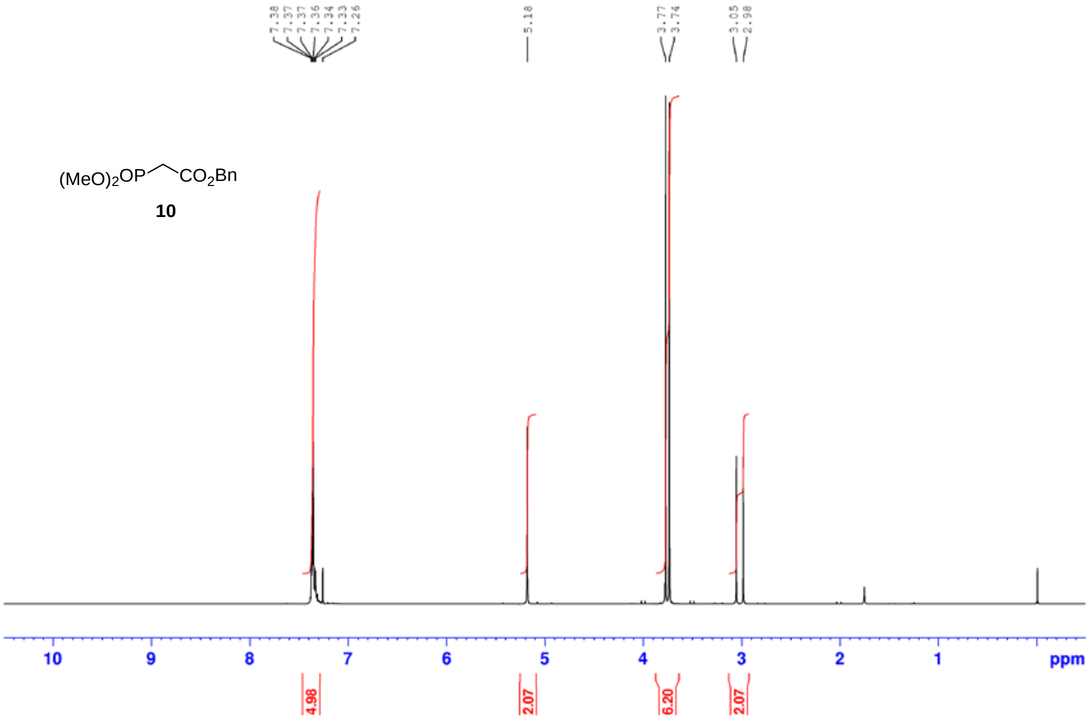

Compound **10** $^{13}C\{^1H\}$ NMR (75 MHz, $CDCl_3$)

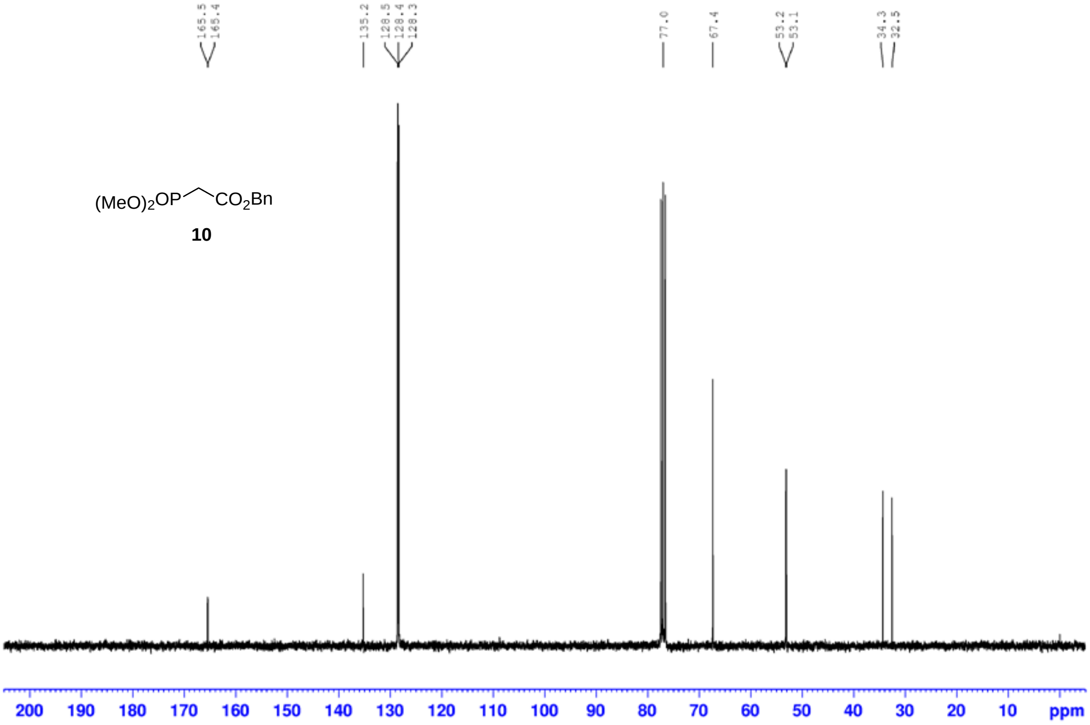

Compound **10** $^{31}P\{^1H\}$ NMR (121 MHz, $CDCl_3$)

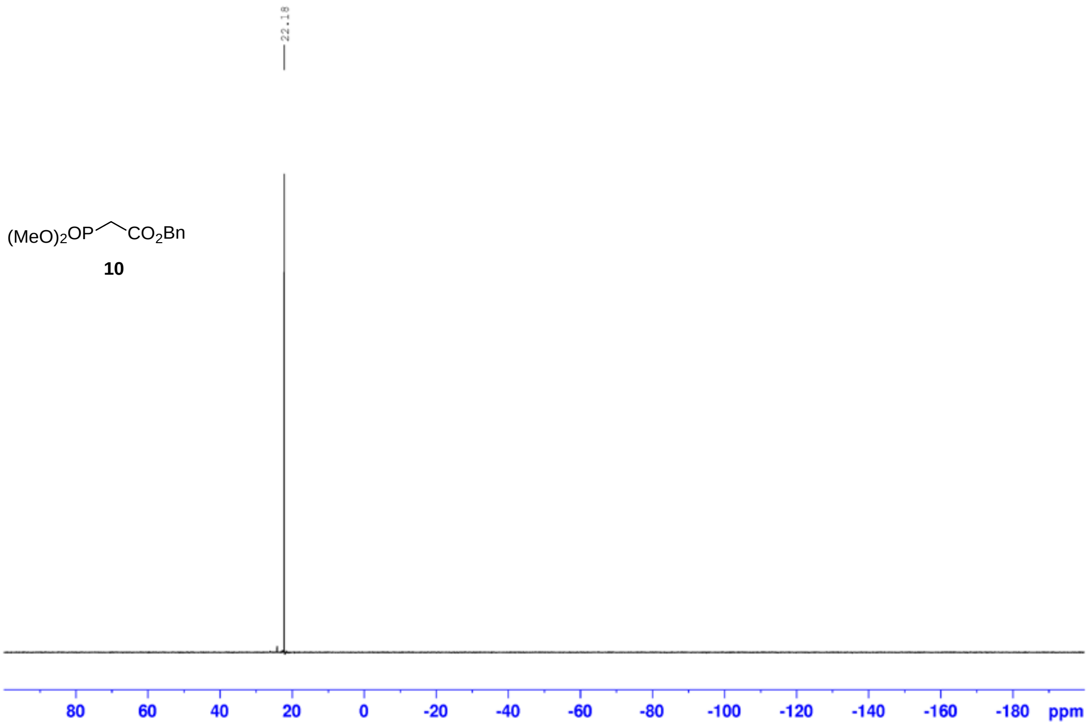

Compound **11** ^{1}H NMR (300 MHz, $CDCl_3$)

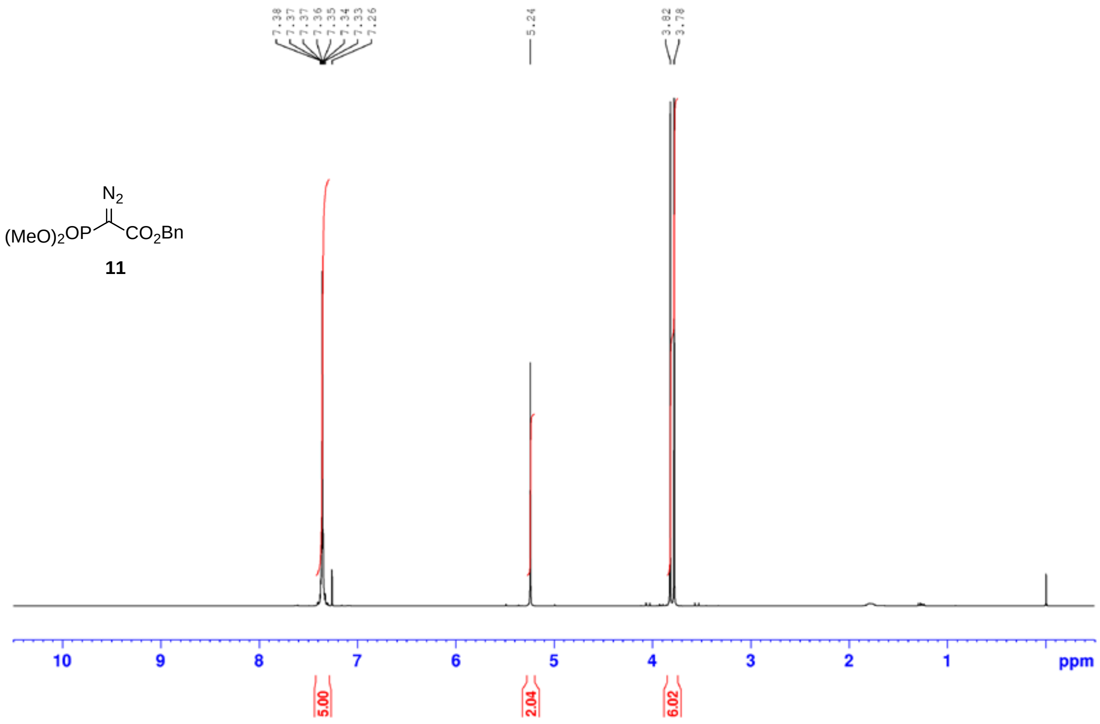

Compound **11** $^{13}C\{^{1}H\}$ NMR (75 MHz, $CDCl_3$)

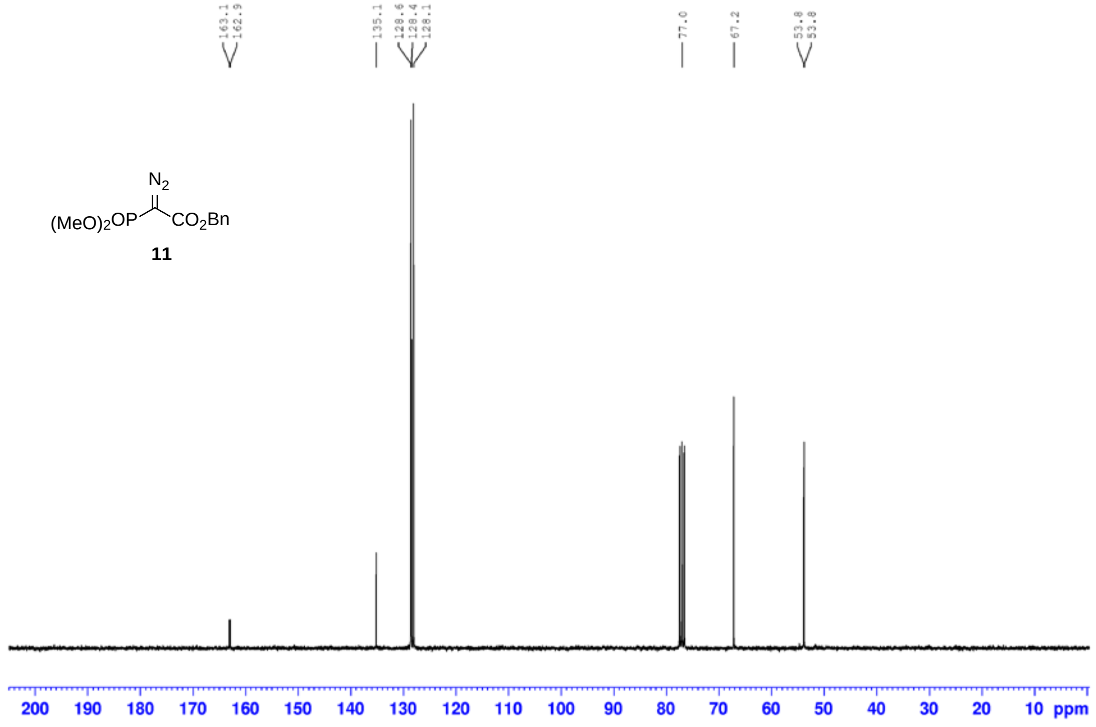

Compound **11** 31P{1H} NMR (121 MHz, $CDCl_3$)

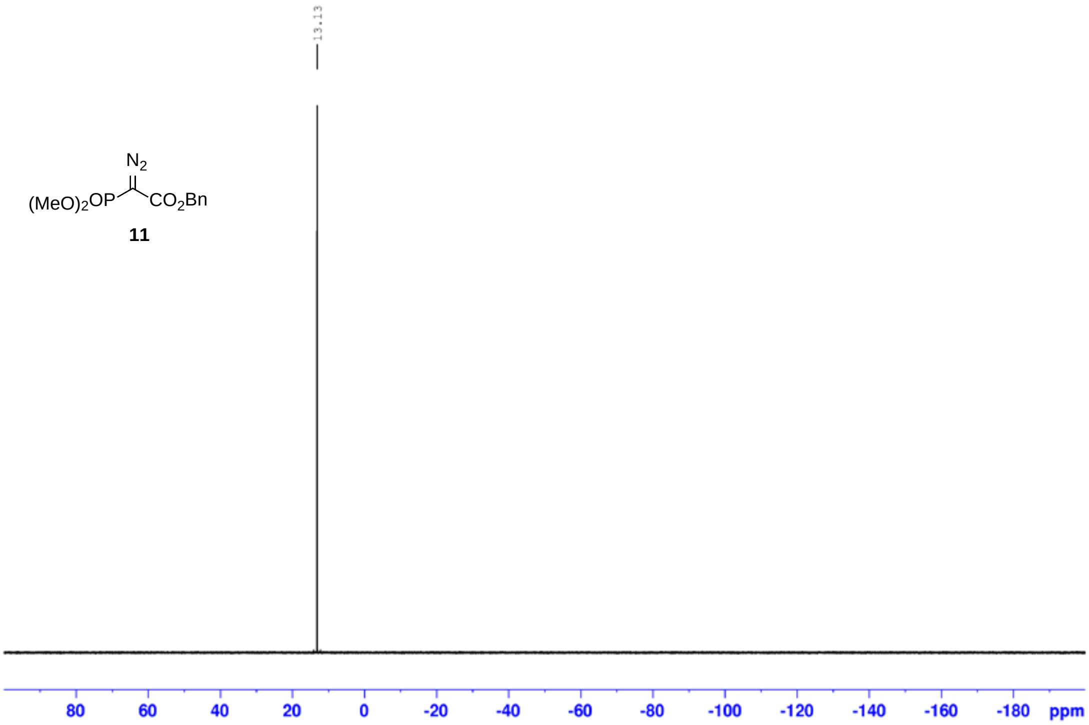

Compound **13** ^1H NMR (300 MHz, $CDCl_3$)

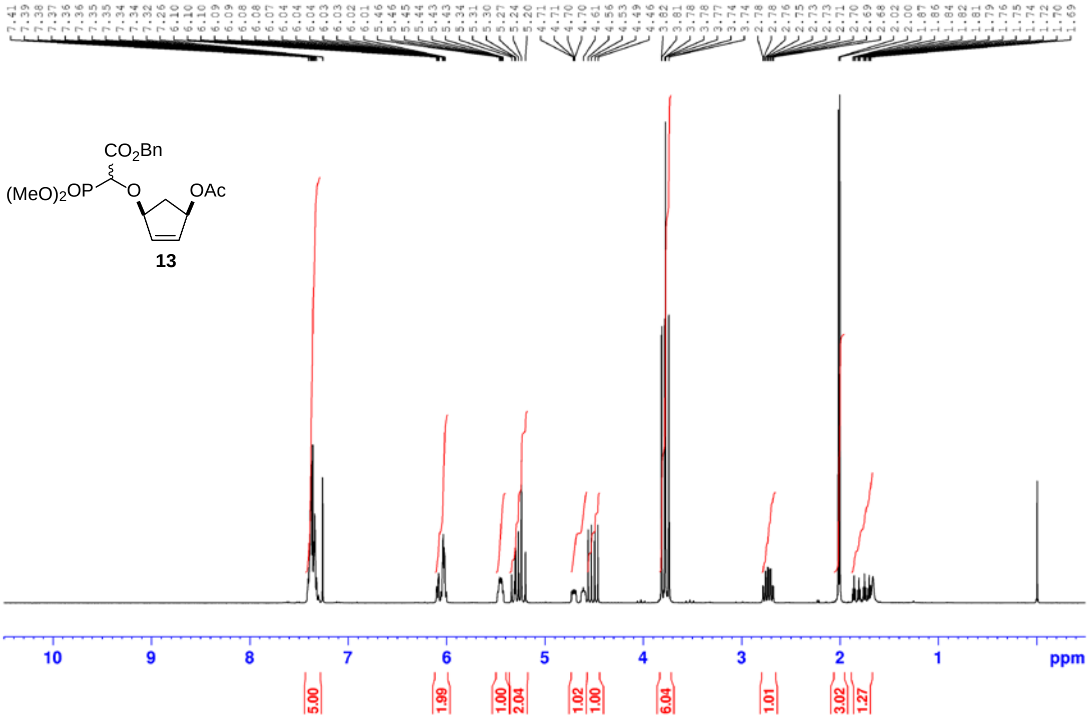

Compound **13** 13C{^{1}H} NMR (75 MHz, $CDCl_3$)

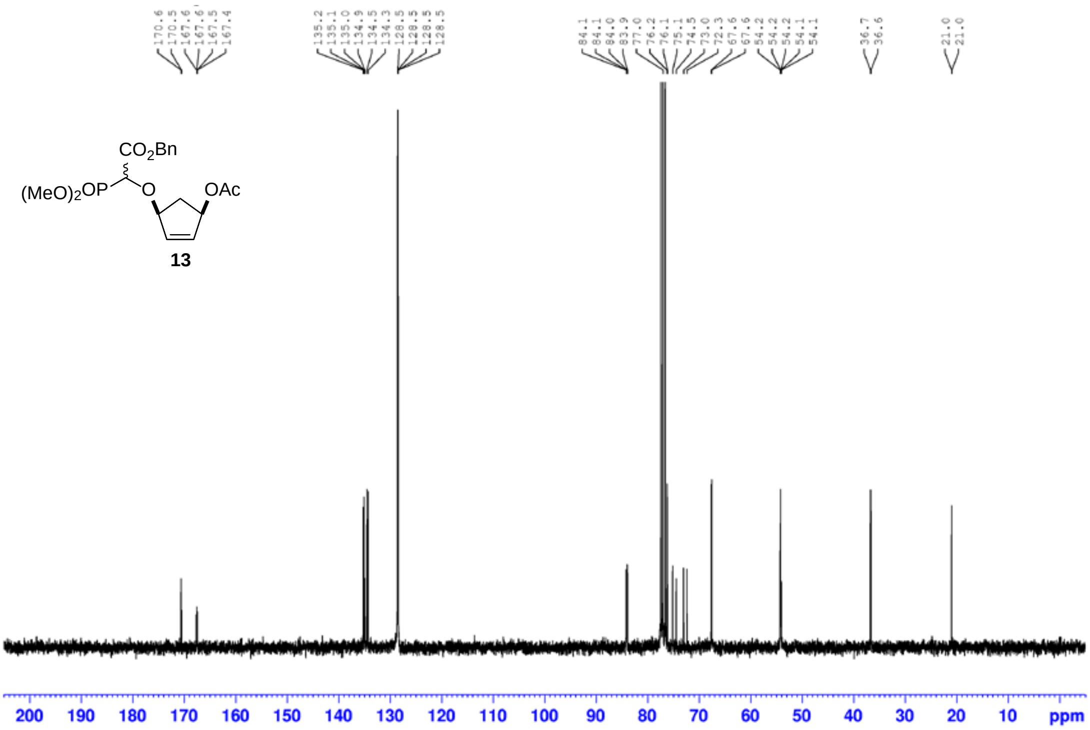

Compound **13** $^{31}P\{^{1}H\}$ NMR (121 MHz, $CDCl_3$)

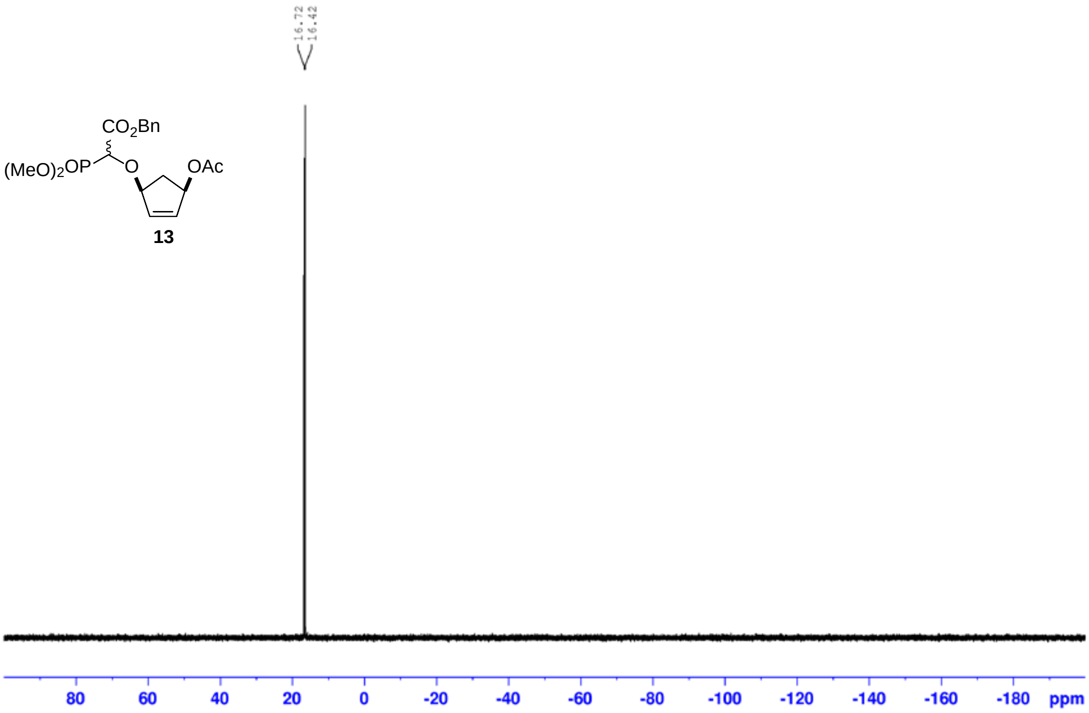

Compound **14** ^{1}H NMR (300 MHz, $CDCl_3$)

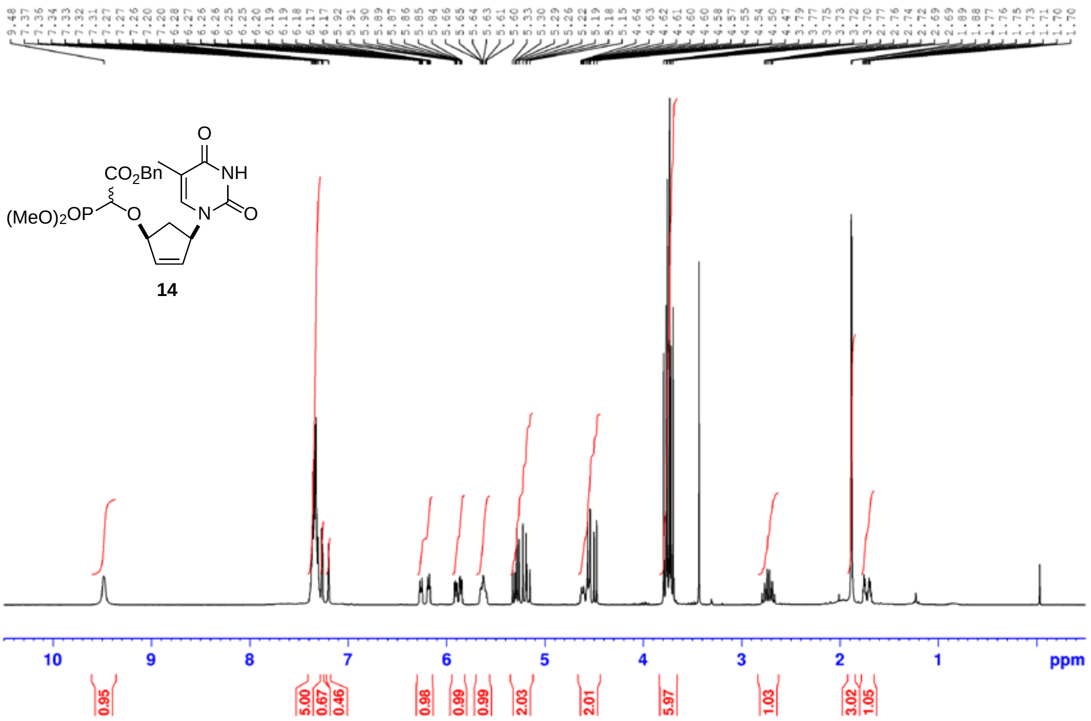

Compound **14** $^{13}C\{^{1}H\}$ NMR (75 MHz, $CDCl_3$)

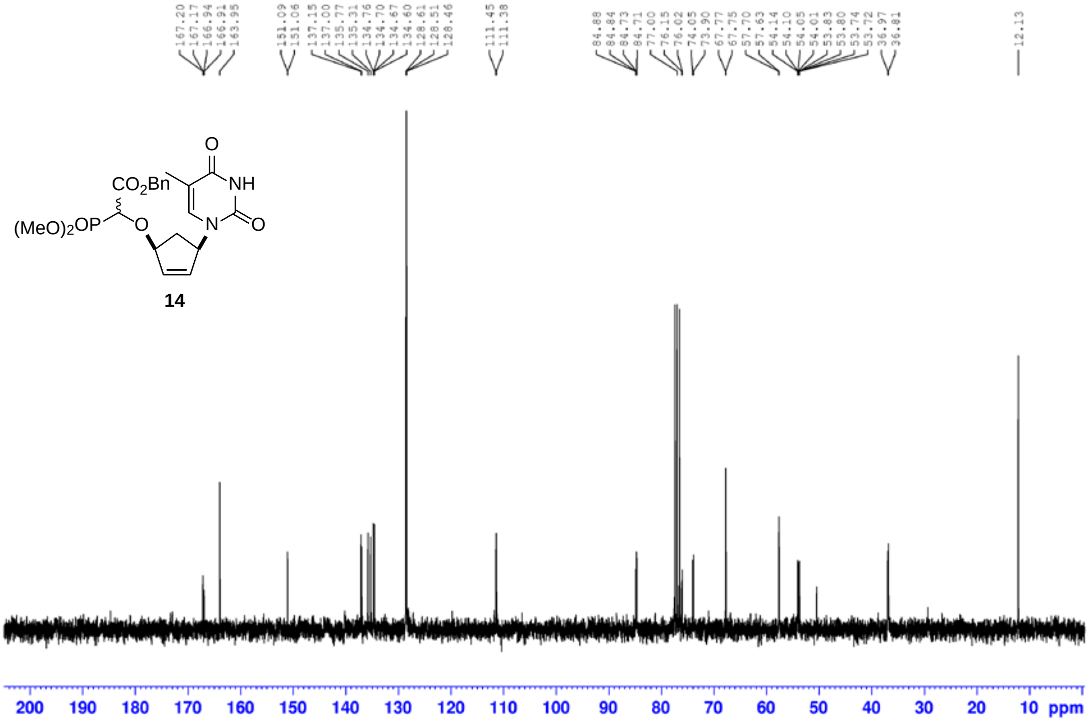

Compound **14** $^{31}P\{^{1}H\}$ NMR (121 MHz, $CDCl_3$)

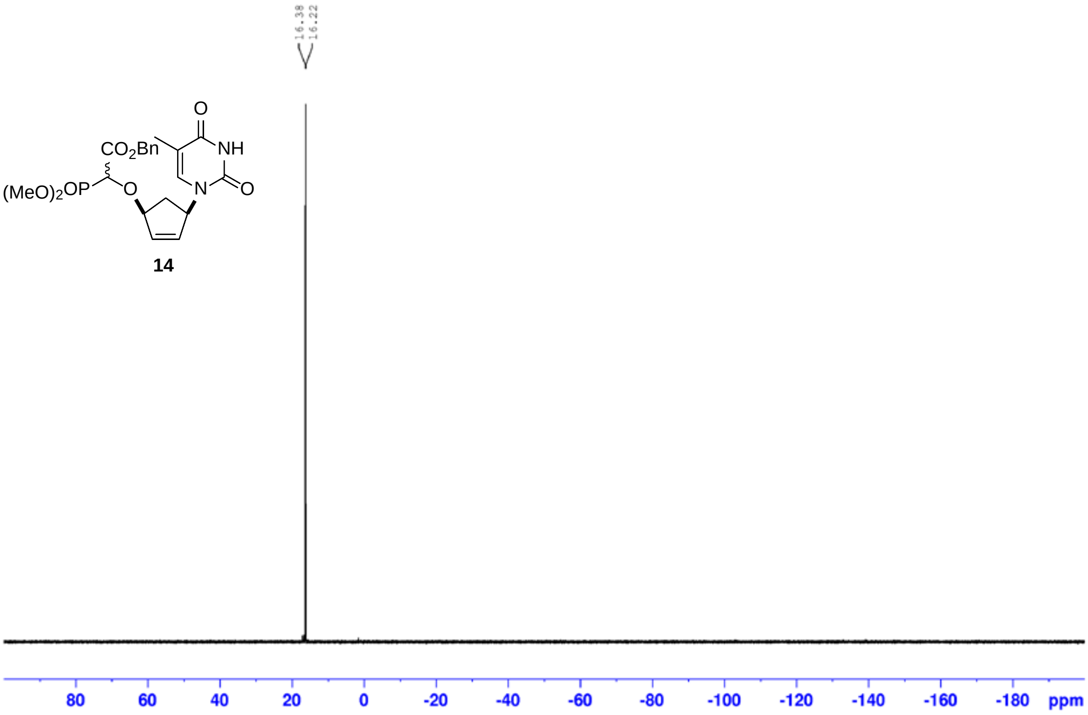

Compound **15** ^{1}H NMR (300 MHz, $CDCl_3$)

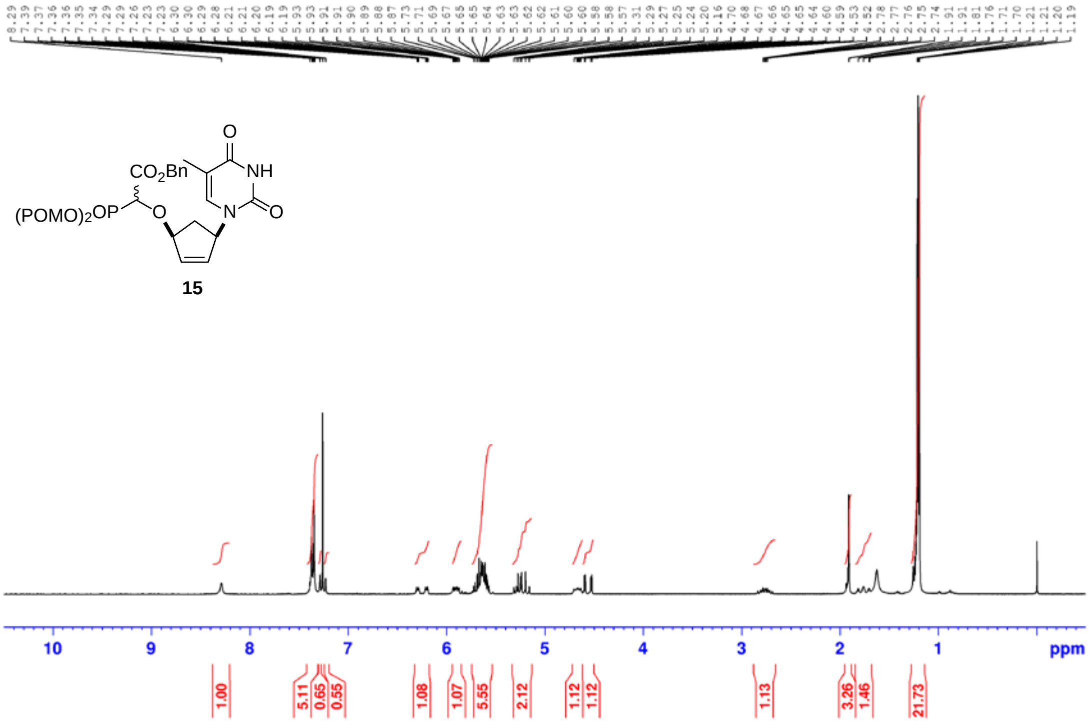

Compound **15** $^{13}C\{^1H\}$ NMR (75 MHz, $CDCl_3$)

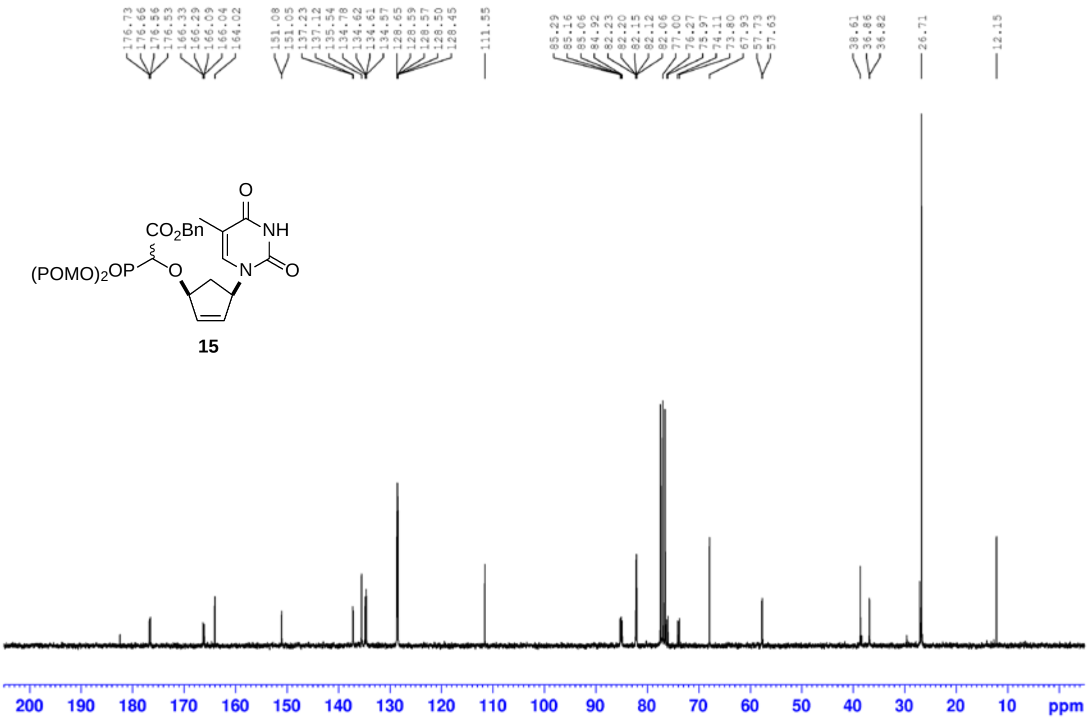

Compound **15** $^{31}P\{^1H\}$ NMR (121 MHz, $CDCl_3$)

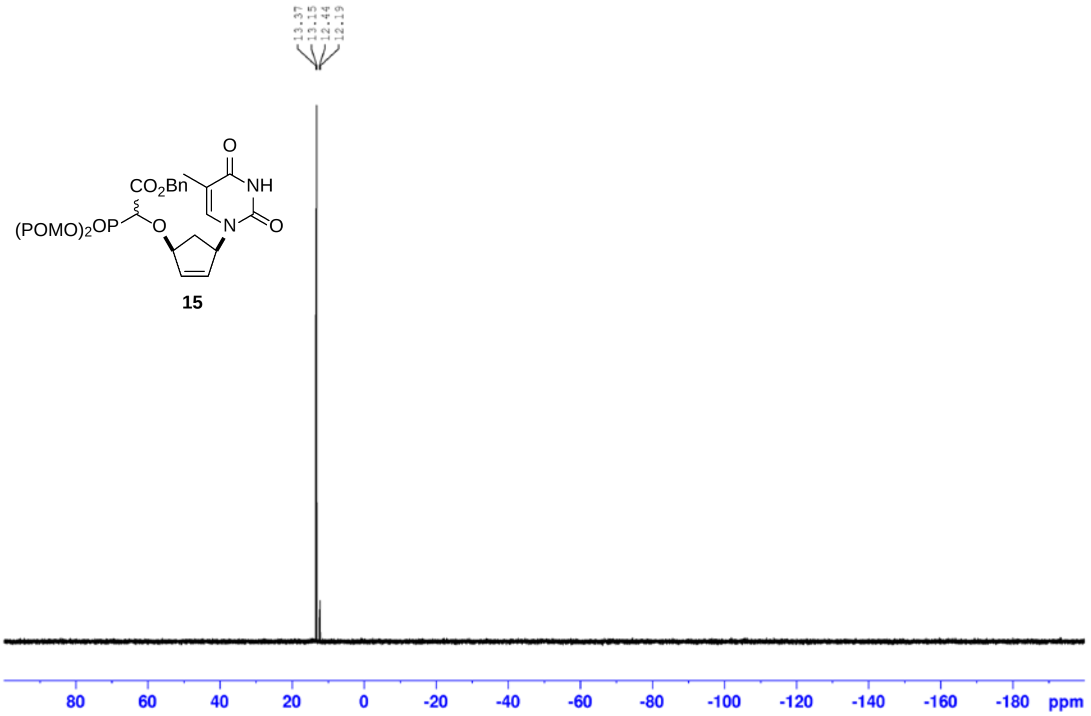

Compound **3** ^{1}H NMR (300 MHz, $CDCl_3$)

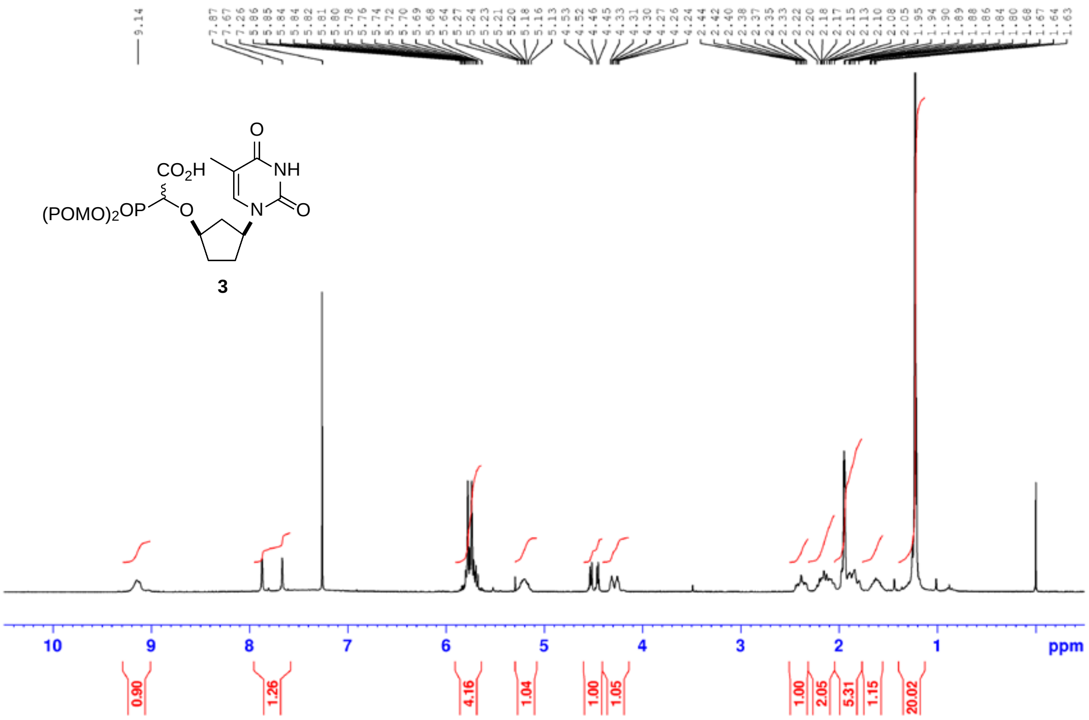

Compound **3** $^{13}C\{^{1}H\}$ NMR (300 MHz, $CDCl_3$)

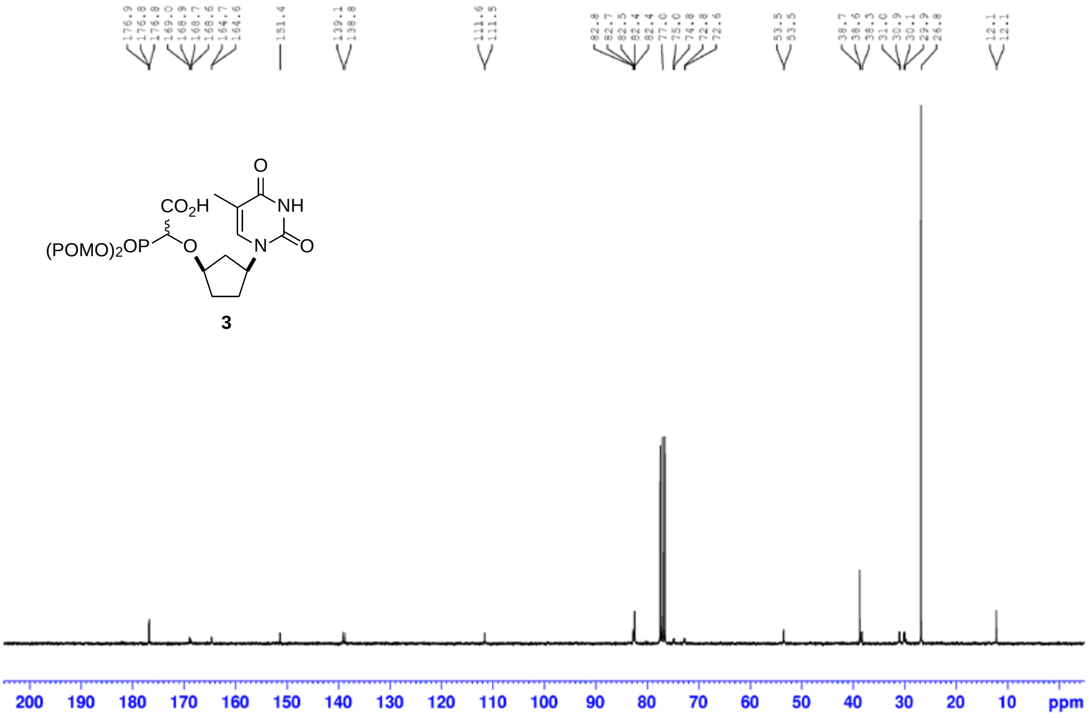

Compound **3** $^{31}P\{^{1}H\}$ NMR (300 MHz, $CDCl_3$)

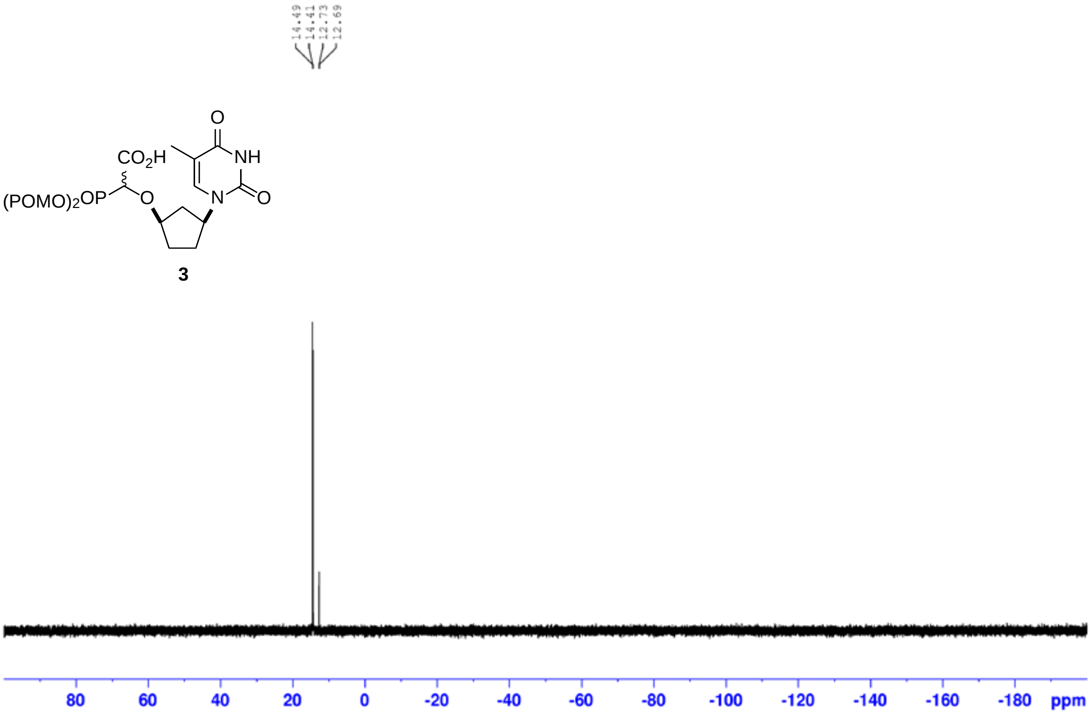

Compound **16** ^{1}H NMR (300 MHz, $CDCl_3$)

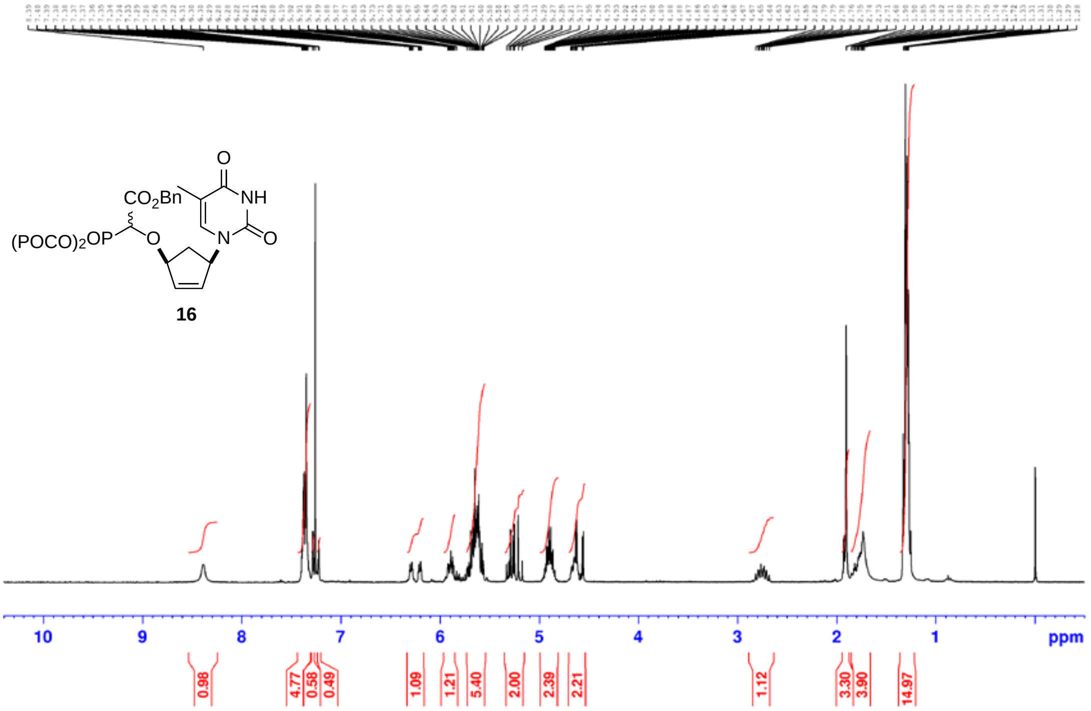

Compound **16** $^{13}C\{^{1}H\}$ NMR (75 MHz, $CDCl_3$)

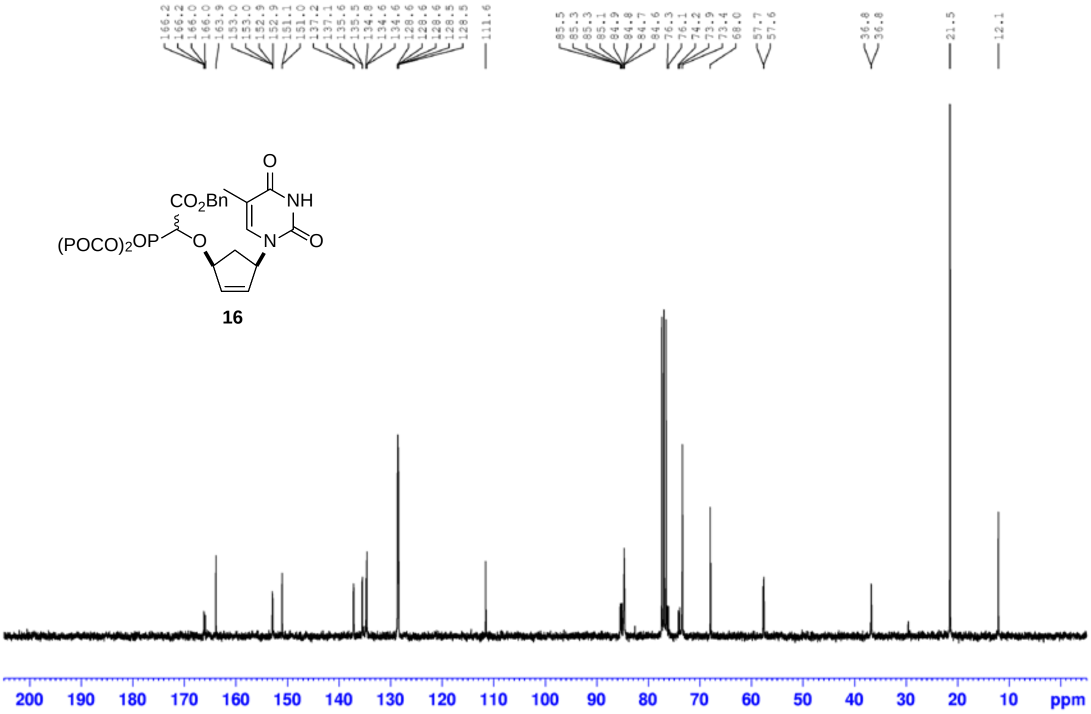

Compound **16** $^{31}P\{^{1}H\}$ NMR (121 MHz, $CDCl_3$)

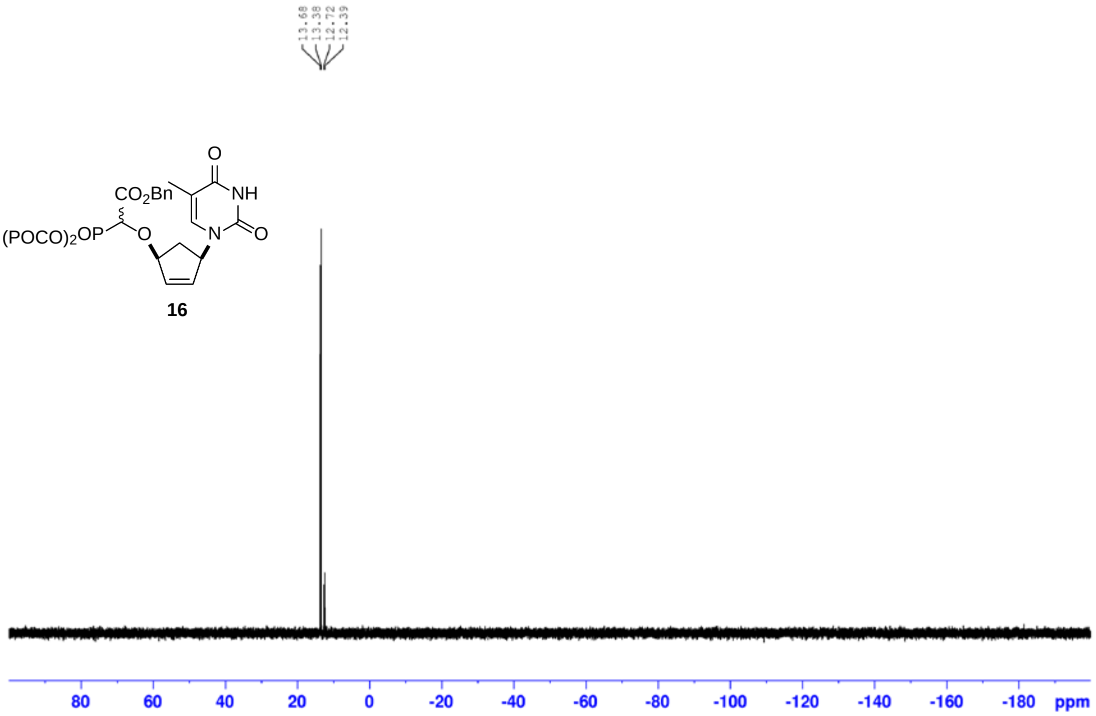

Compound **19** ^1H NMR (300 MHz, $CDCl_3$)

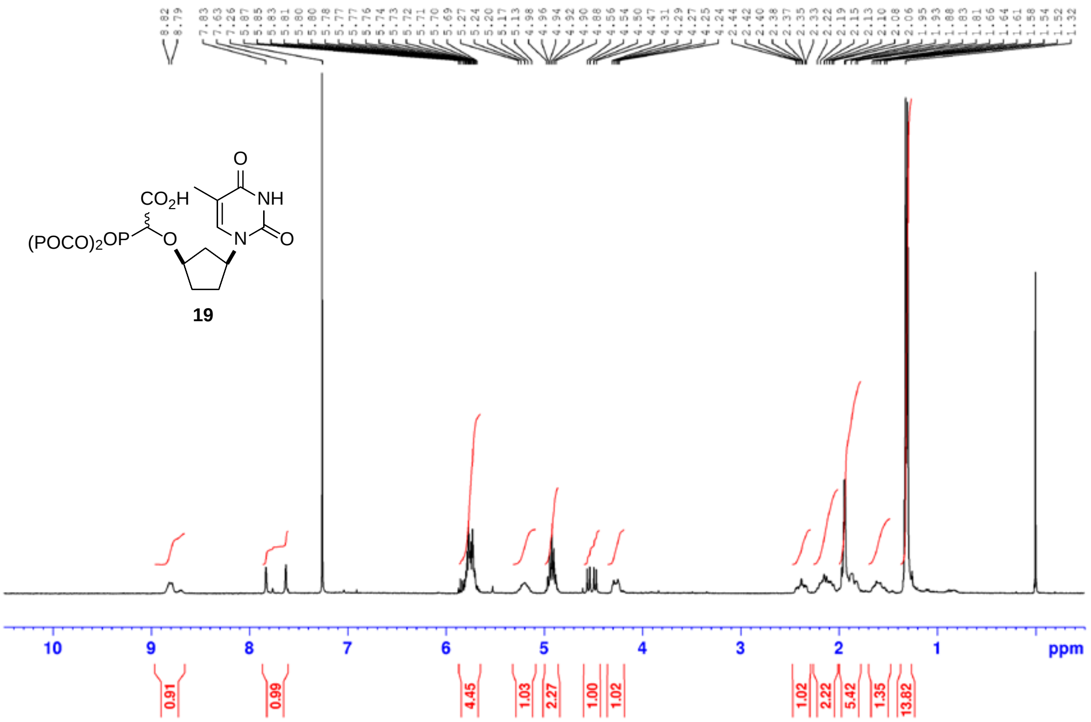

Compound **19** $^{13}C\{^1H\}$ NMR (75 MHz, $CDCl_3$)

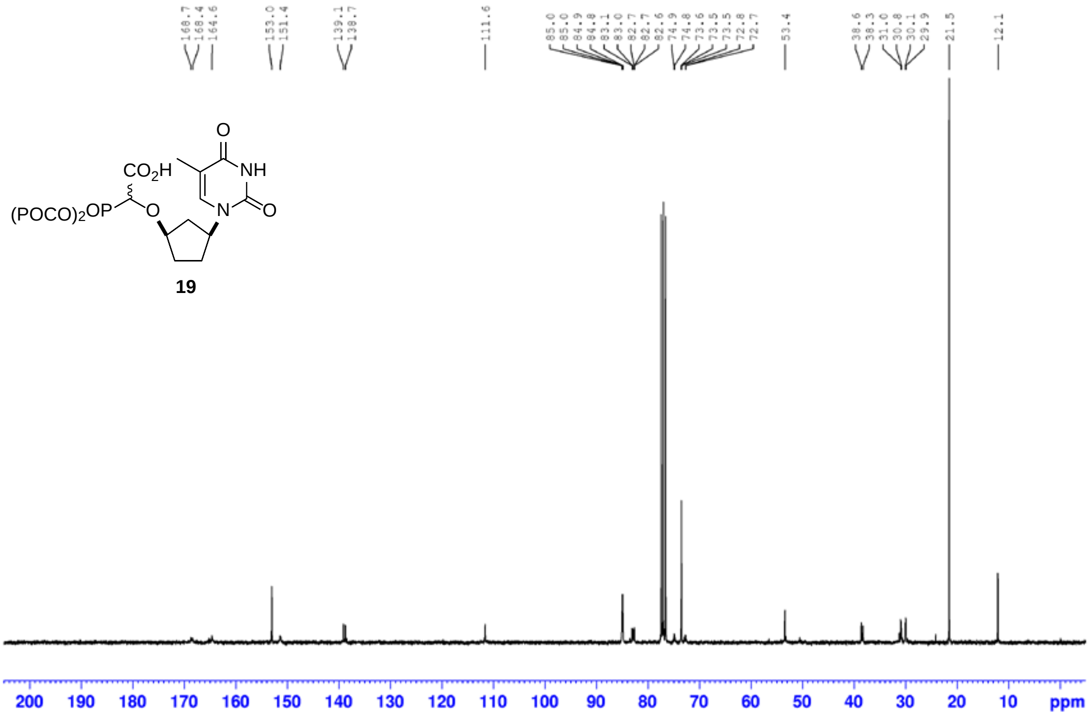

Compound **19** $^{31}P\{^{1}H\}$ NMR (121 MHz, $CDCl_3$)

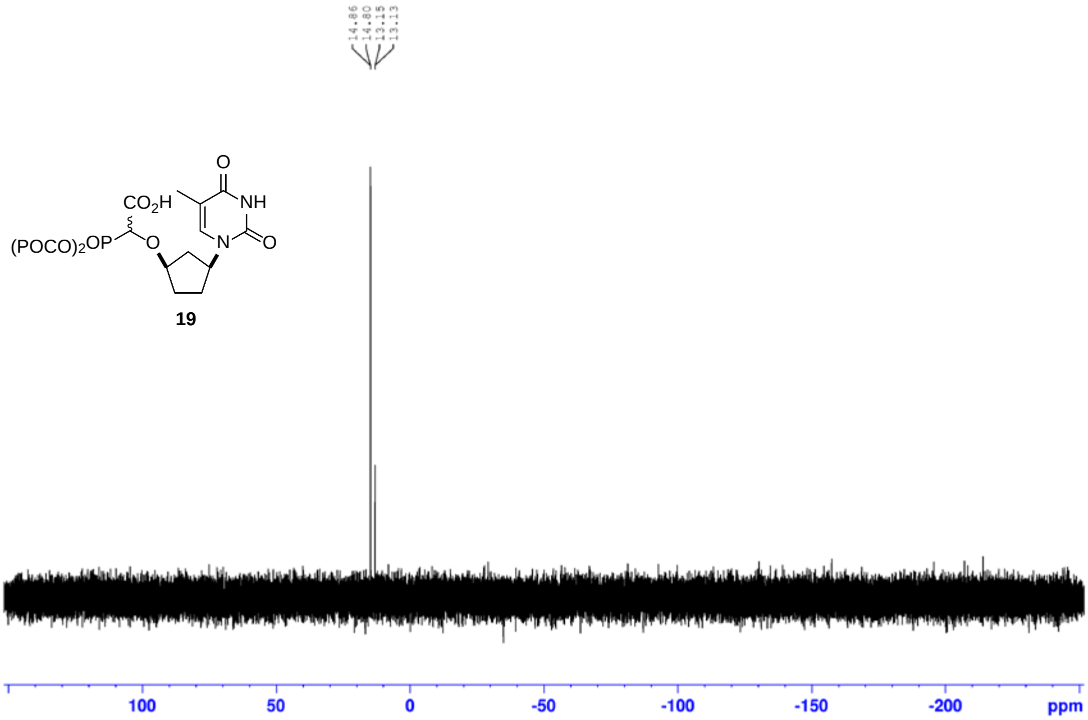

Compound **17** ^{1}H NMR (300 MHz, $CDCl_3$)

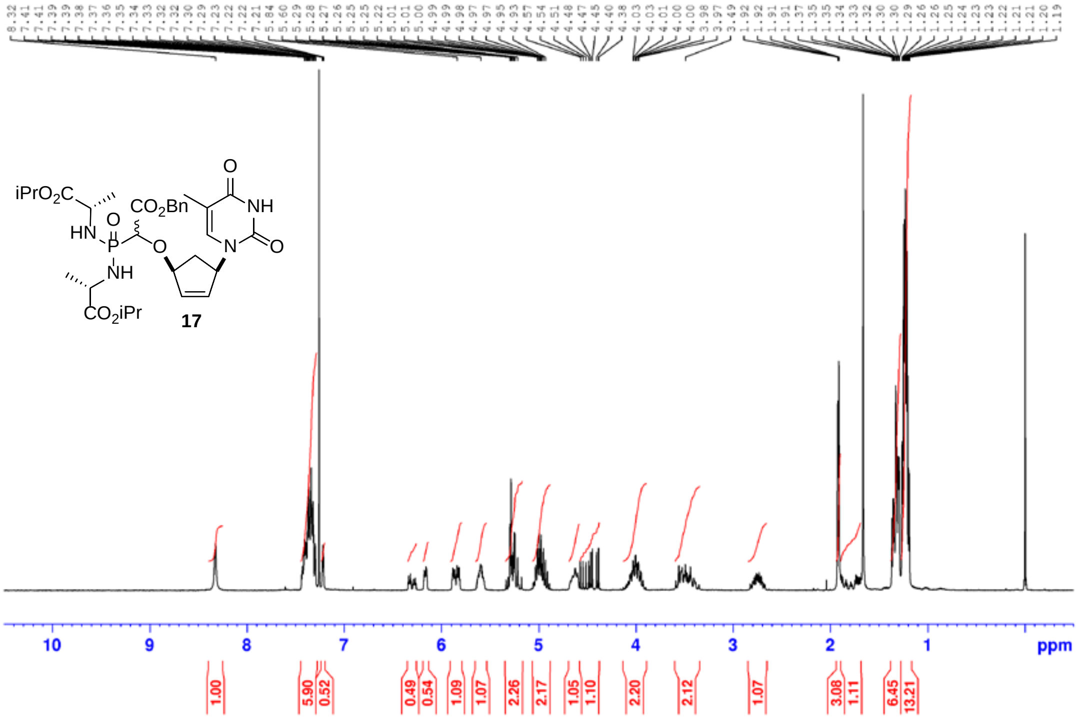

Compound **17** $^{31}P\{^{1}H\}$ NMR (121 MHz, $CDCl_3$)

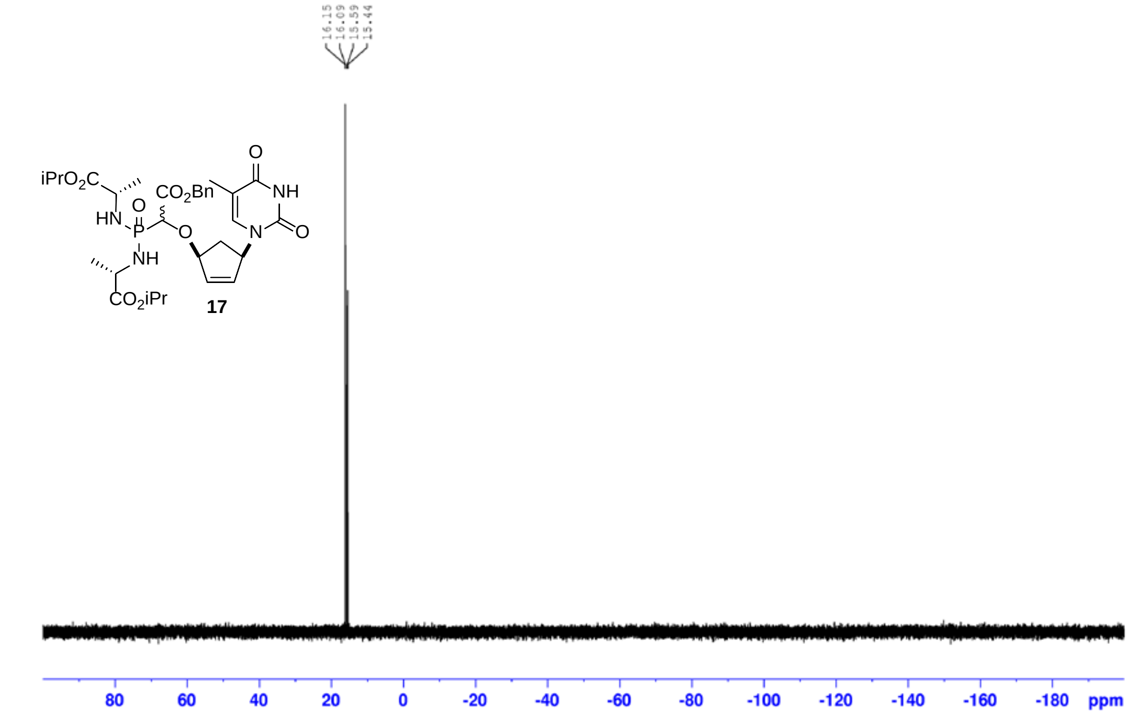

Compound **20** ^{1}H NMR (600 MHz, $CDCl_3$)

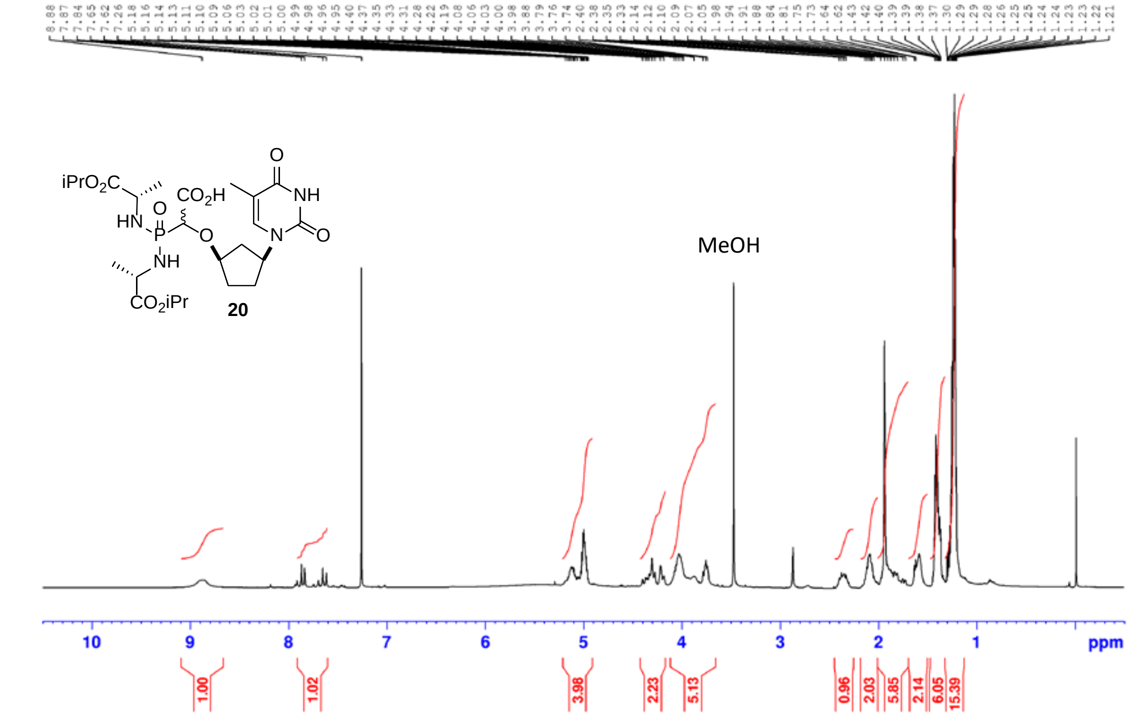

Compound **20** $^{13}C\{^{1}H\}$ NMR (150 MHz, $CDCl_3$)

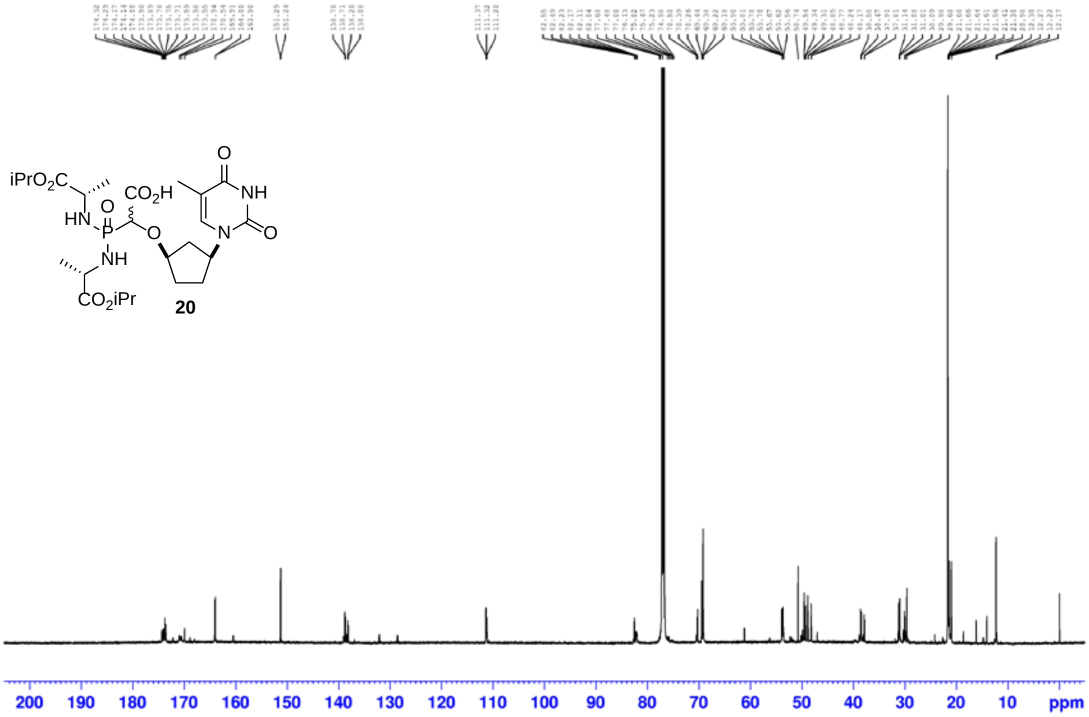

Compound **20** $^{31}P\{^{1}H\}$ NMR (121 MHz, $CDCl_3$)

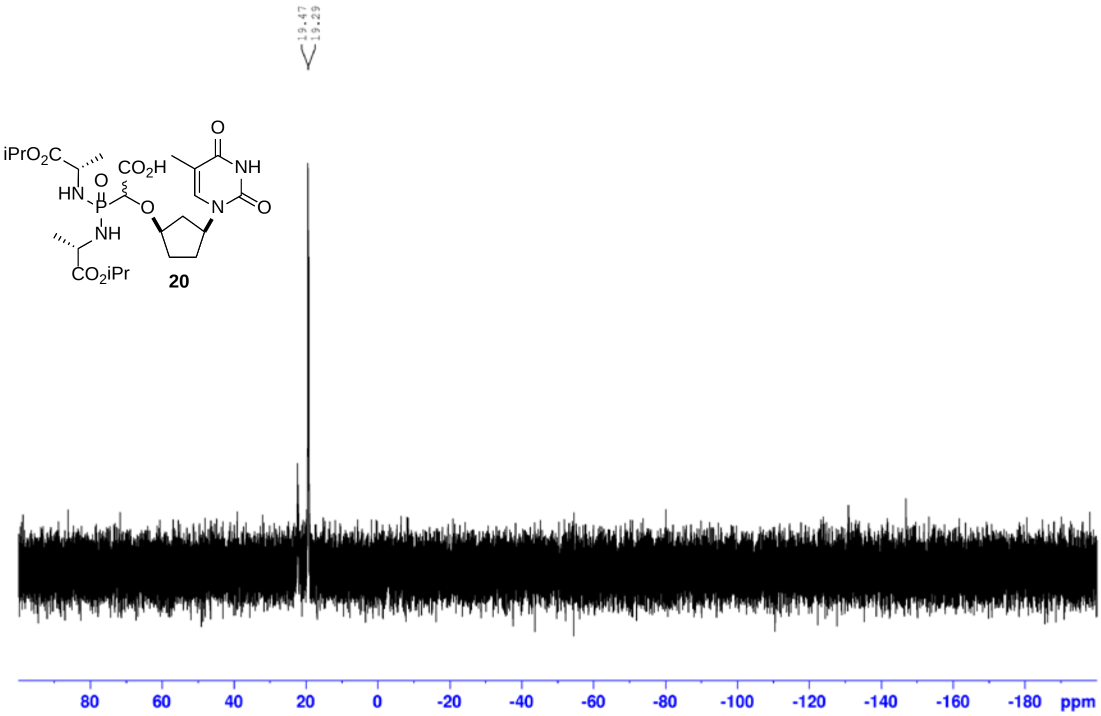

Compound **18** ^{1}H NMR (300 MHz, $CDCl_3$)

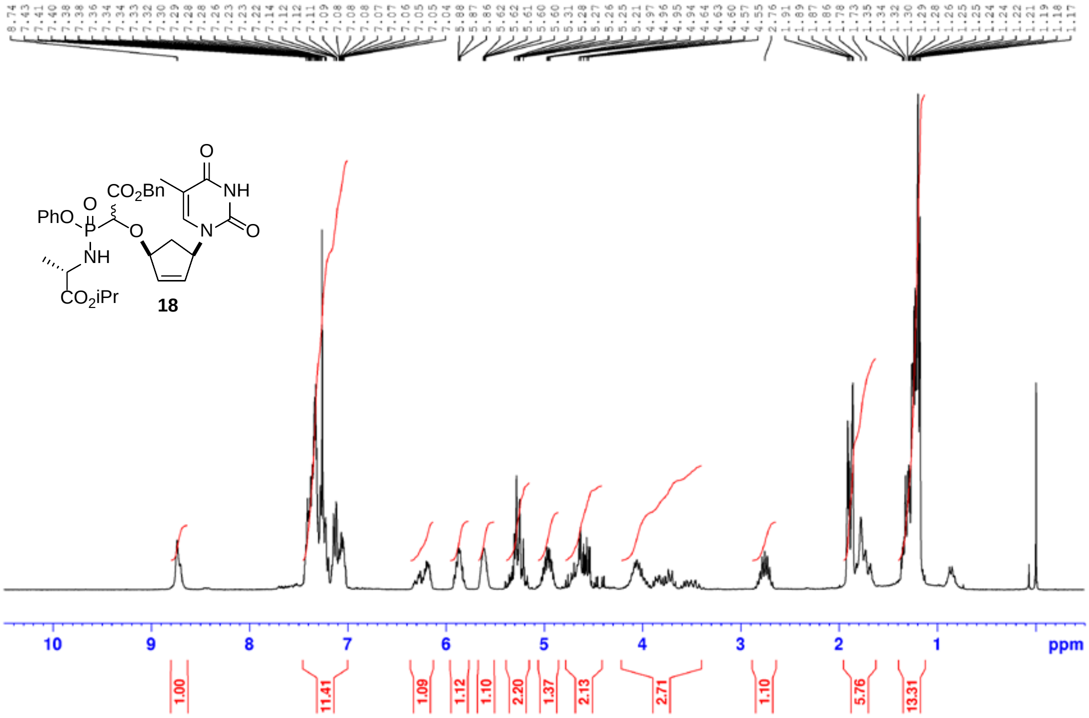

Compound **18** $^{31}P\{^{1}H\}$ NMR (121 MHz, $CDCl_3$)

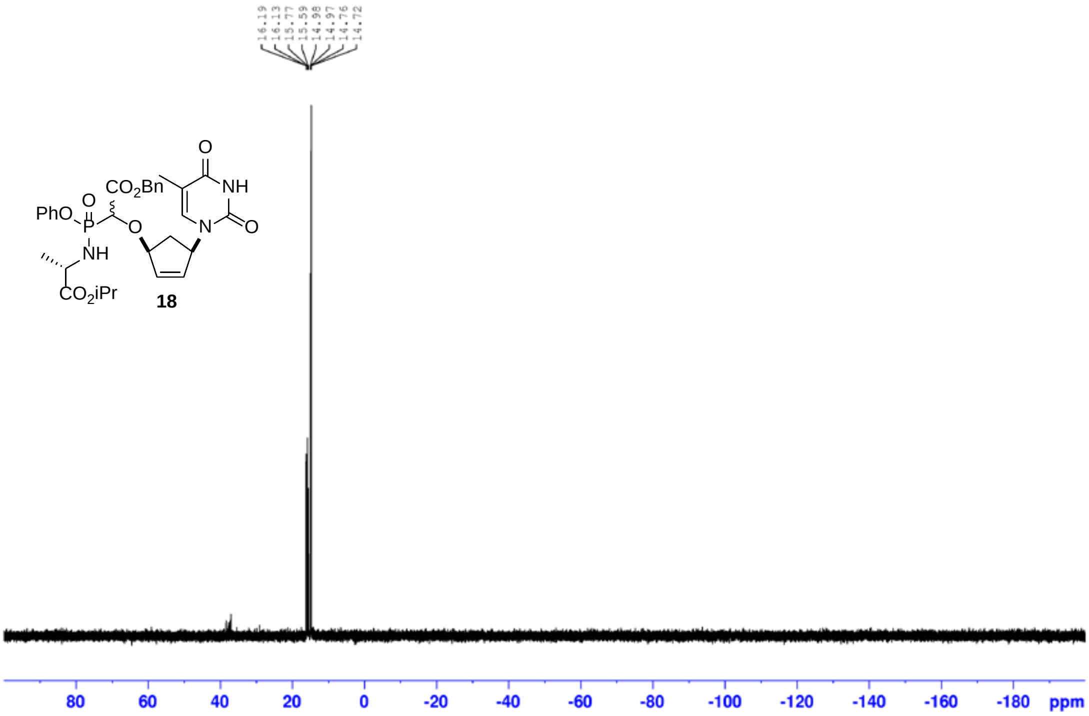

Compound **21** ^{1}H NMR (600 MHz, $CDCl_3$)

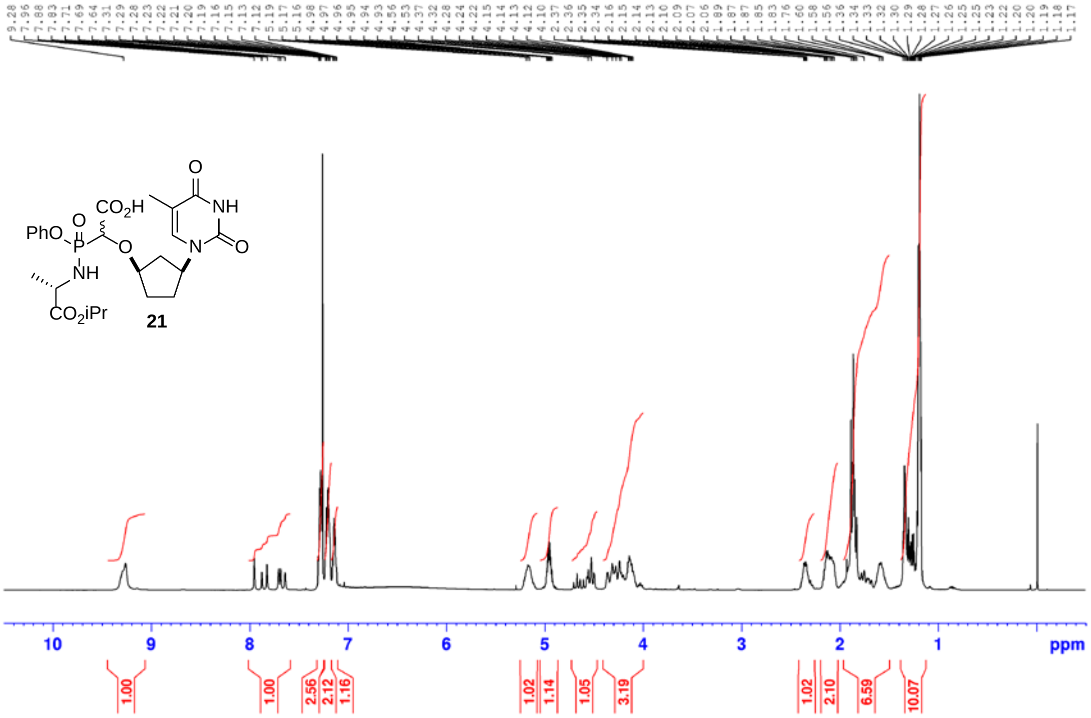

Compound **21** $^{13}C\{^{1}H\}$ NMR (150 MHz, $CDCl_3$)

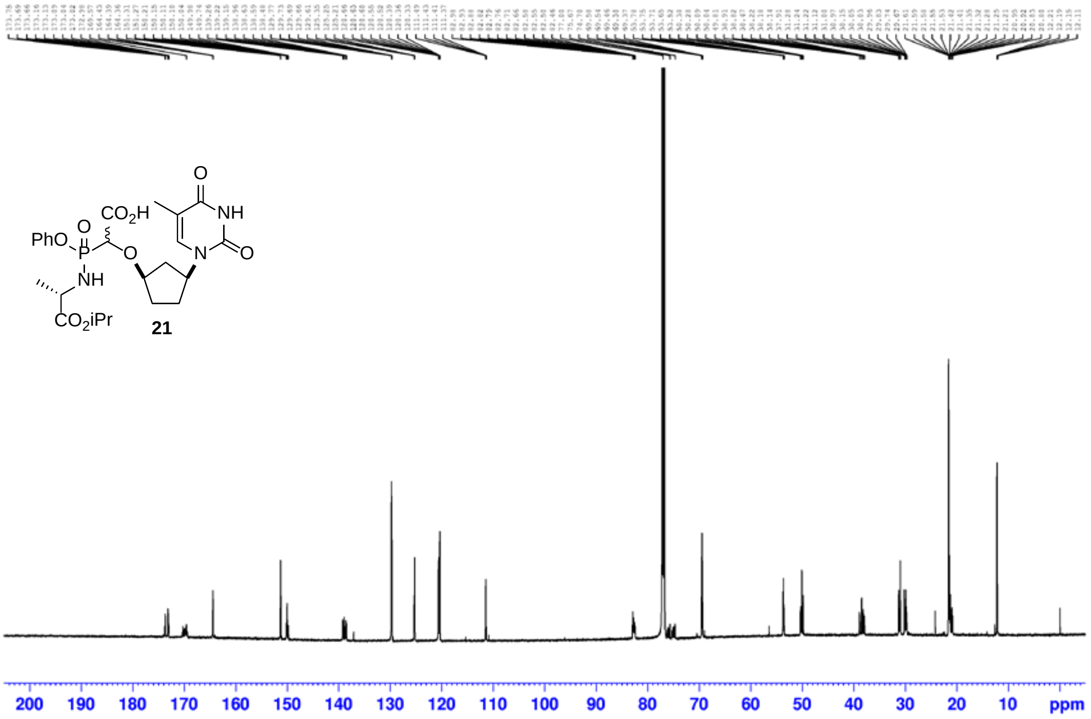

Compound **10** $^{31}P\{^1H\}$ NMR (121 MHz, $CDCl_3$)

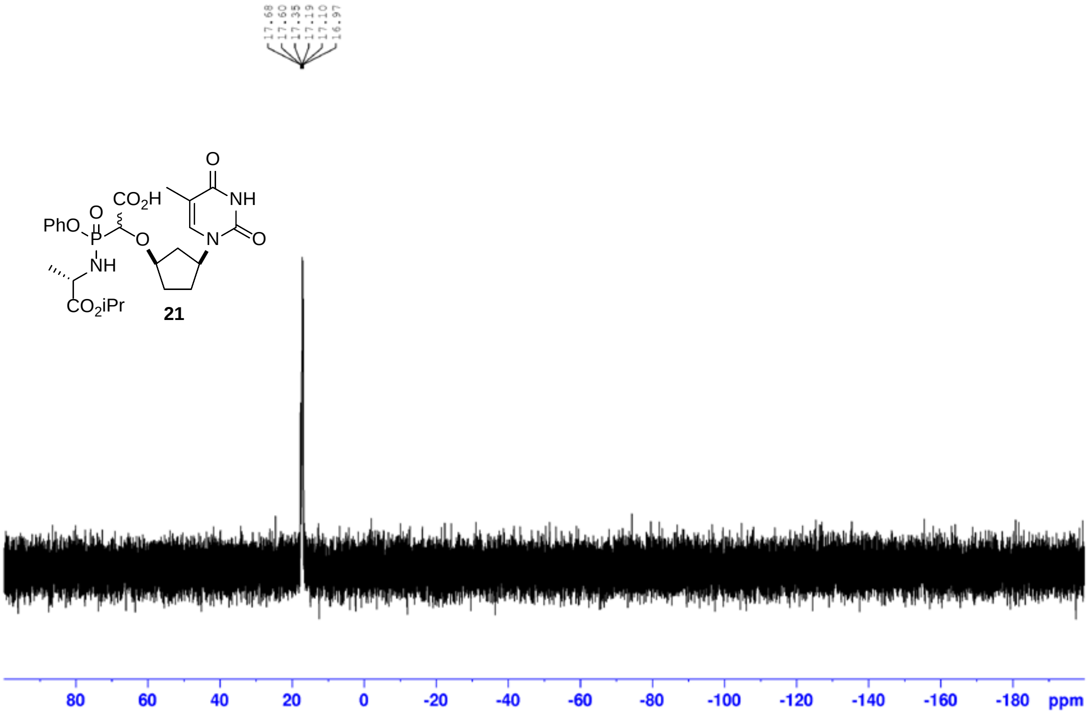

Compound **24** ^1H NMR (300 MHz, $CDCl_3$)

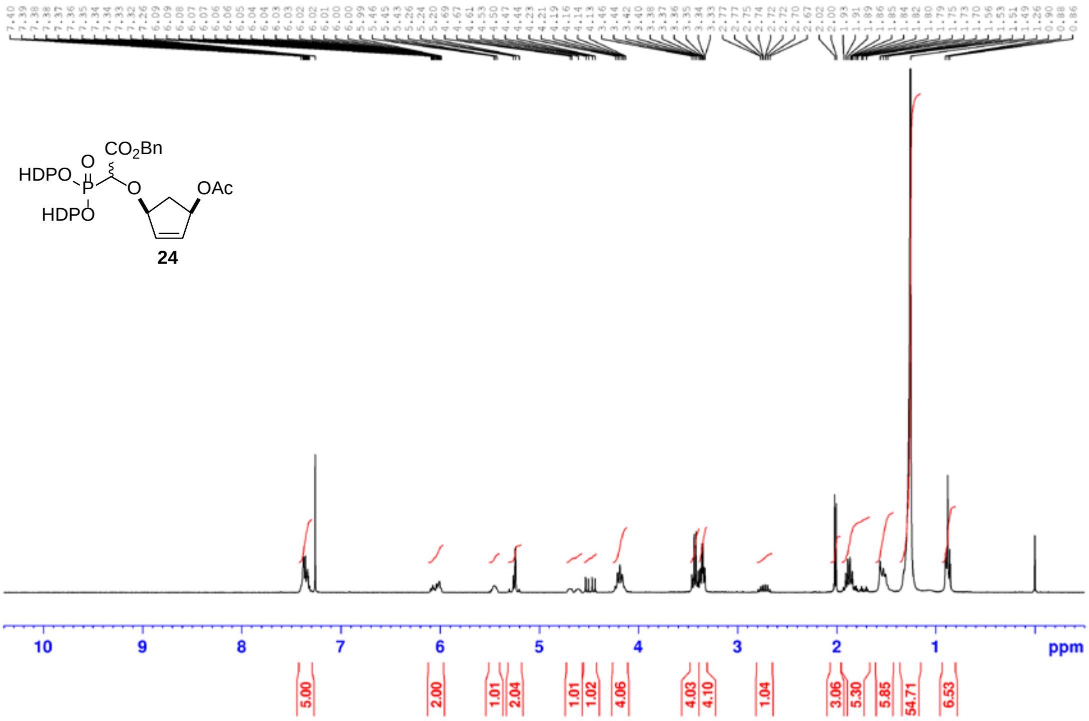

Compound **24** $^{13}C\{^{1}H\}$ NMR (75 MHz, $CDCl_3$)

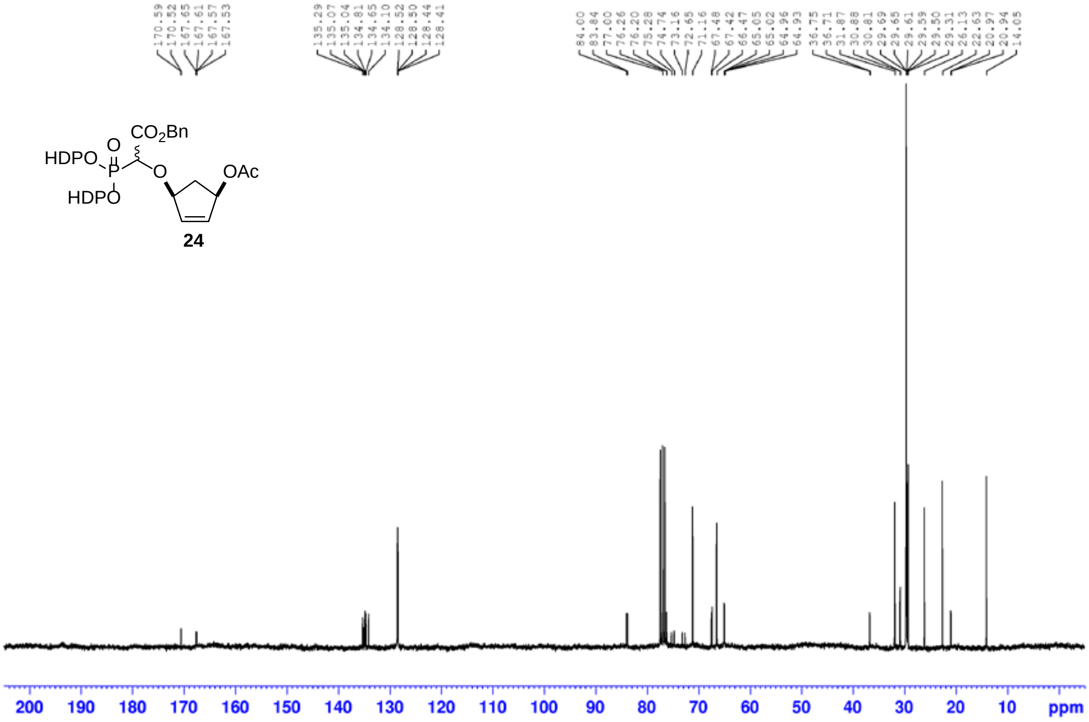

Compound **24** $^{31}P\{^{1}H\}$ NMR (121 MHz, $CDCl_3$)

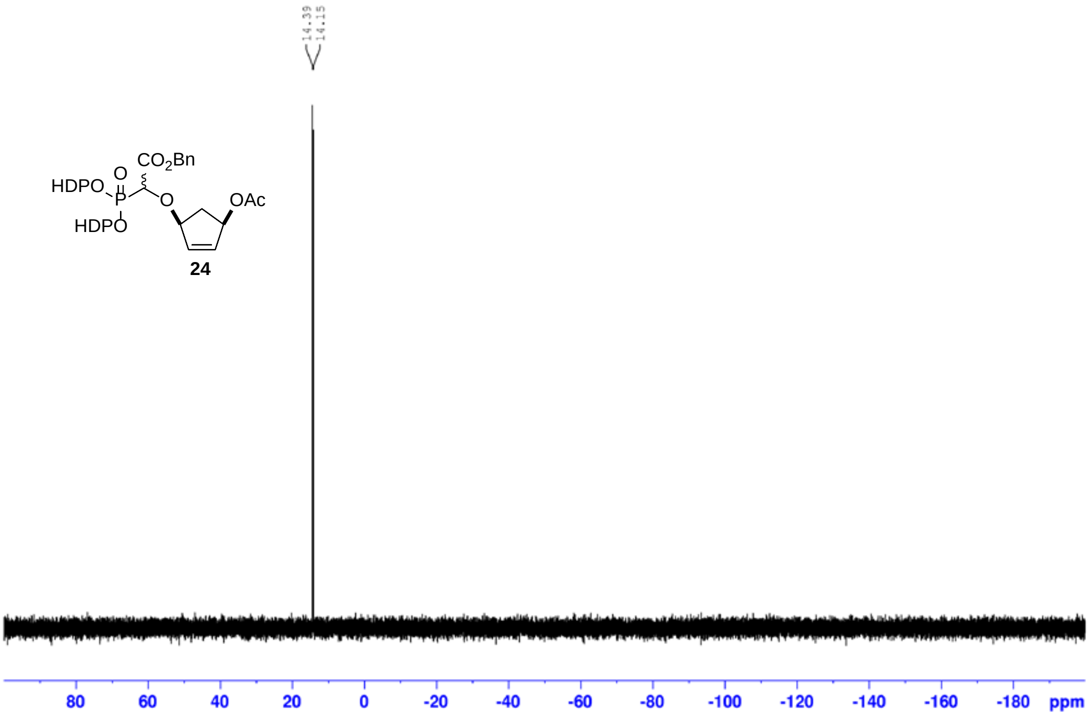

Compound **25** ^{1}H NMR (300 MHz, $CDCl_3$)

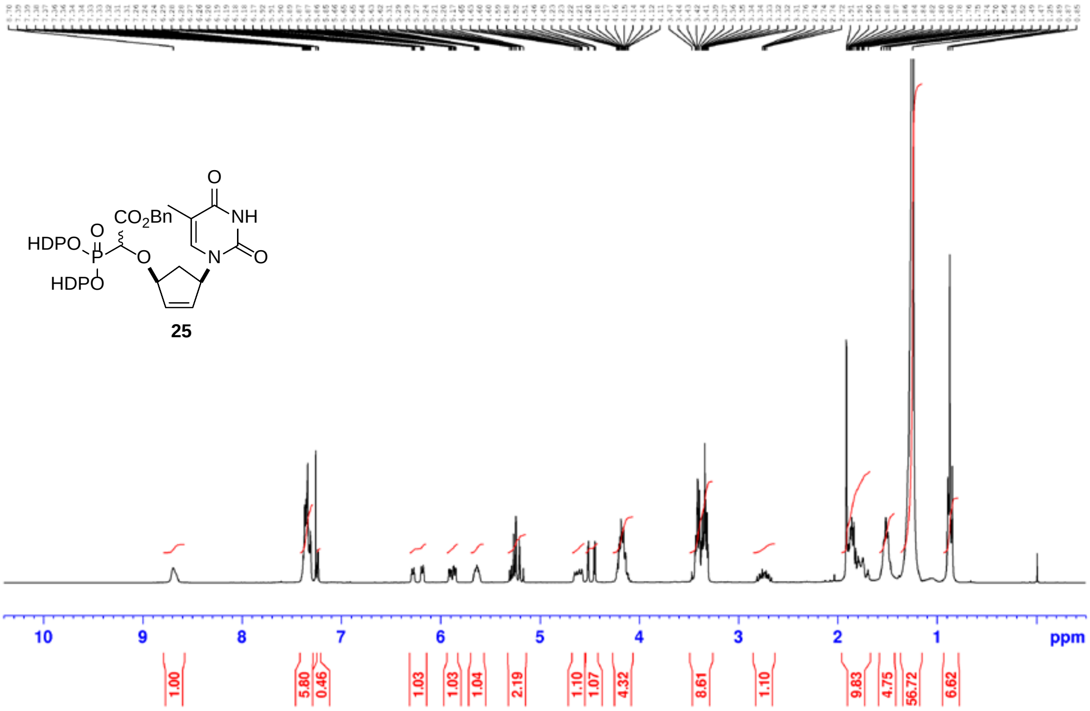

Compound **25** $^{13}C\{^{1}H\}$ NMR (75 MHz, $CDCl_3$)

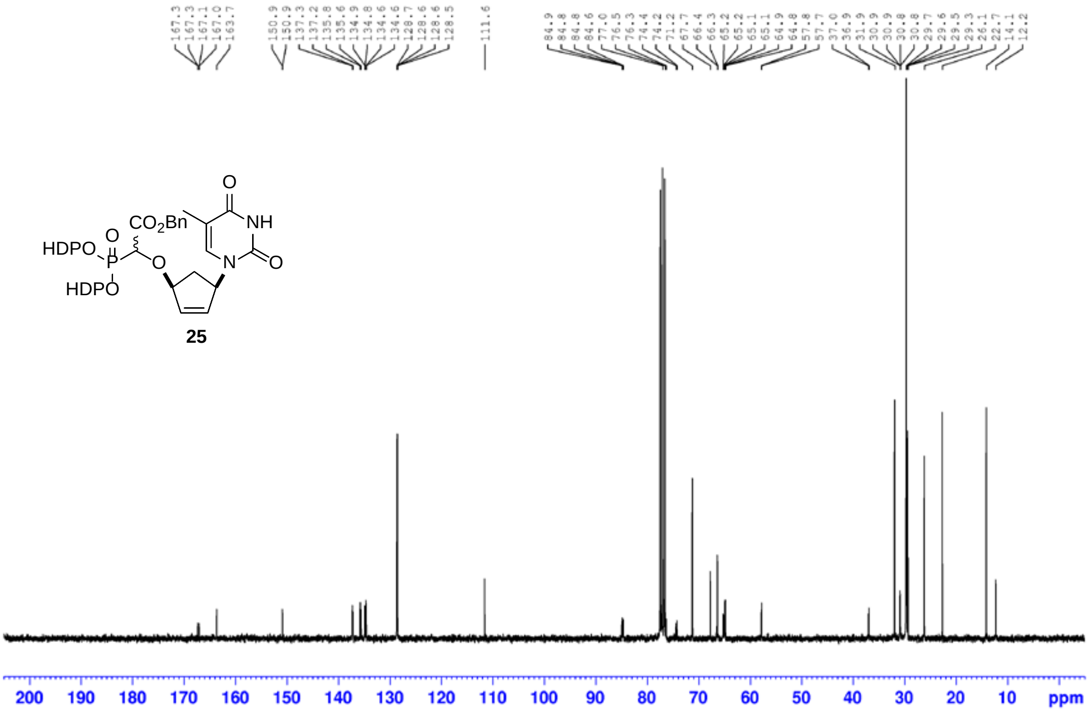

Compound **25** $^{31}P\{^{1}H\}$ NMR (121 MHz, $CDCl_3$)

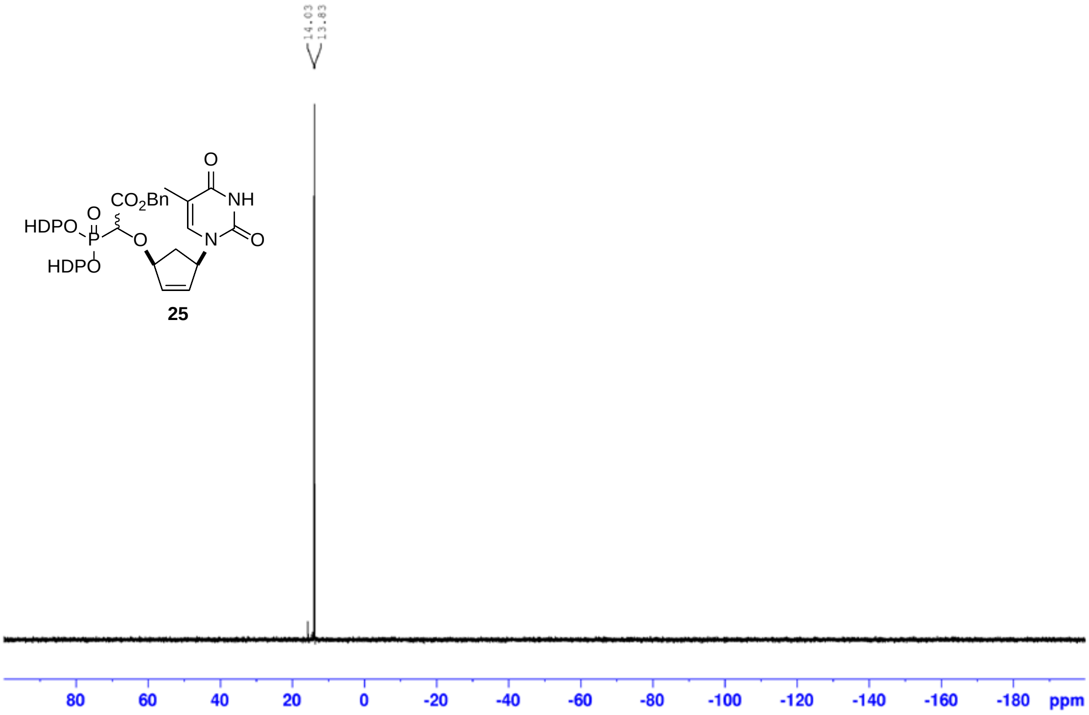

Compound **26** ^{1}H NMR (300 MHz, $CDCl_3$)

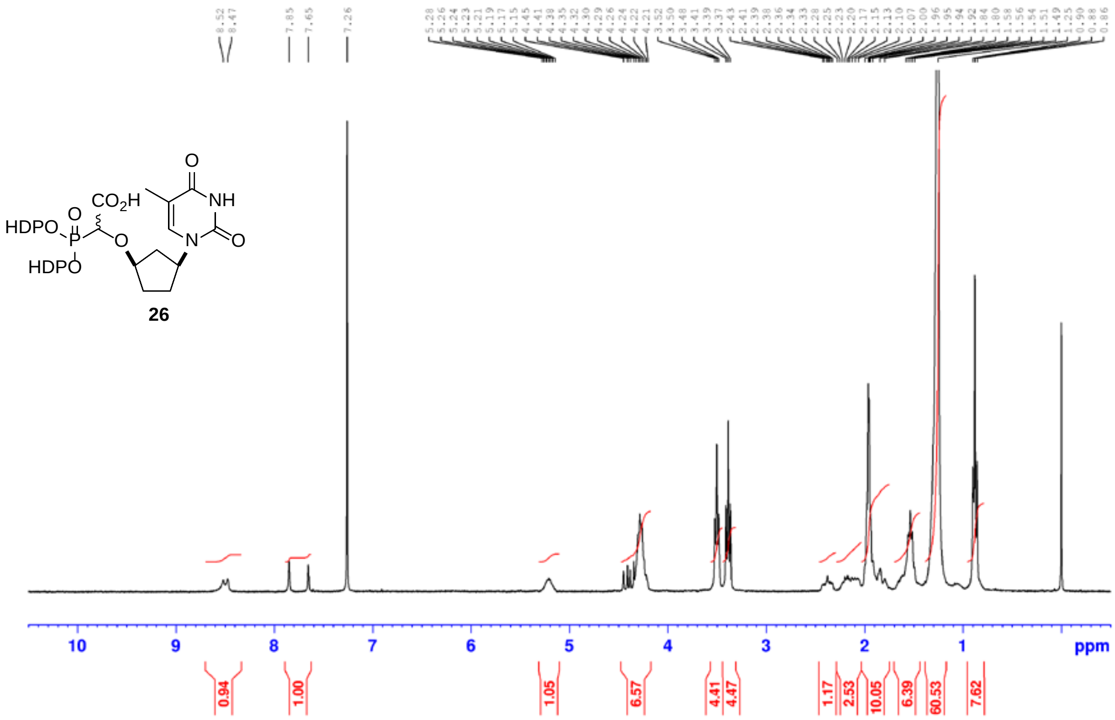

Compound **26** $^{13}C\{^1H\}$ NMR (75 MHz, $CDCl_3$)

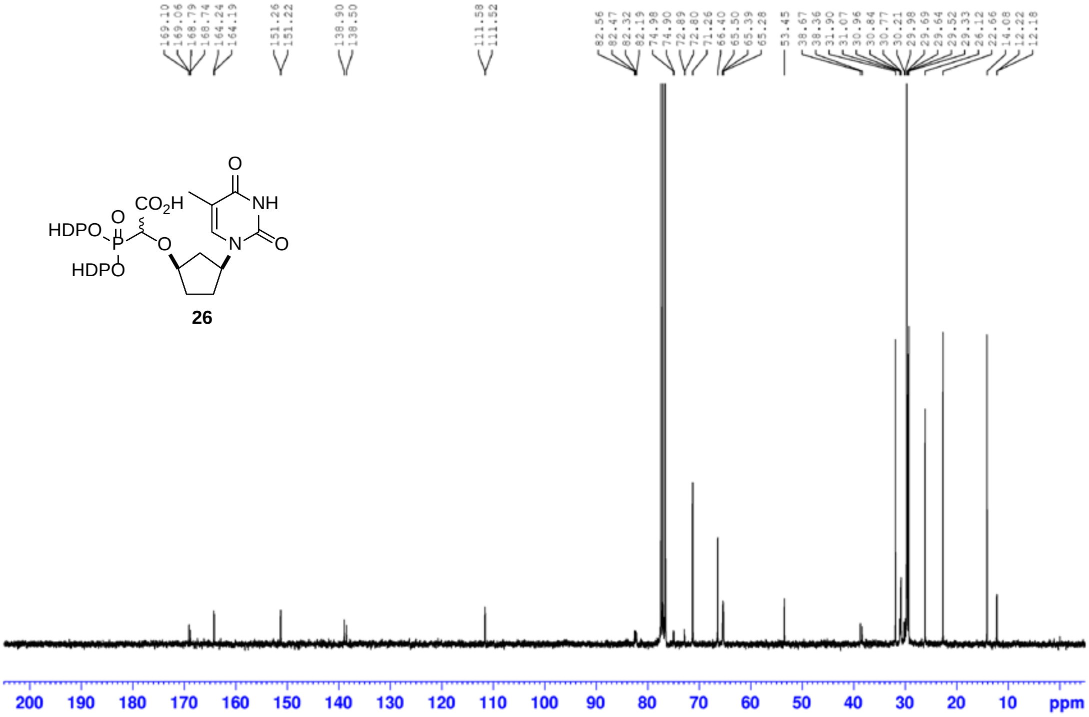

Compound **26** $^{31}P\{^1H\}$ NMR (121 MHz, $CDCl_3$)

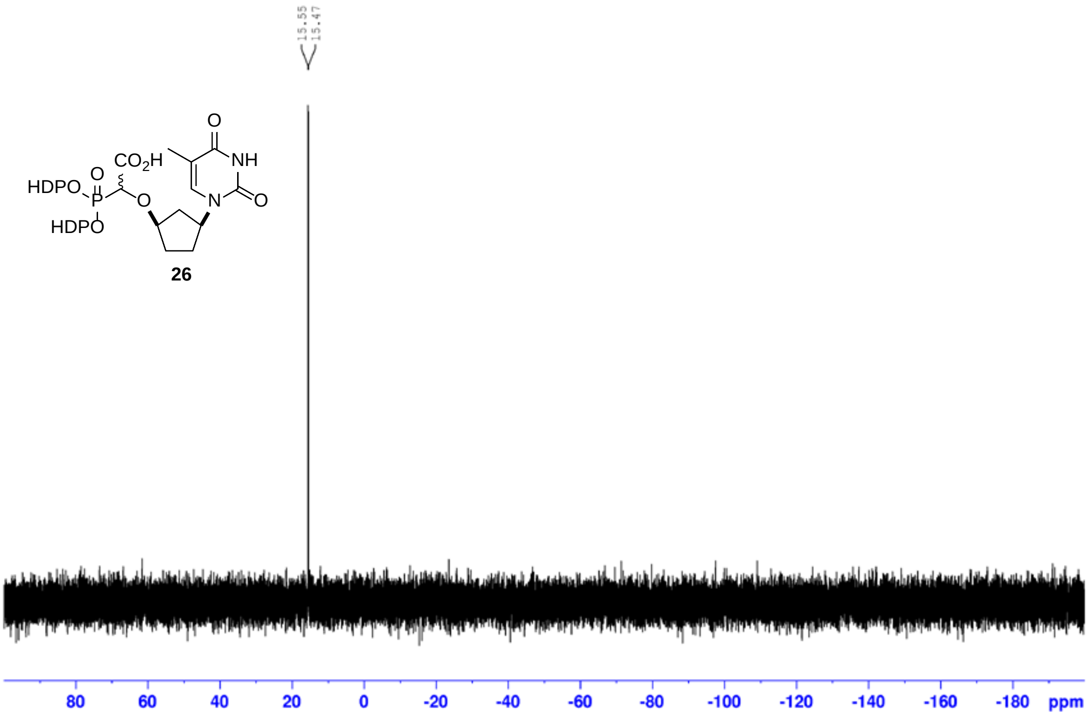

Compound **27** ^{1}H NMR (400 MHz, $CDCl_3$)

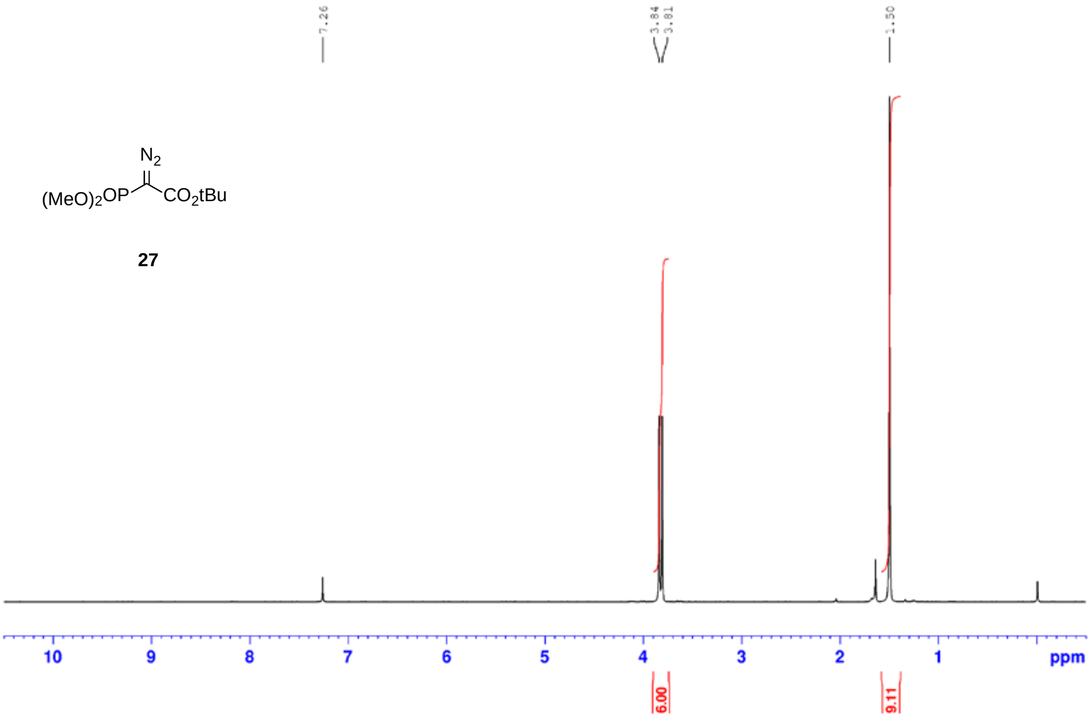

Compound **27** $^{13}C\{^{1}H\}$ NMR (100 MHz, $CDCl_3$)

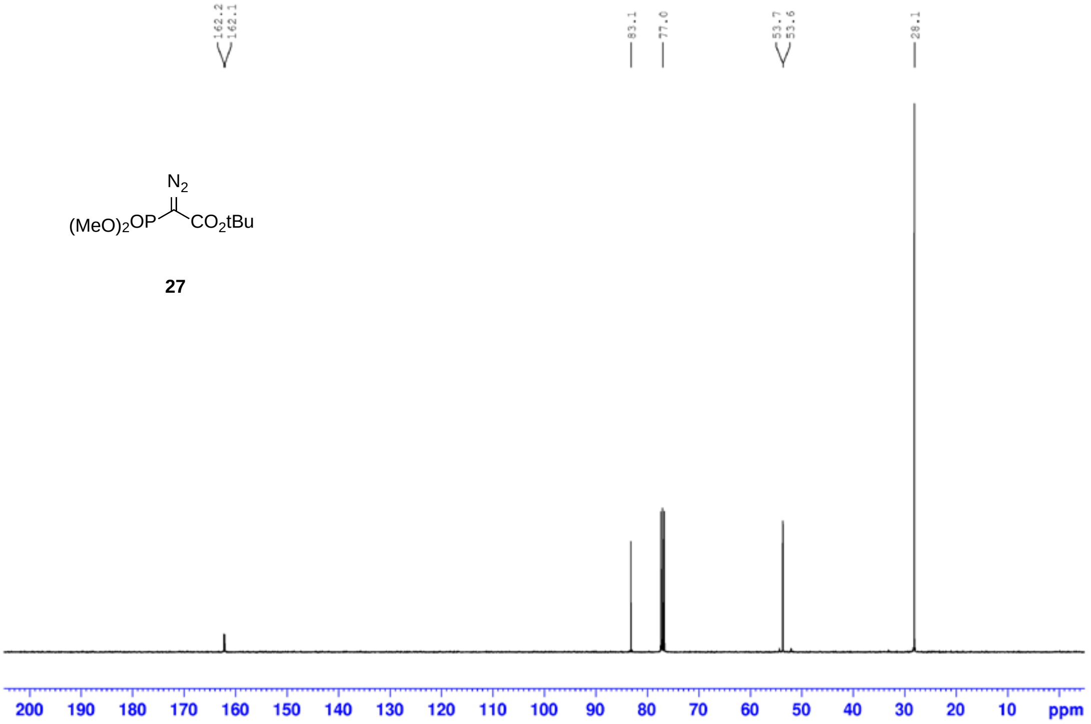

Compound **27** $^{31}P\{^1H\}$ NMR (162 MHz, $CDCl_3$)

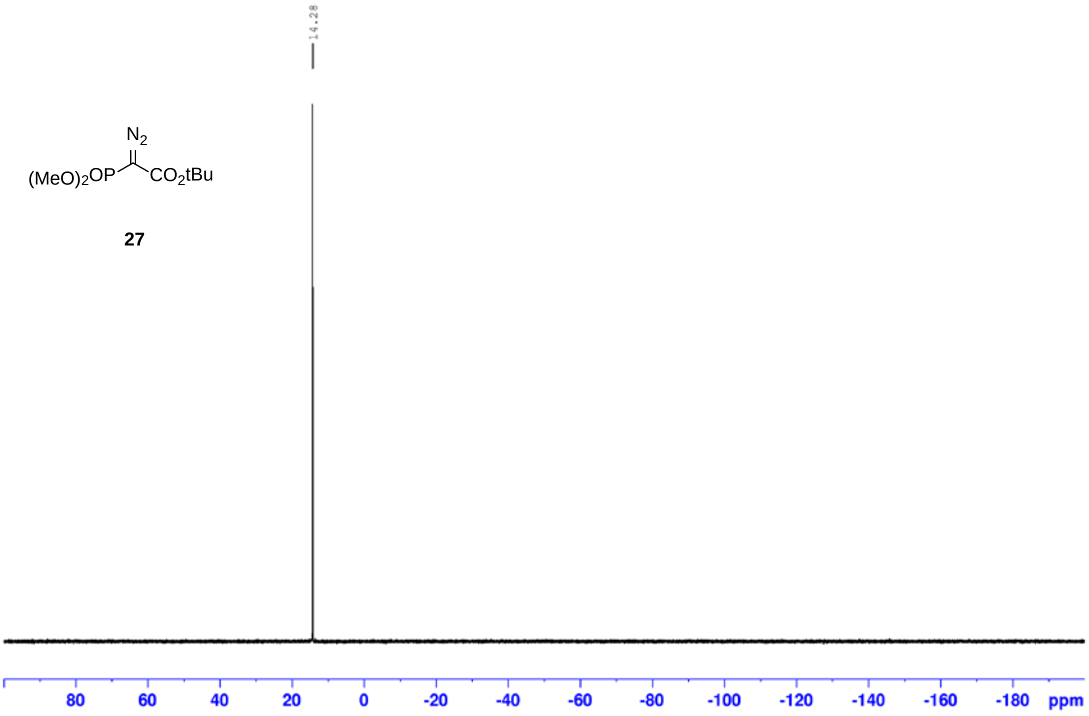

Compound **28** ^1H NMR (400 MHz, $CDCl_3$)

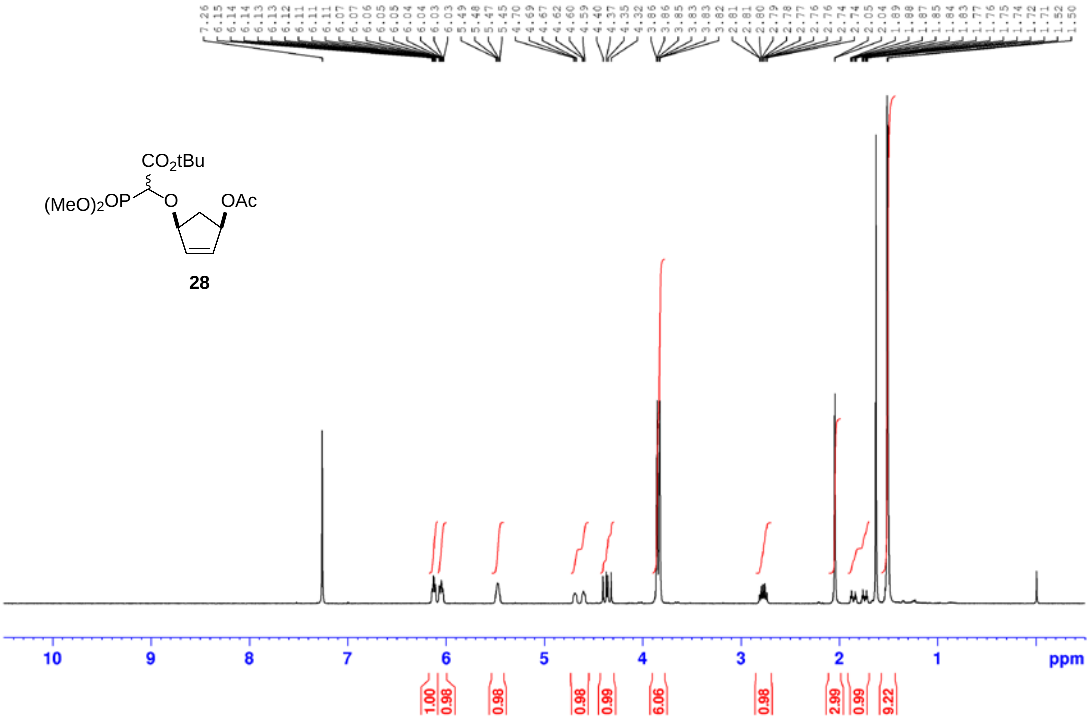

Compound **28** $^{13}C\{^1H\}$ NMR (100 MHz, $CDCl_3$)

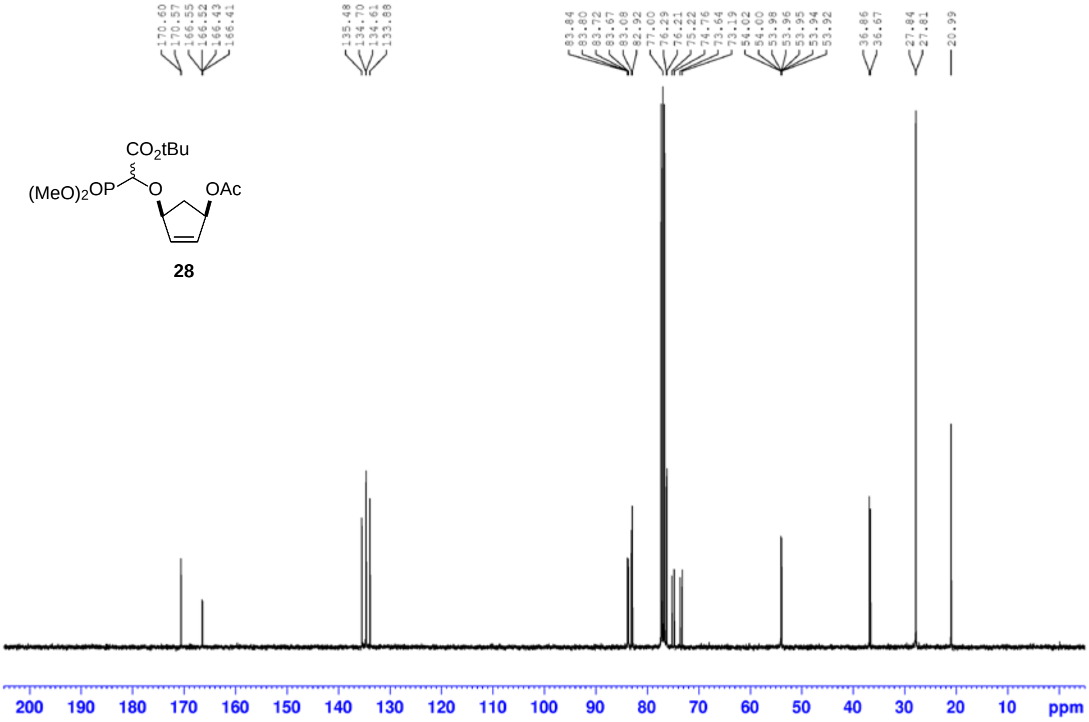

Compound **10** $^{31}P\{^1H\}$ NMR (162 MHz, $CDCl_3$)

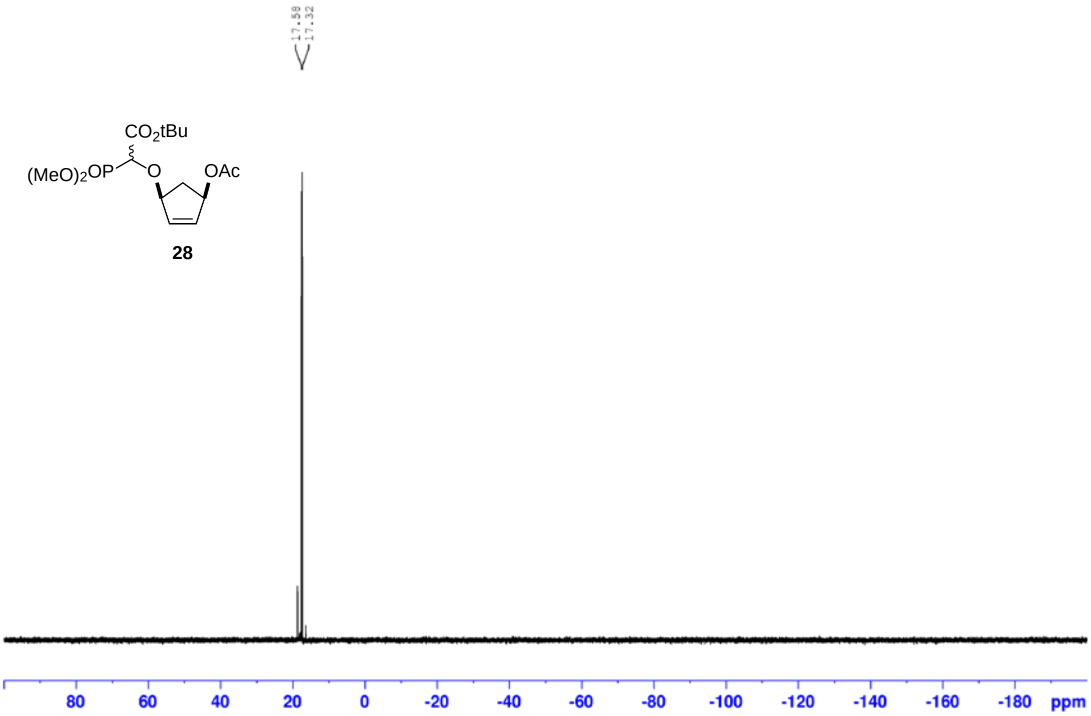

Compound **29** ^{1}H NMR (400 MHz, $CDCl_3$)

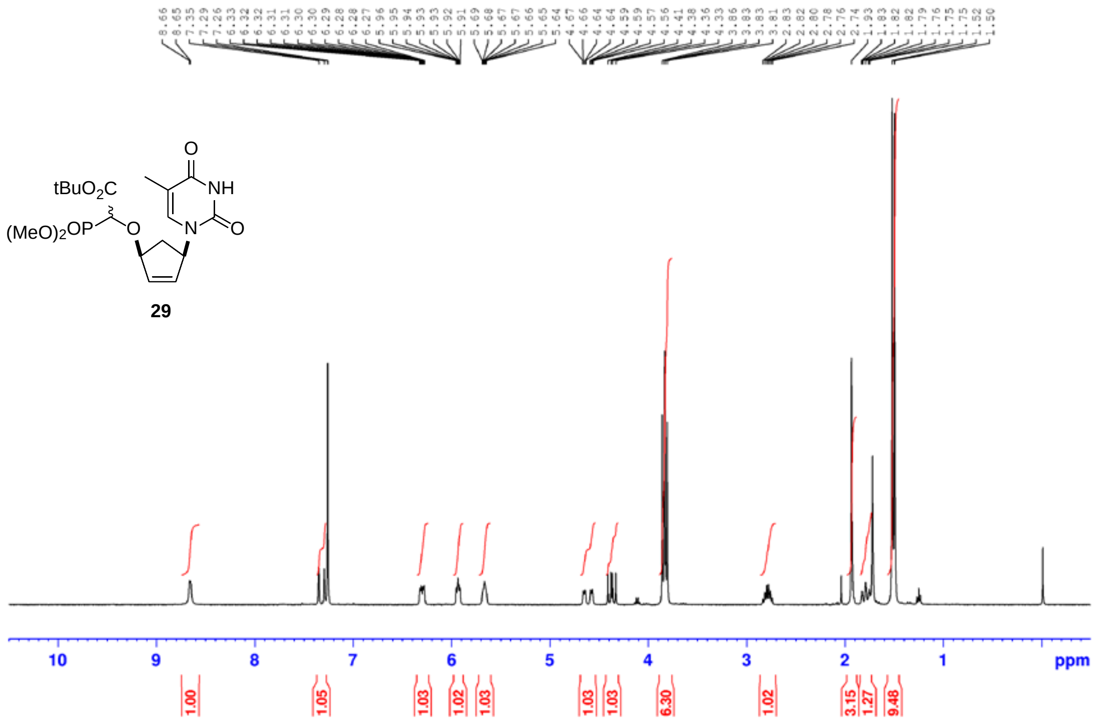

Compound **29** $^{13}C\{^{1}H\}$ NMR (100 MHz, $CDCl_3$)

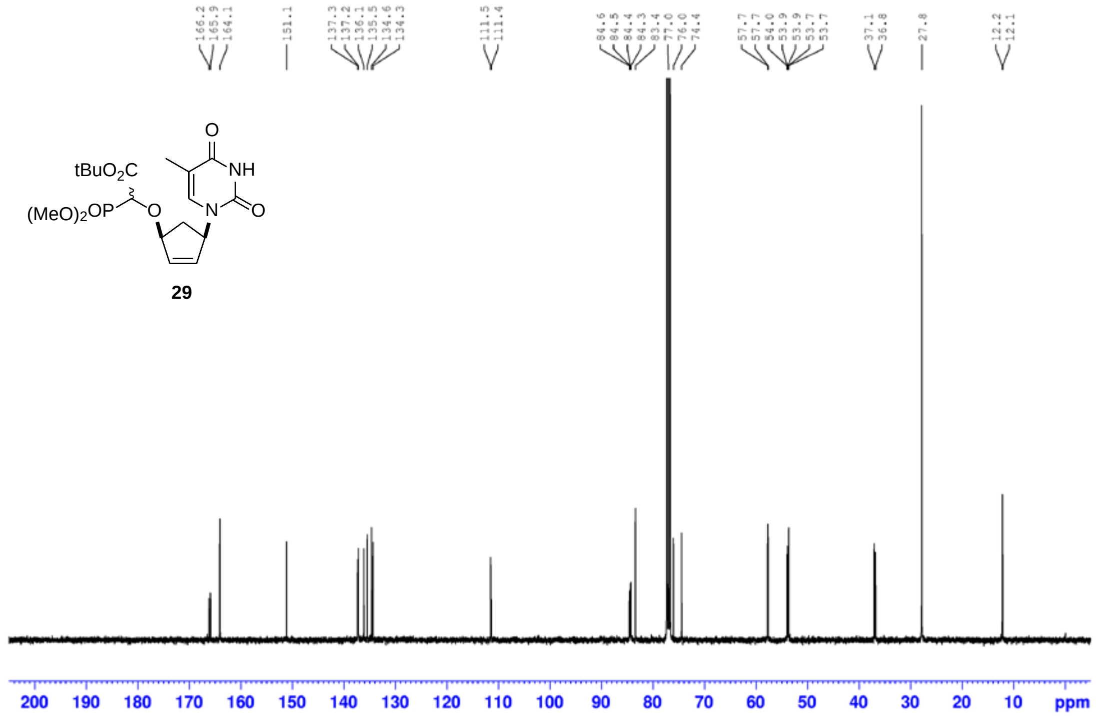

Compound **29** $^{31}P\{^{1}H\}$ NMR (162 MHz, $CDCl_3$)

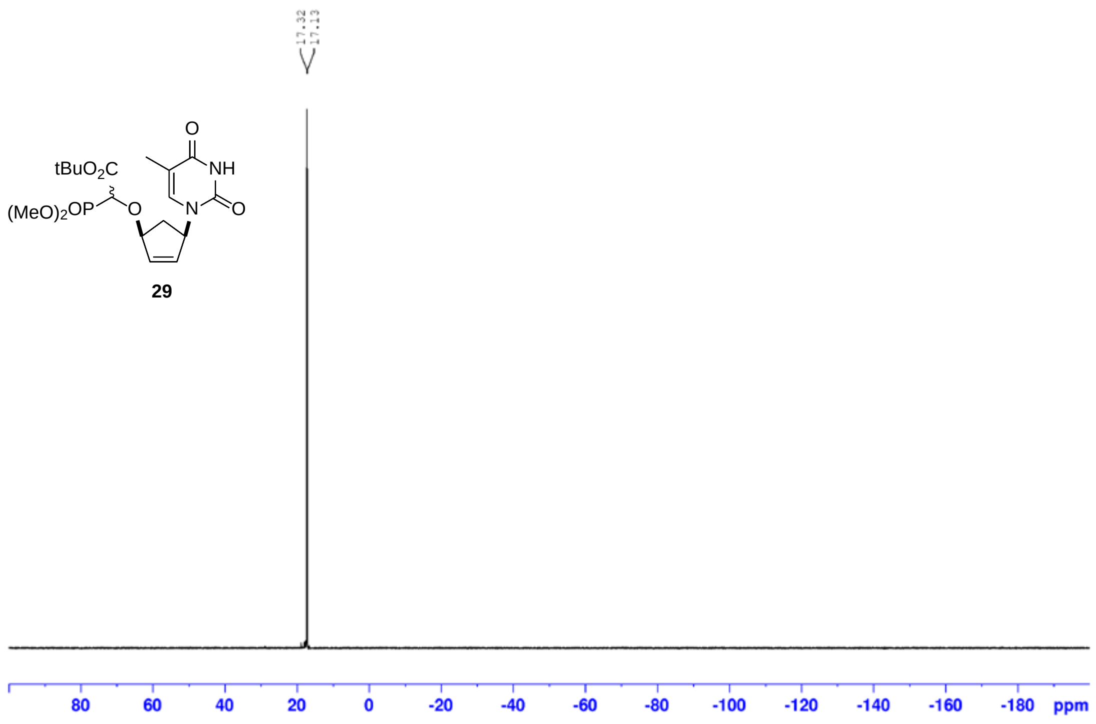

Compound **30** ^{1}H NMR (400 MHz, $CDCl_3$)

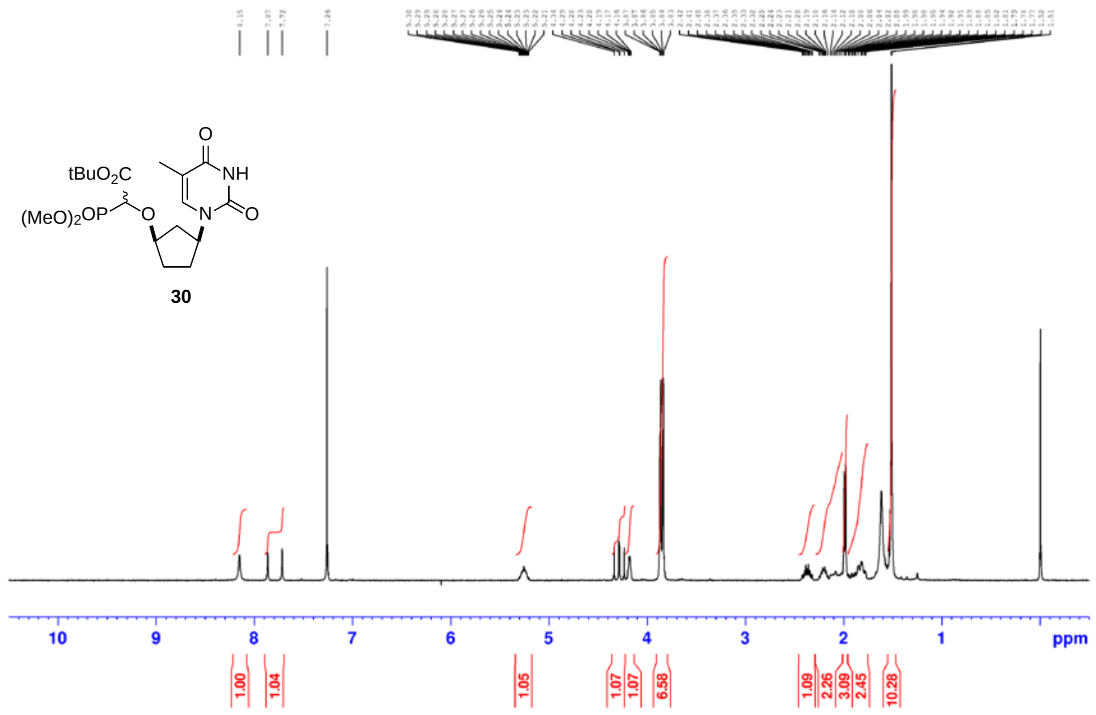

Compound **30** $^{13}C\{^{1}H\}$ NMR (100 MHz, $CDCl_3$)

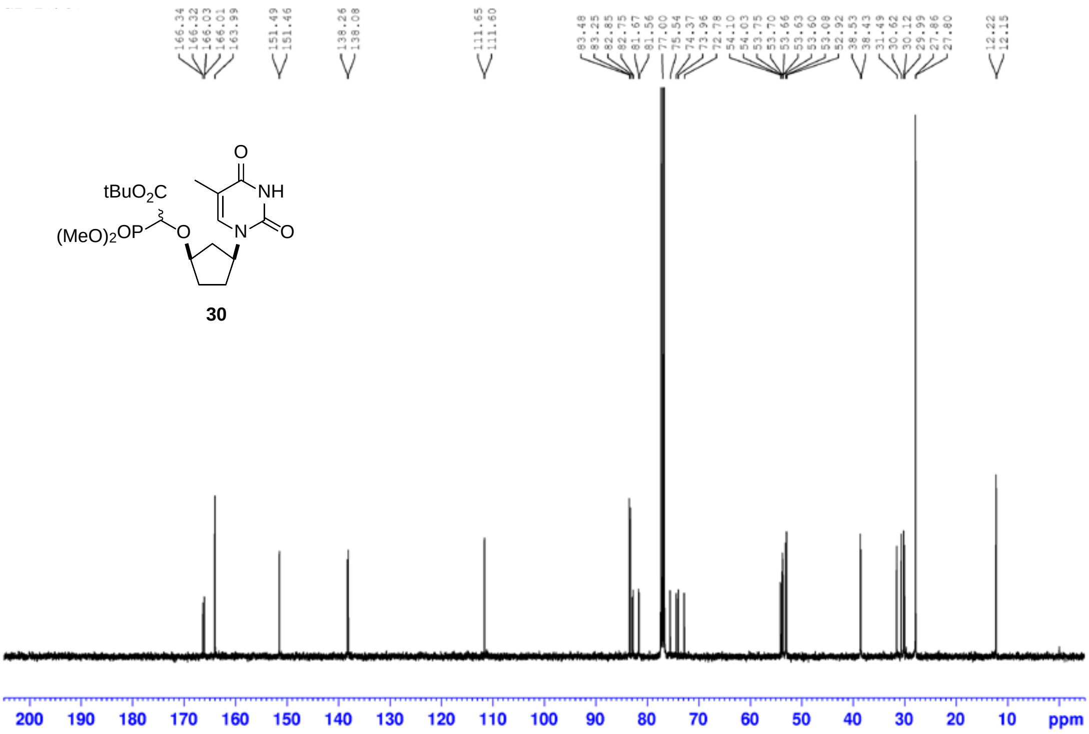

Compound **30** $^{31}P\{^{1}H\}$ NMR (162 MHz, $CDCl_3$)

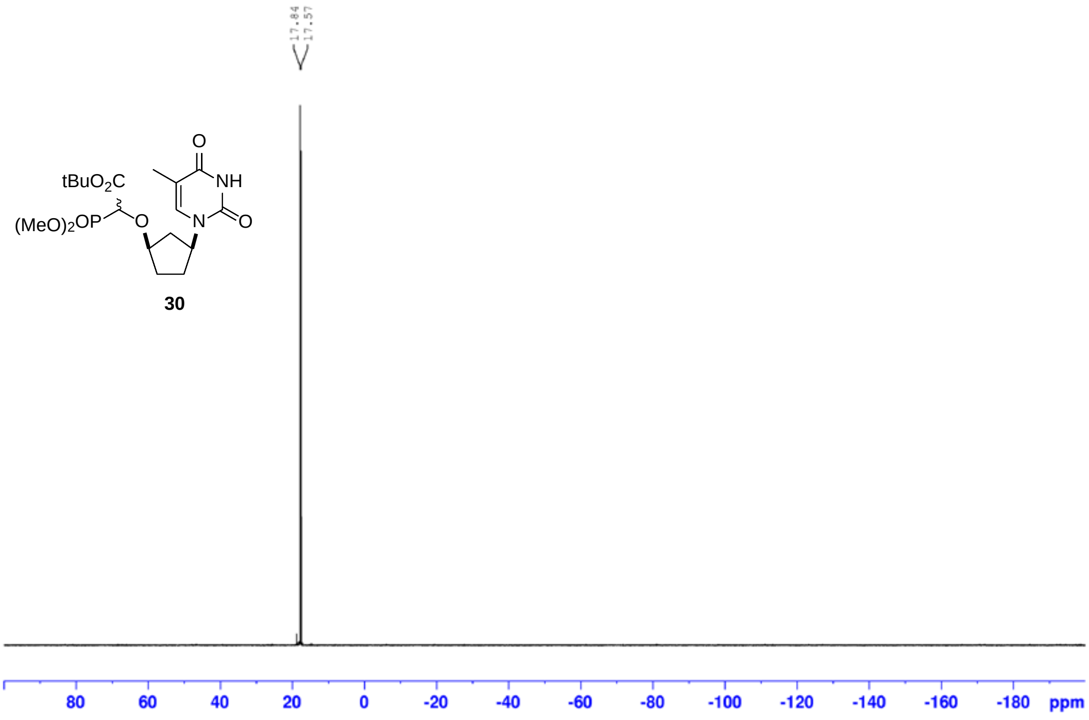

Compound **34** ^{1}H NMR (600 MHz, $CDCl_3$)

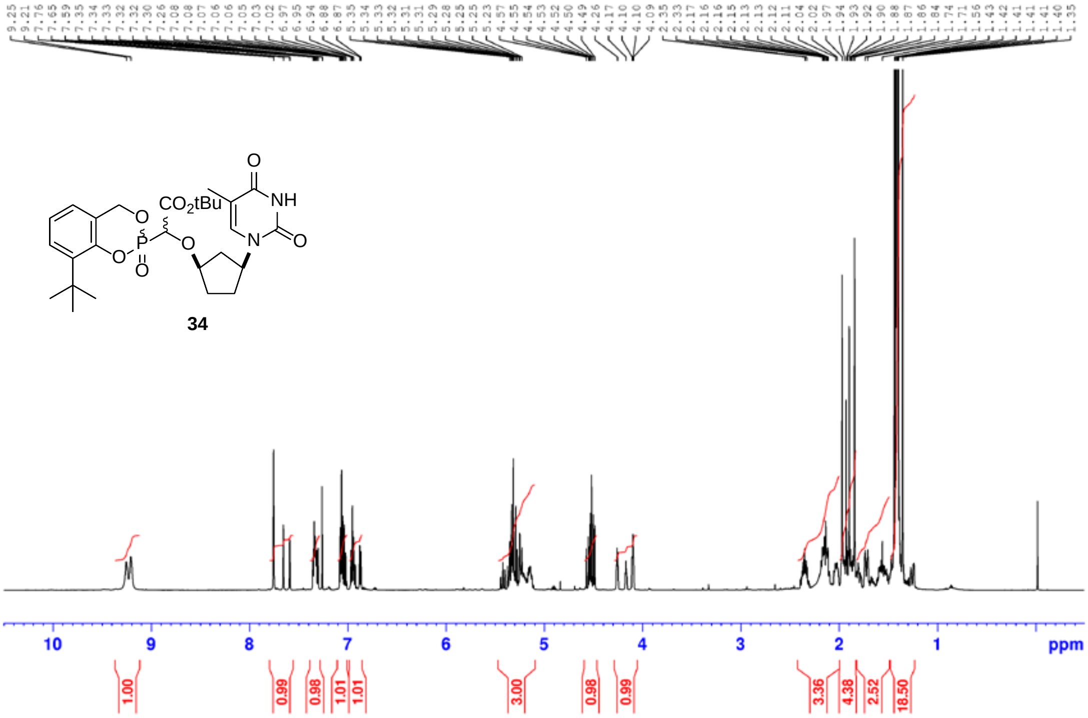

Compound **34** $^{13}C\{^{1}H\}$ NMR (150 MHz, $CDCl_3$)

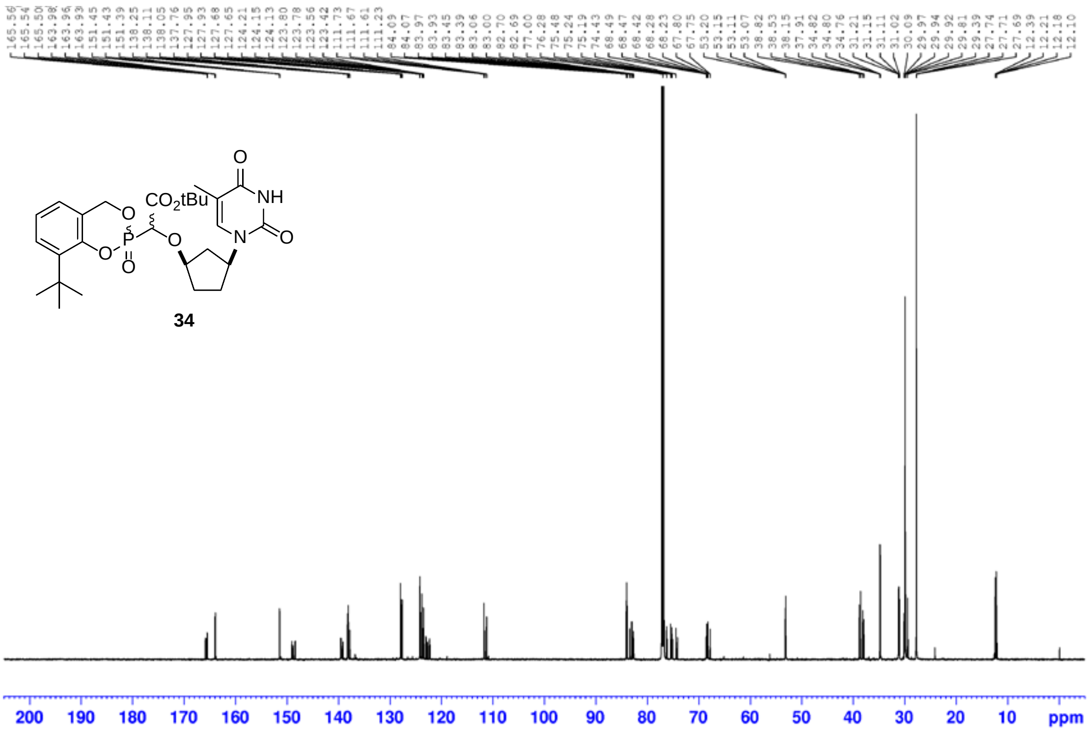

Compound **34** $^{31}P\{^{1}H\}$ NMR (121 MHz, $CDCl_3$)

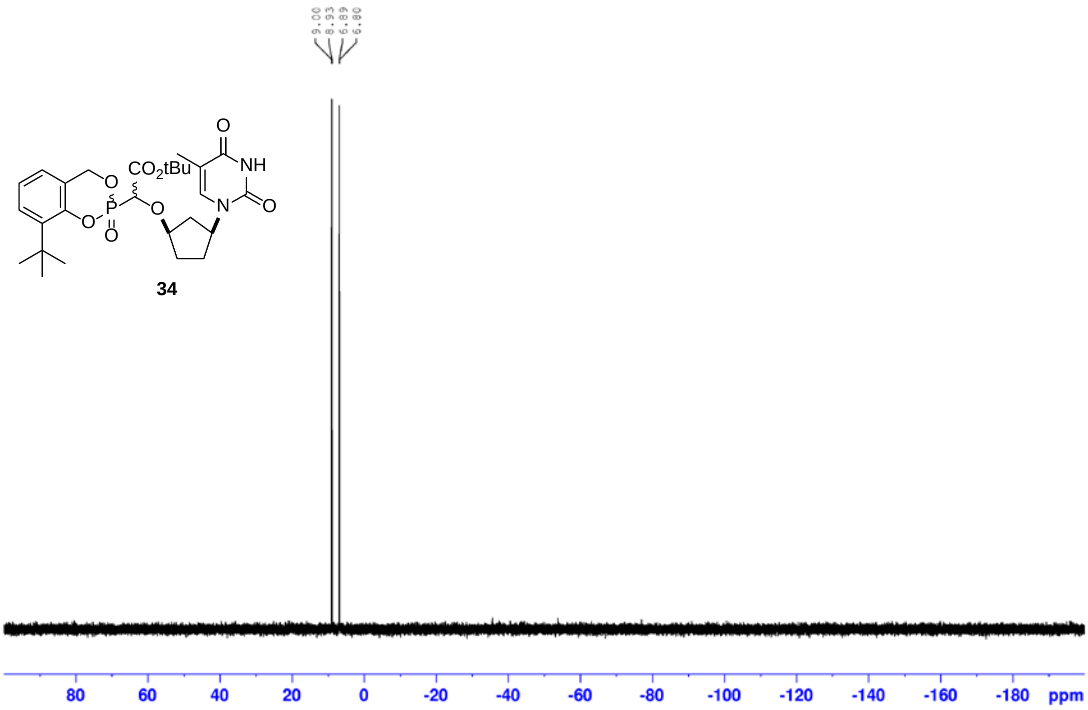

Compound **35** ^{1}H NMR (600 MHz, $CDCl_3$)

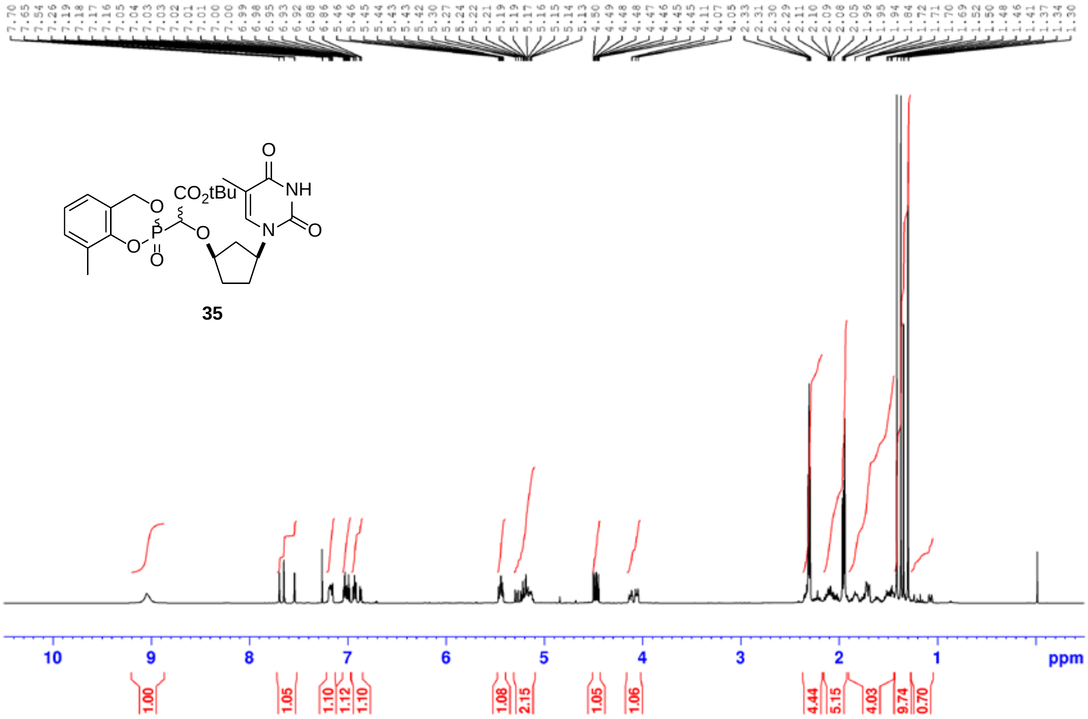

Compound **35** $^{13}C\{^{1}H\}$ NMR (150 MHz, $CDCl_3$)

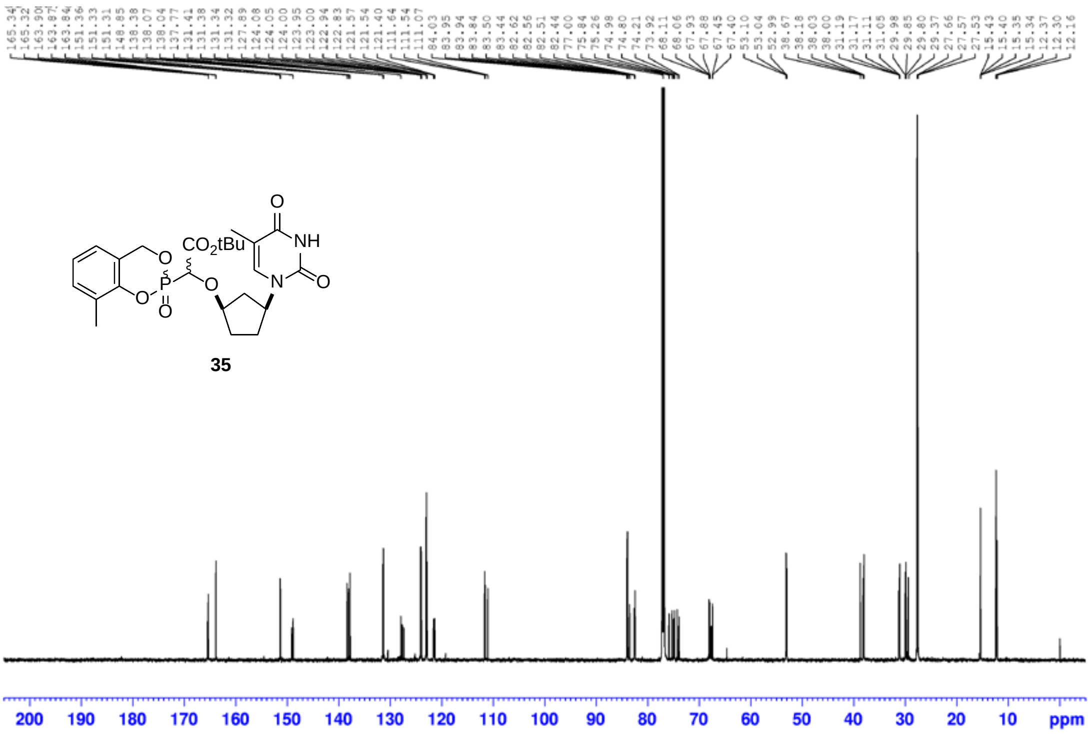

Compound **35** $^{31}P\{^{1}H\}$ NMR (121 MHz, $CDCl_3$)

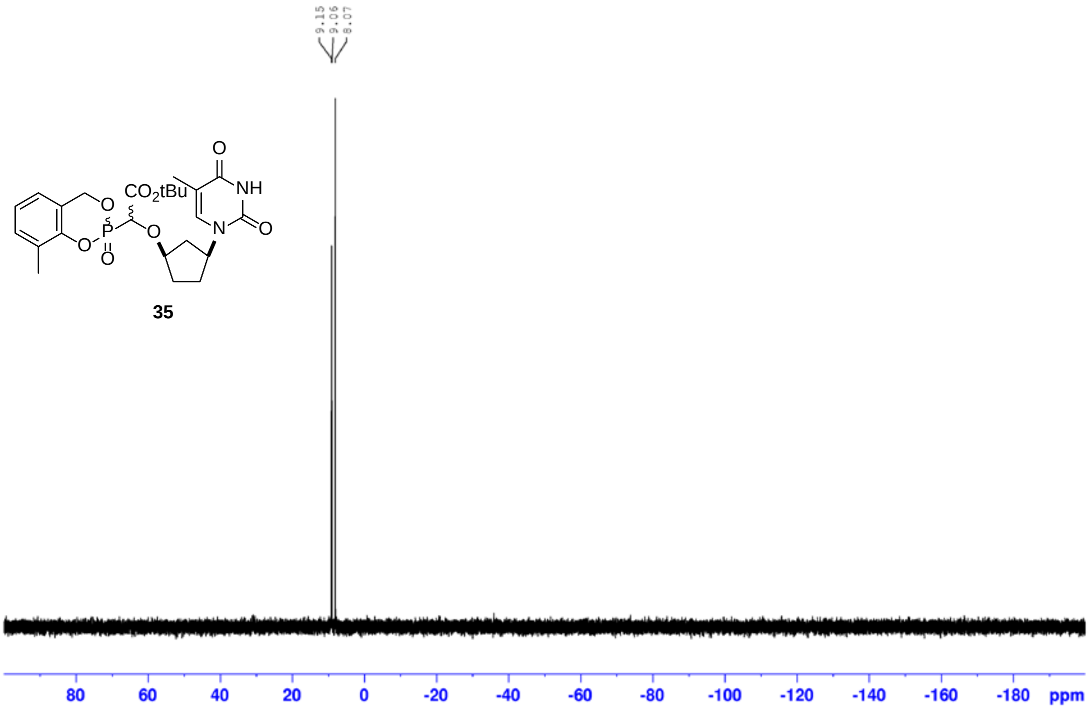

Compound **36** ^{1}H NMR (600 MHz, $CDCl_3$)

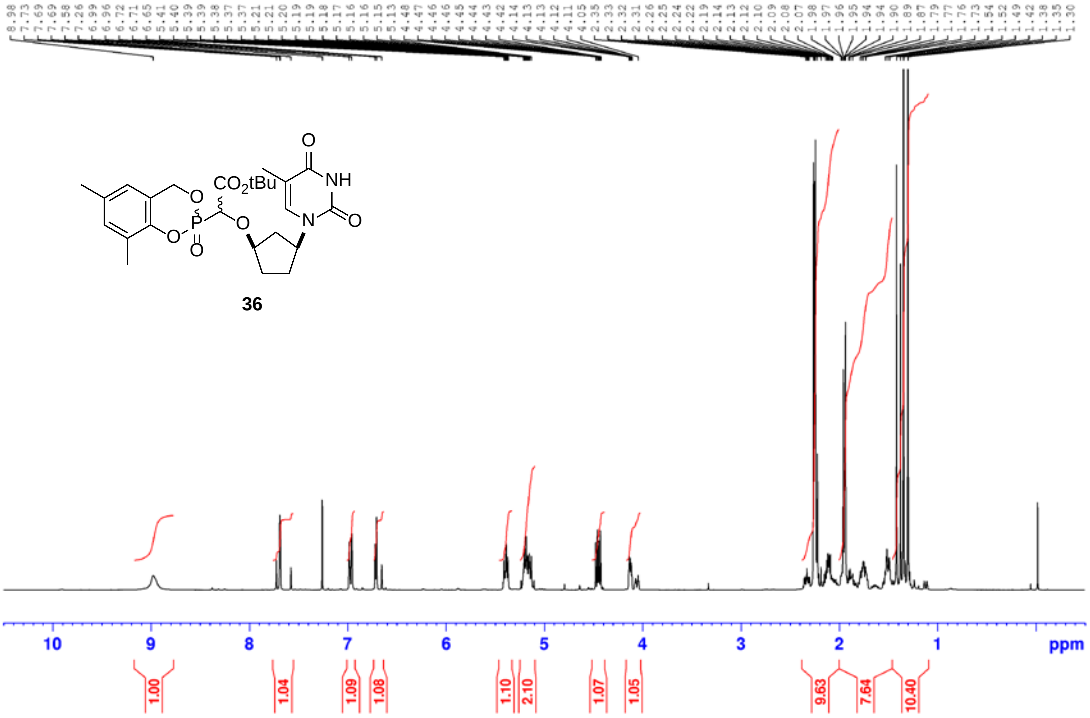

Compound **36** $^{13}C\{^{1}H\}$ NMR (150 MHz, $CDCl_3$)

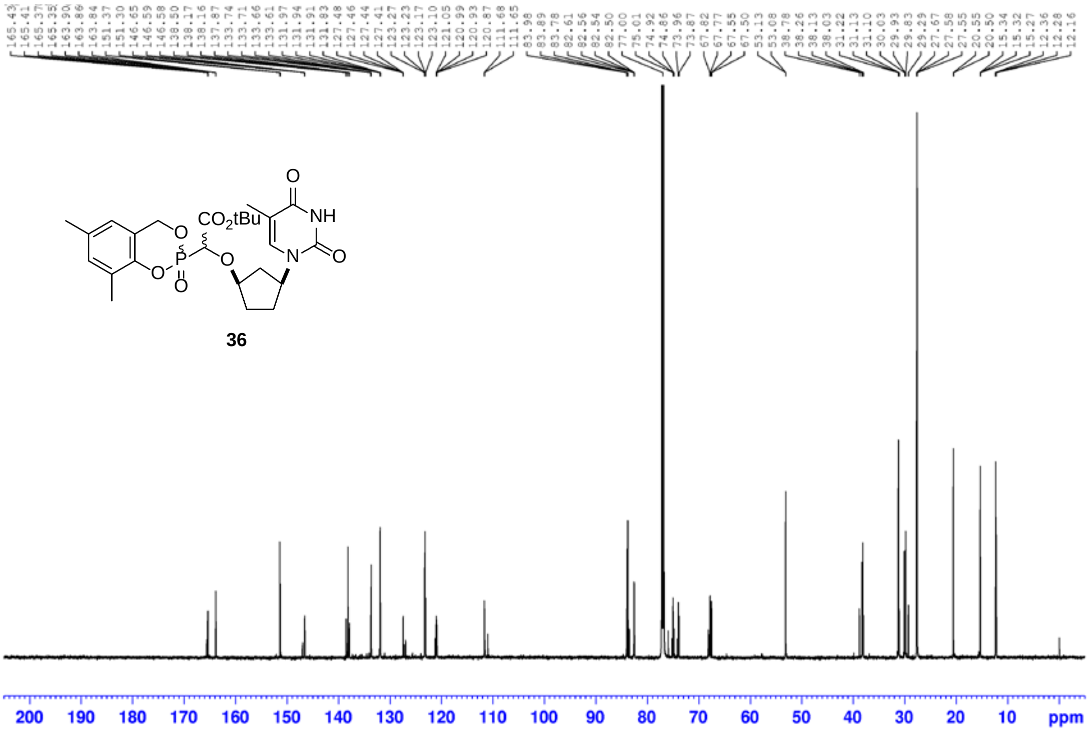

Compound **36** $^{31}P\{^{1}H\}$ NMR (121 MHz, $CDCl_3$)

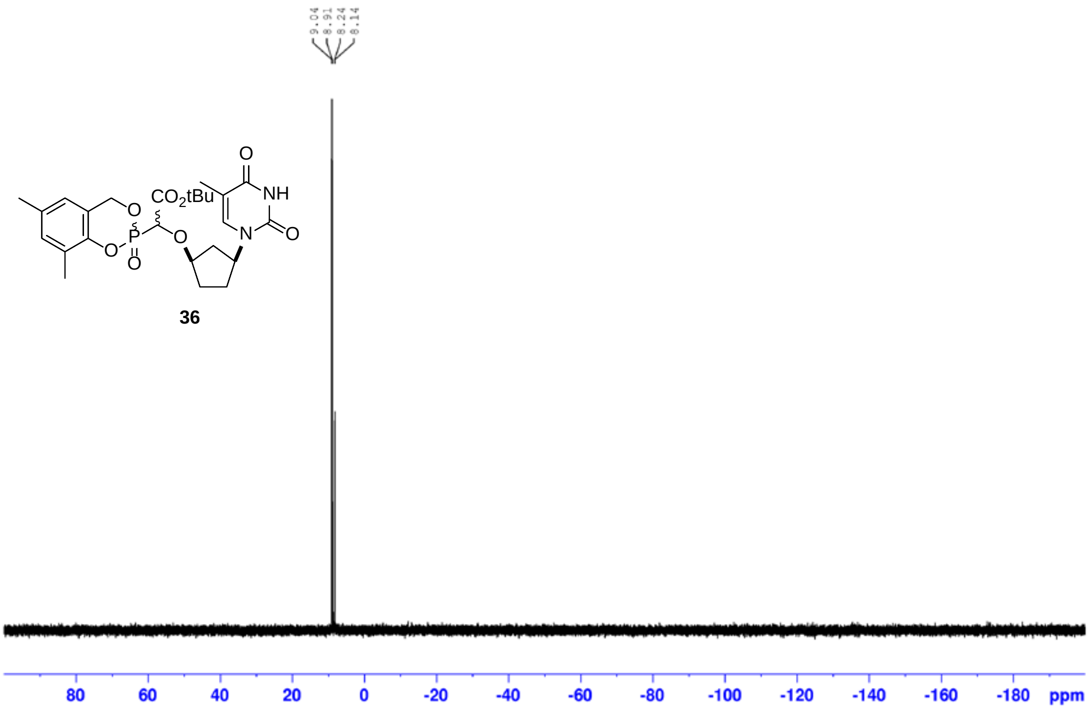

Compound **37** ^{1}H NMR (600 MHz, $CDCl_3$)

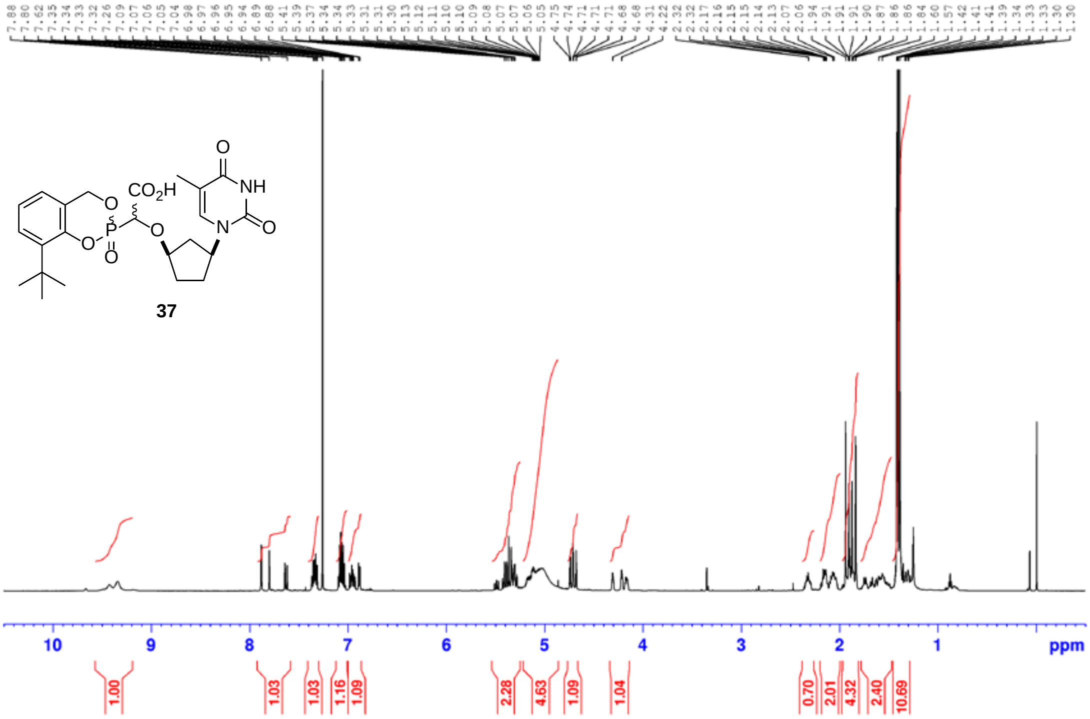

Compound **37** $^{13}C\{^{1}H\}$ NMR (150 MHz, $CDCl_3$)

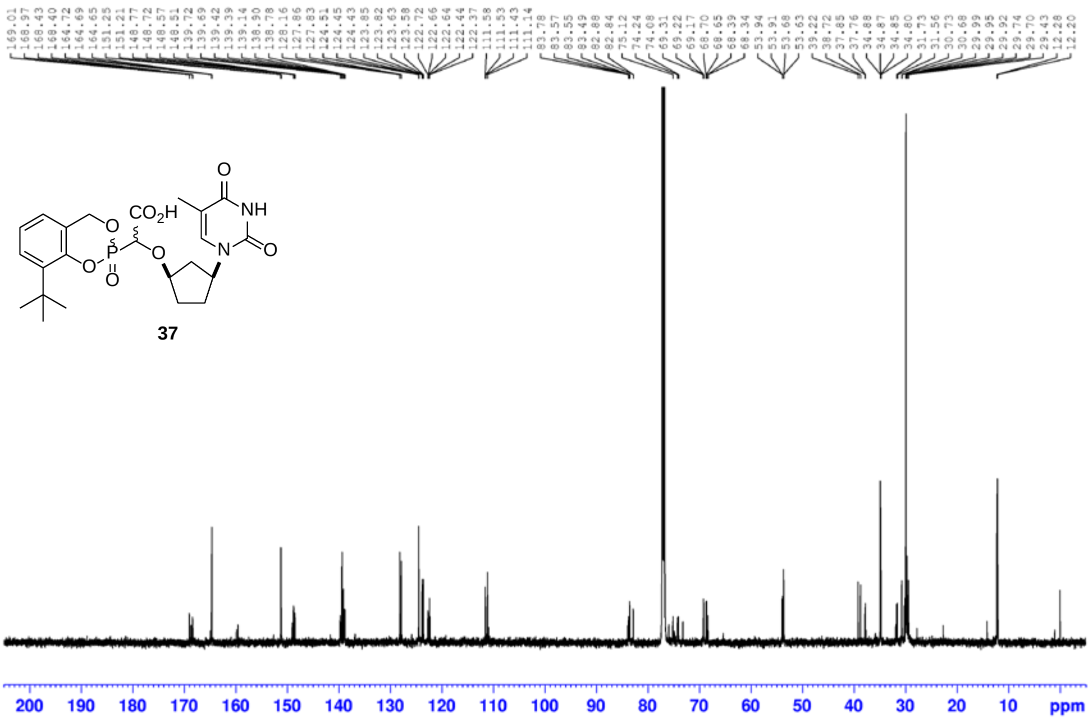

Compound **37** $^{31}P\{^{1}H\}$ NMR (121 MHz, $CDCl_3$)

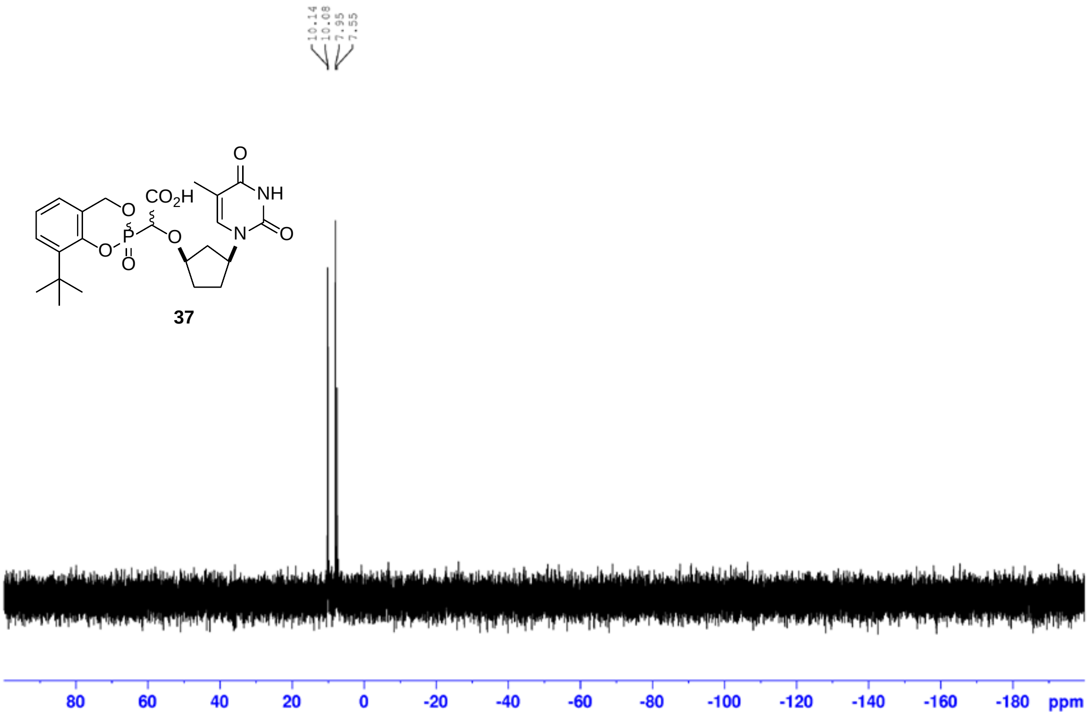

Compound **38** ^{1}H NMR (400 MHz, $CDCl_3$)

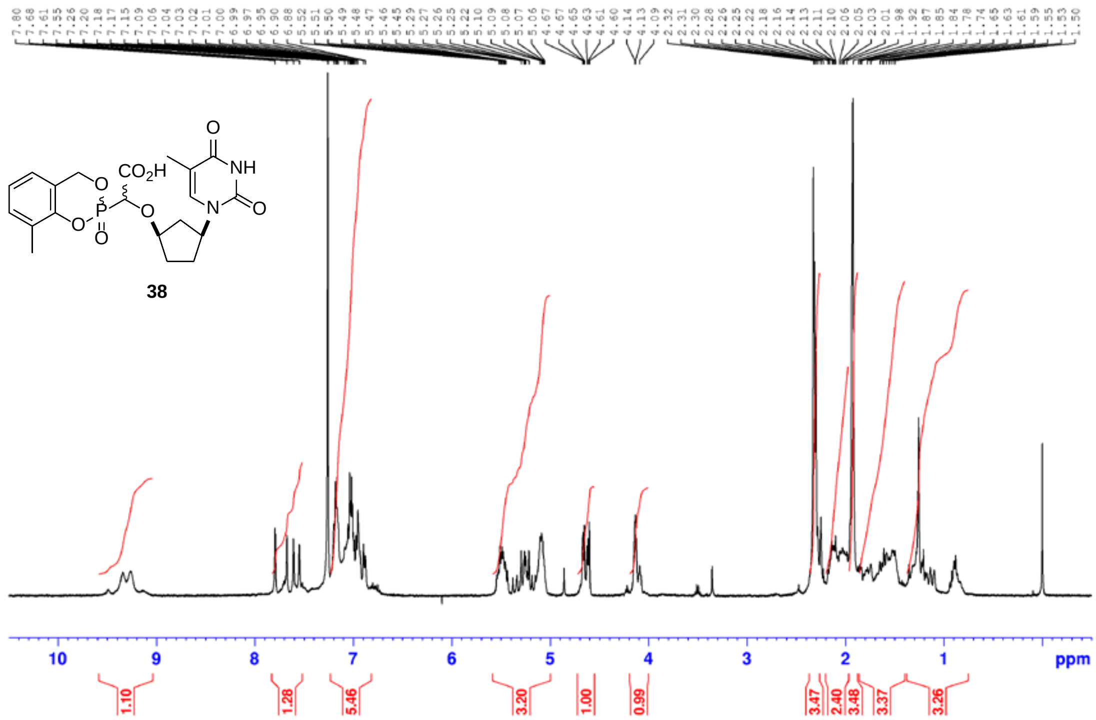

Compound **38** $^{13}C\{^{1}H\}$ NMR (150 MHz, $CDCl_3$)

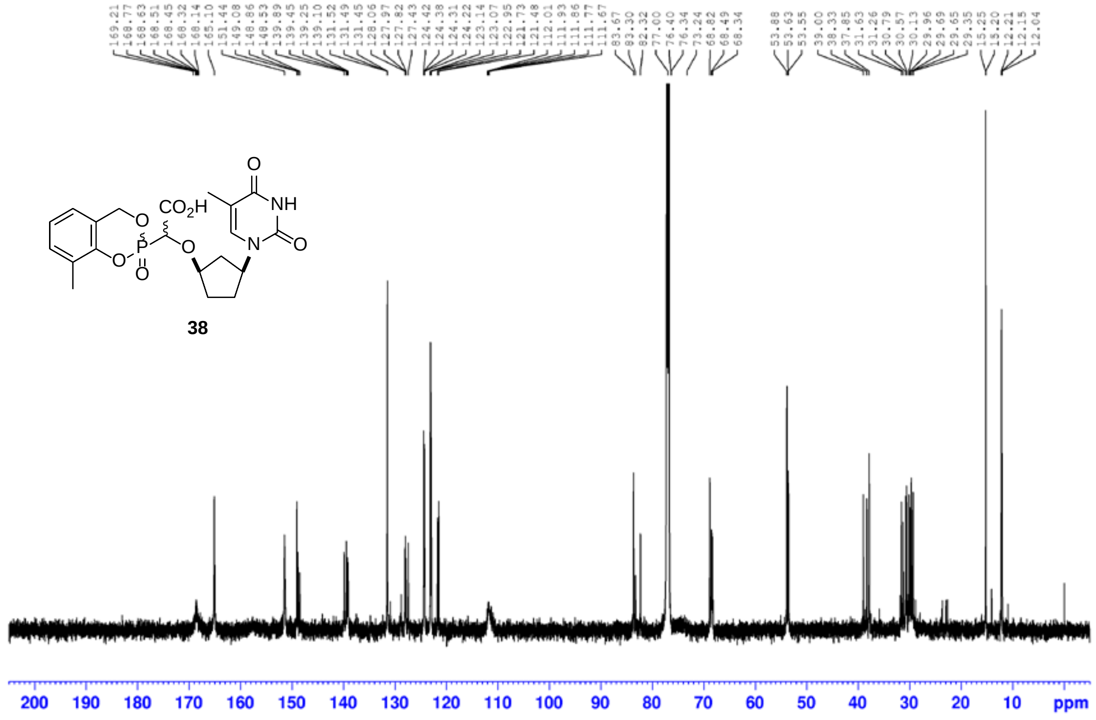

Compound **38** $^{31}P\{^{1}H\}$ NMR (162 MHz, $CDCl_3$)

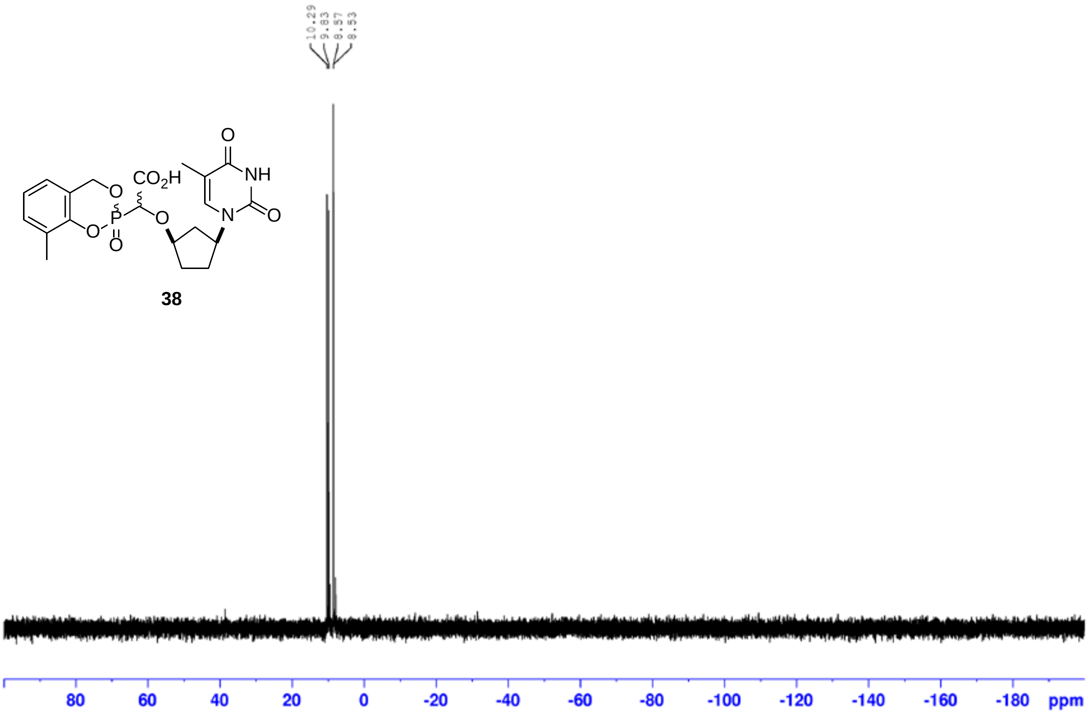

Compound **39** ^{1}H NMR (600 MHz, $CDCl_3$)

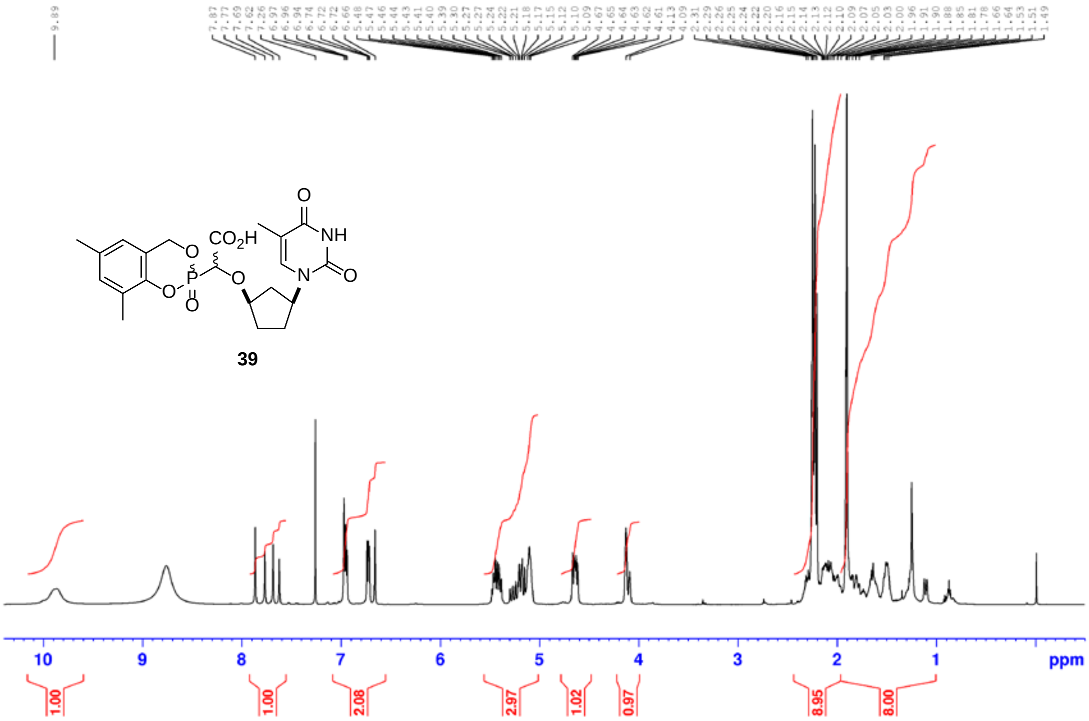

Compound **39** $^{13}C\{^1H\}$ NMR (150 MHz, $CDCl_3$)

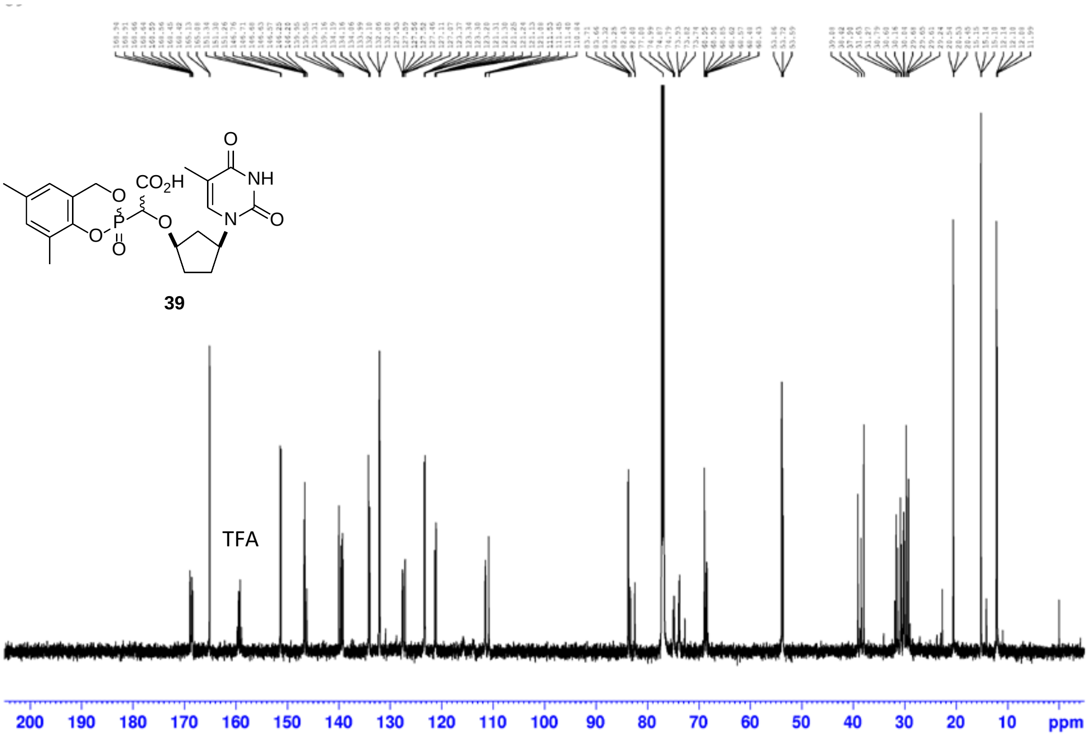

Compound **39** $^{31}P\{^1H\}$ NMR (162 MHz, $CDCl_3$)

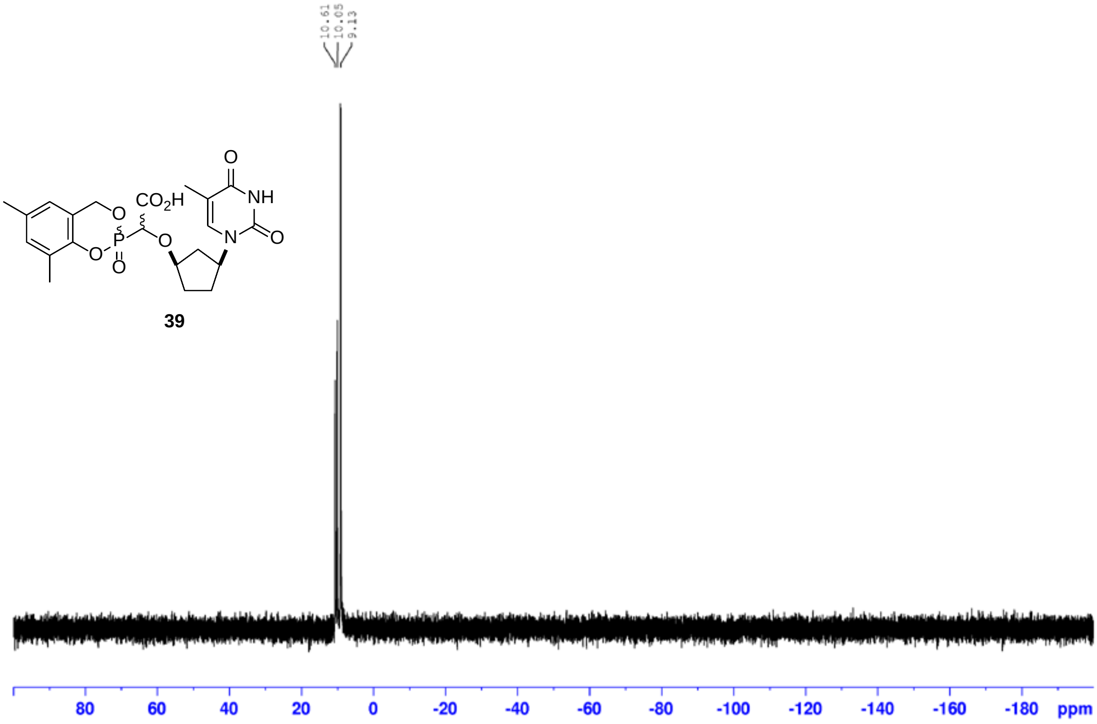

Compound **40** ^{1}H NMR (400 MHz, $CDCl_3$)

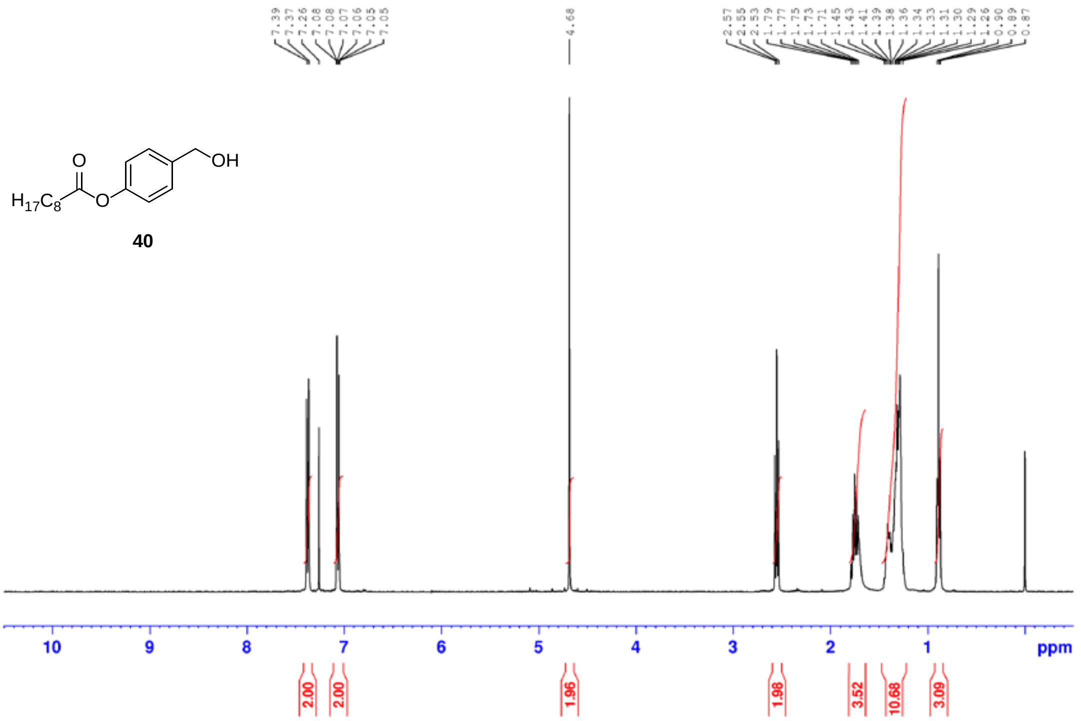

Compound **40** $^{13}C\{^{1}H\}$ NMR (100 MHz, $CDCl_3$)

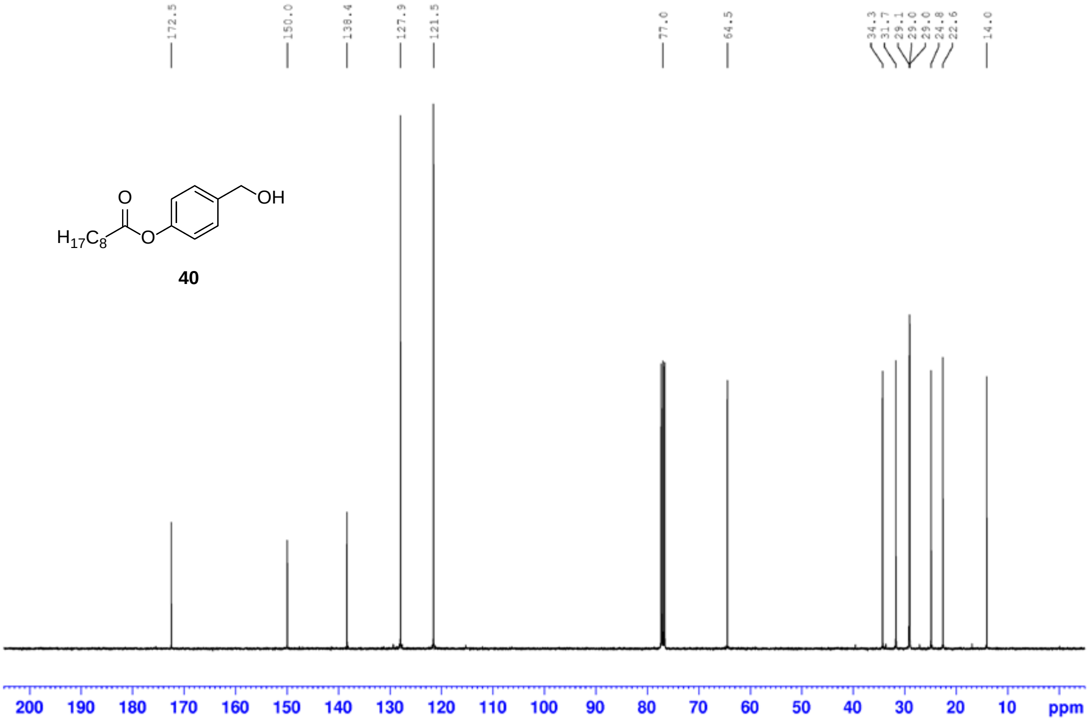

Compound **41** ^{1}H NMR (400 MHz, $CDCl_3$)

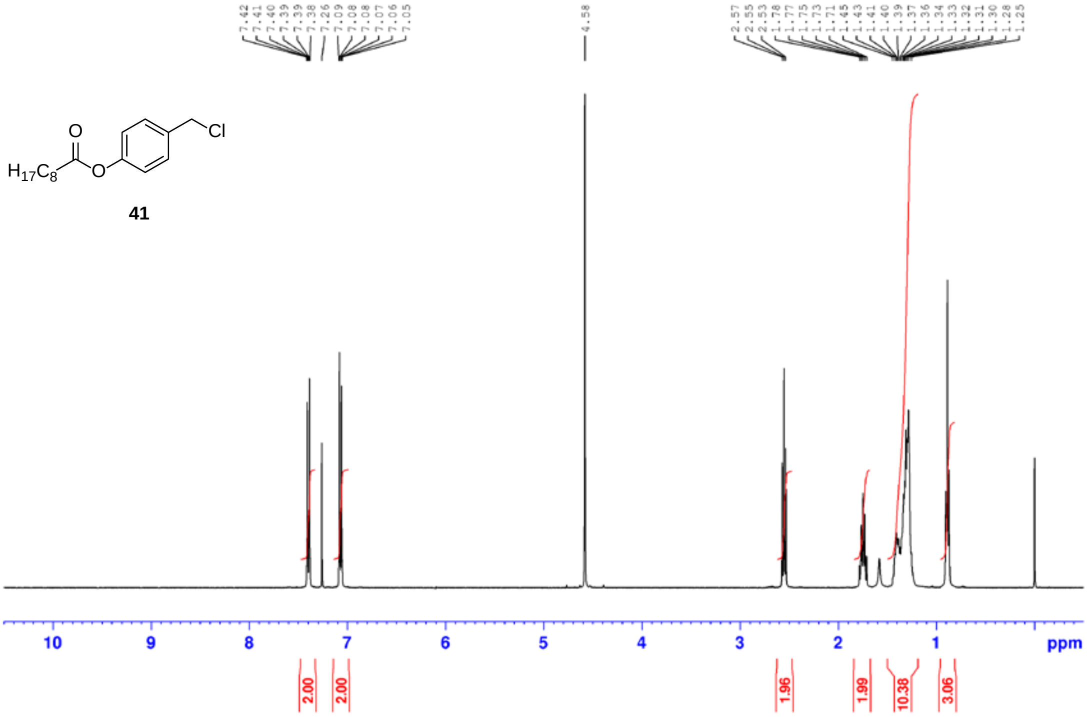

Compound **41** 13C{1H} NMR (100 MHz, $CDCl_3$)

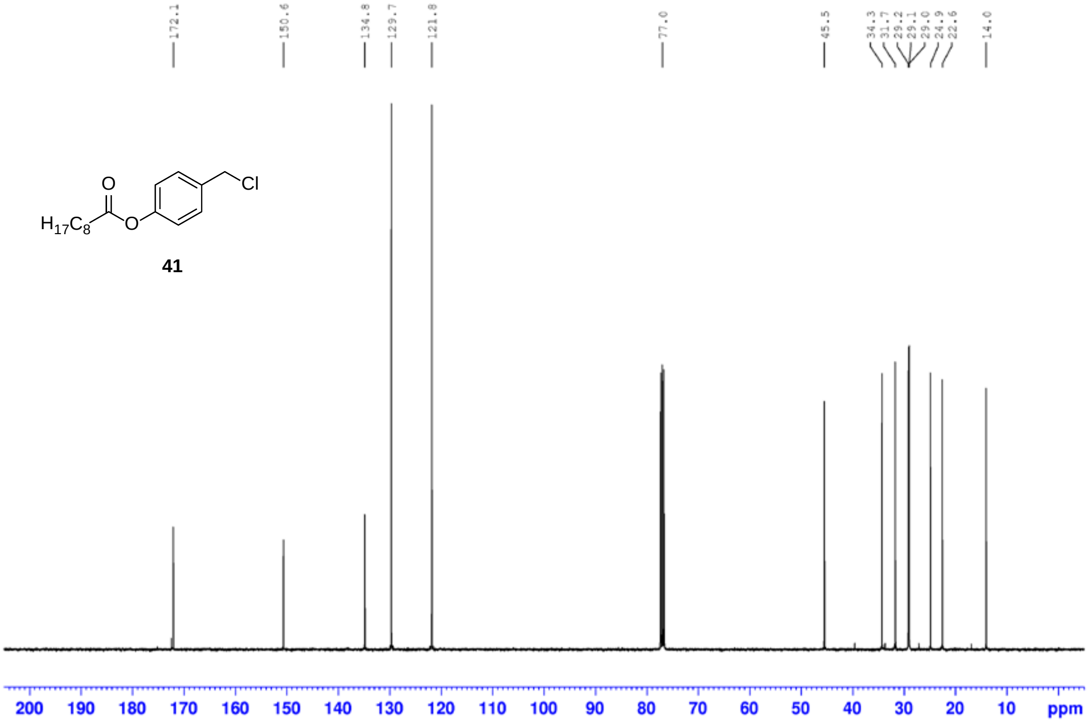

Compound **42** ^{1}H NMR (400 MHz, $CDCl_3$)

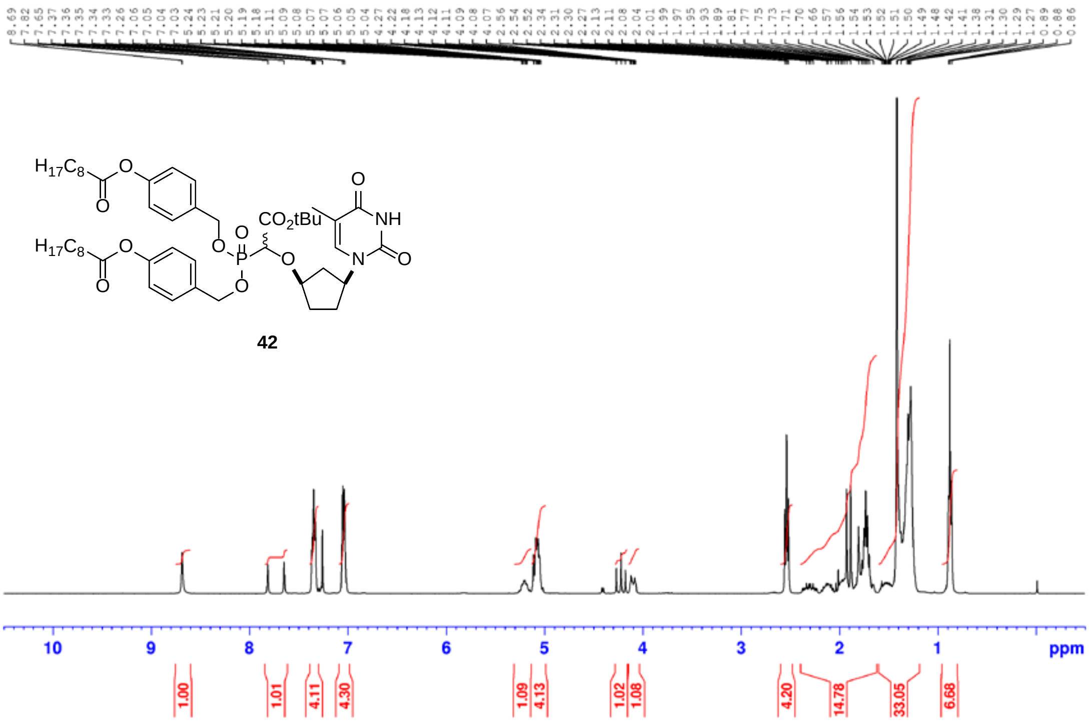

Compound **42** $^{13}C\{^{1}H\}$ NMR (100 MHz, $CDCl_3$)

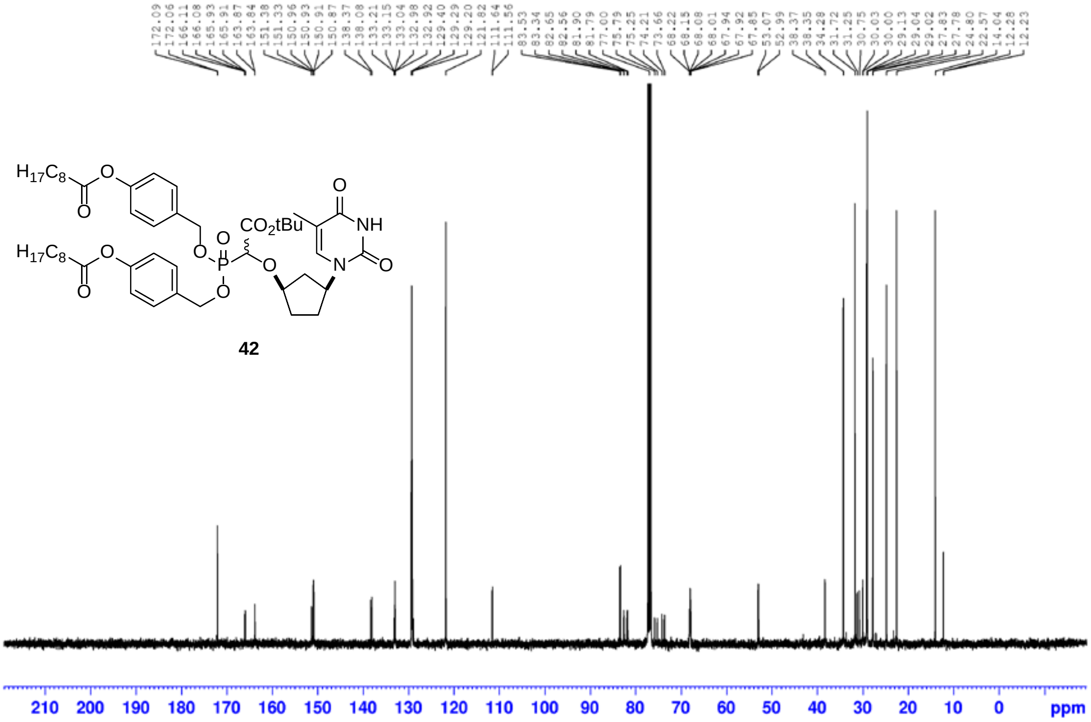

Compound **42** $^{31}P\{^{1}H\}$ NMR (162 MHz, $CDCl_3$)

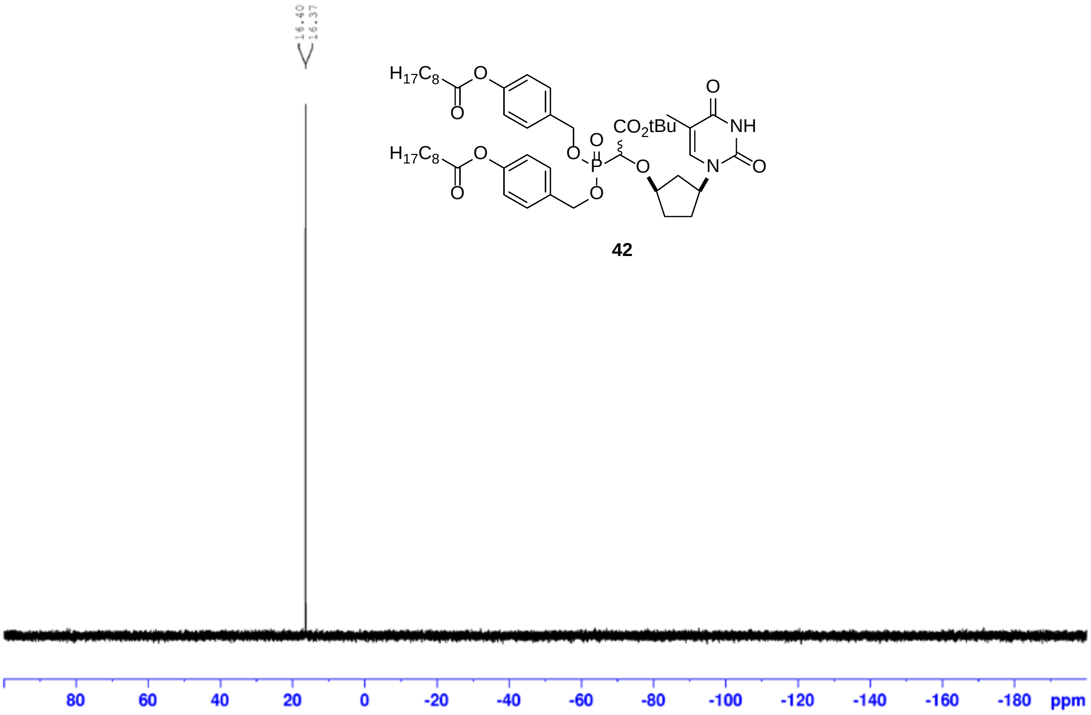

Compound **43** ^{1}H NMR (400 MHz, $CDCl_3$)

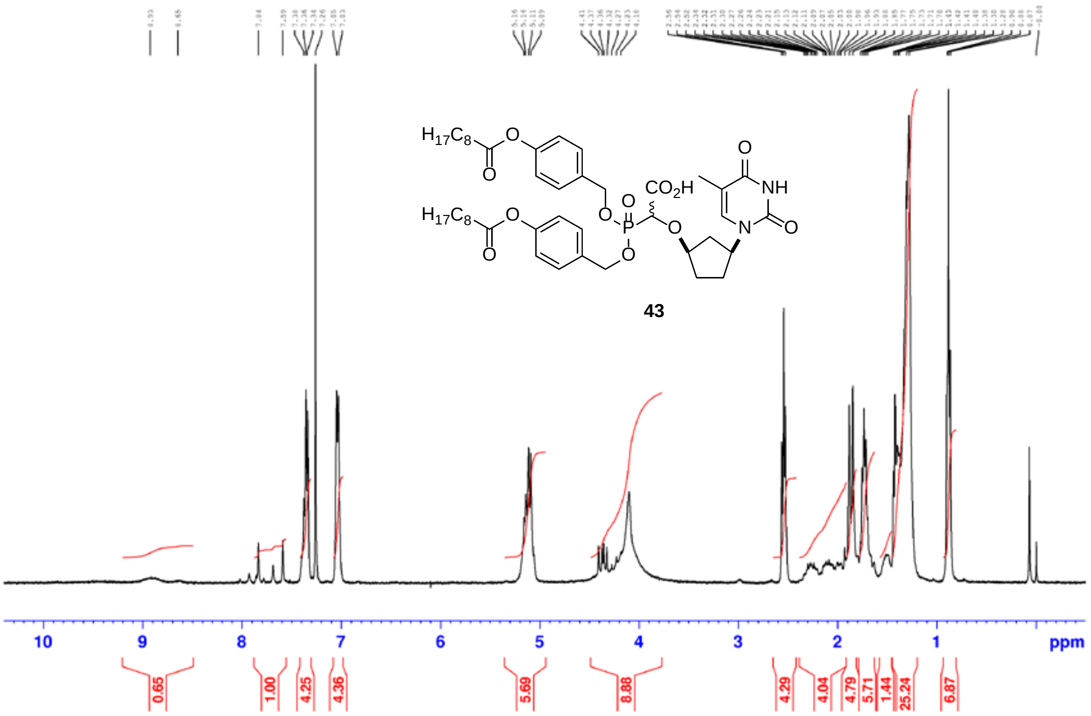

Compound **43** $^{31}P\{^{1}H\}$ NMR (162 MHz, $CDCl_3$)

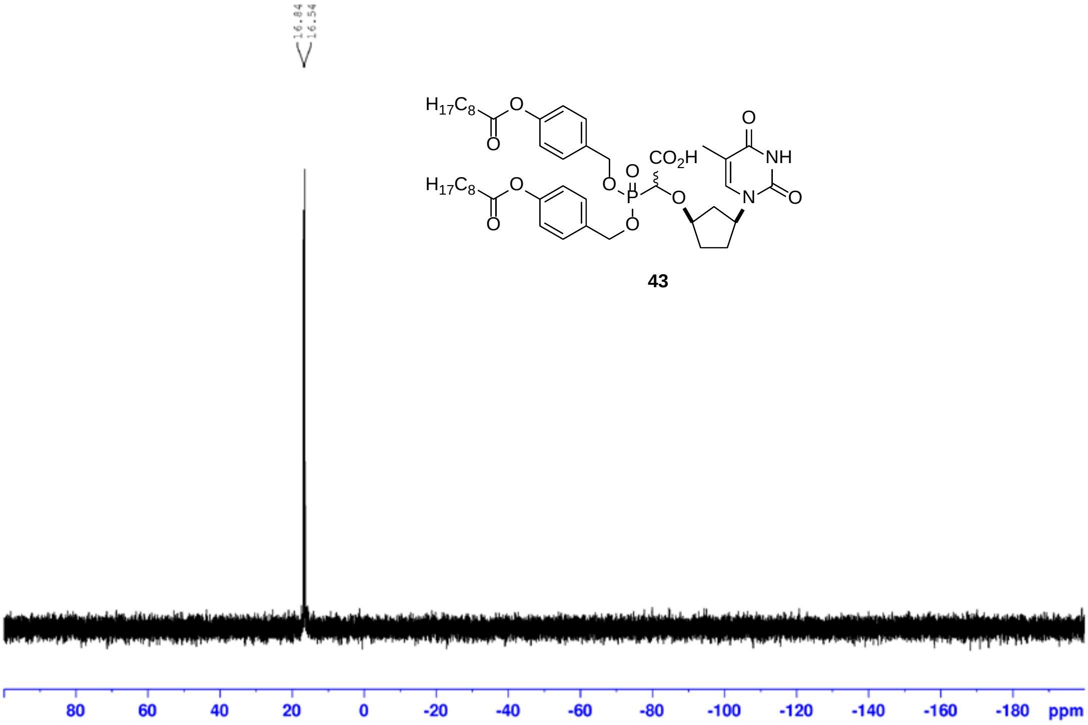

Supplement: Supplementary file 1 — jo2c02135_si_001.pdf [file jo2c02135_si_001.pdf]
